# Bone morphogenetic protein 4 inhibits rat stem/progenitor Leydig cell development and regeneration via SMAD-dependent and SMAD-independent signaling

**DOI:** 10.1038/s41419-022-05471-8

**Published:** 2022-12-13

**Authors:** Xiaoheng Li, Yinghui Fang, Lanlan Chen, Hehua Quan, Yiyan Wang, Ren-Shan Ge

**Affiliations:** 1 Department of Anesthesiology, The Second Affiliated Hospital and Yuying Children’s Hospital of Wenzhou Medical University, 109 Xueyuan West Road, Wenzhou, Zhejiang 325027 China; 2 Experimental Teaching of Clinical Skills Center, Academic Affairs Office, Wenzhou Medical University, Wenzhou, Zhejiang 325035 China; 3 Department of Anesthesiology, Taizhou People’s Hospital, Fifth Affiliated Hospital of Nantong University, Taizhou City, Jiangsu China

**Keywords:** Cell growth, Cell division

## Abstract

Bone morphogenetic protein 4 (BMP4) is an important member of the transforming growth factor-β superfamily. BMP4 is expressed in the Leydig cell lineage. We hypothesized that BMP4 might regulate the development of stem/progenitor Leydig cells. The BMP4 receptors, BMPR1A, BMPR1B, and BMPR2 were found to be expressed in progenitor Leydig cells of prepubertal testis and isolated cells. BMP4 at 1 and 10 ng/mL significantly reduced androgen production and down-regulated steroidogenesis-related gene and protein expression possibly by activating the SMAD signaling pathway (increasing SMAD1/5 phosphorylation and SMAD4) at 24 h treatment. BMP4 at 0.1 ng/mL and higher concentrations markedly reduced the EdU labeling index of CD90^+^ stem Leydig cells after 24 h treatment and significantly reduced the number of EdU^+^ stem Leydig cells on the surface of seminiferous tubules after 7 days of culture. BMP4 at 0.01 ng/mL and higher concentrations significantly blocked the differentiation of stem Leydig cells into adult cells, as shown by the reduction of testosterone secretion and the downregulation of *Lhcgr, Scarb1, Cyp11a1, Hsd11b1*, and *Insl3* and their function after 3D seminiferous tubule culture for 3 weeks, and this effect was reversed by co-treatment with the BMP4 antagonists noggin and doxomorphine. In addition, BMP4 also blocked stem Leydig cell differentiation through SMAD-independent signaling pathways (ERK1/2 and AMPK). Ethanedimethane sulfonate (EDS) single injection can result in reduction of testosterone, restoration can happen post treatment. In an in vivo model of Leydig cell regeneration following EDS treatment, intratesticular injection of BMP4 from day 14 to day 28 post-elimination significantly reduced serum testosterone levels and down-regulated the expression of *Scarb1, Star, Hsd11b1,* and *Insl3* and its proteins, possibly through SMAD-dependent and SMAD-independent (ERK1/2 and AMPK) signaling pathways. In conclusion, BMP4 is expressed in cells of the Leydig cell lineage and blocks entry of stem/progenitor Leydig cells into adult Leydig cells through SMAD-dependent and SMAD-independent signaling pathways.

## Introduction

The adult mammalian (rat) testis is divided into two compartments: the seminiferous tubule (ST) and the interstitium, which contains the adult Leydig cell (ALC) and its founder stem Leydig cells (SLCs). The main function of ALCs is to synthesize testosterone (T), which plays a key role in maintaining secondary sexual characteristics and promoting spermatogenesis [1, 2], and secrete insulin-like 3 (INSL3) to maintain bone health [3, 4].

T biosynthesis requires stimulation of luteinizing hormone (LH), which binds to LH receptors (LHCGR) on ALC surface to initate its signal [4]. T is made from the substrate cholesterol, which is mainly taken up by the high-density lipoprotein receptor (SCARB1). In ALCs, cholesterol is acutely transported to mitochondria via the acute regulator of steroidogenesis (STAR), where it is catalyzed by cholesterol side-chain lyase (CYP11A1) in the mitochondria to produce pregnenolone, which is further catalyzed on the smooth endoplasm to be converted to T by a series of 3β-hydroxysteroid dehydrogenase 1 (HSD3B1), 17α-hydroxylase/17,20-lyase (CYP17A1) and 17β-hydroxysteroid dehydrogenase 3 (HSD17B3) catalysis. T can be converted to the more potent androgen dihydrotestosterone (DHT) by 5α-reductase 1 (SRD5A1) in progenitor Leydig cells (PLCs) and immature Leydig cells (ILCs) during development, which in turn is converted to 3α-hydroxysteroid steroids (e.g., androsterone in PLCs) by 3α-hydroxysteroid dehydrogenase (AKR1C14) [4]. In rat model, morphologically, PLCs appear SLC-like, but biological function analysis shows that PLCs begin to express CYP11A1, HSD3B1, and CYP17A1 at a low level without expressing HSD17B3, and mainly secrete androsterone [5]. PLCs further undergo a series of differentiations, finally to ALCs [4, 5]. The ALC development can be investigated both in vivo using ethanedimethane sulfonate (EDS)-induced ALC elimination to induce a regeneration process and in vitro using EDS-treated 3D ST culture system [6, 7]. Once adult male rats are intraperitoneally injected with 75 mg/kg EDS, the drug can induce ALC apoptosis [8]. ALCs disappear within 7 days and subsequent regeneration of ALCs is very similar to its pubertal development, in which SLCs commit to PLCs, finally into ALCs which express mature cell biomarker 11β-hydroxysteroid dehydrogenase 1 (HSD11B1), a glucocorticoid-metabolizing enzyme, on post-elimination day 56 [4].

Although LH has been shown to play a critical role in ALC development from SLCs, initial development of SLCs into the LC lineage does not require LH [9]. However, other growth factors may be needed for the initial commitment and development of SLCs [10]. Our previous studies have been able to identify SLCs on the surface of STs [6, 7], and they express several biomarkers including platelet-derived factor receptor A (PDGFRA) [11], Nestin [11, 12], CD90 [7], and CD51 [12]. Being stem cells, SLCs not only have self-renewal properties, but also they can differentiate into ALCs [4]. A growing number of studies are attempting to identify SLC their regulators [4]. One of the underlying factors may be bone morphogenetic protein 4 (BMP4), a member of the transforming growth factor-β (TGFβ) family. In this study, we re-analyzed the microarray data for BMP family member in the LC lineage and compared to bone marrow-derived mesenchymal stem cell (BMSC) and found that BMP4 was the predominant BMP member, increasing significantly from SLCs to PLCs. However, the role of BMP4 in LC development, especially at the early development stages, remains unclear. BMP4 primarily activates the type I receptors, BMPR1A and BMPR1B, and then type II BMPR2 [13], to form ligand-receptor complexes that enter cells to phosphorylate receptor-associated SMAD (R-SMAD) proteins such as SMAD1 and SMAD5 [13]. R-SMADs form complexes with co-operating SMAD (co-SMAD, SMAD4) to regulate gene transcription in the nucleus [14]. In this study, we investigated the effect of BMP4 on SLC/PLC development and entry into the LC lineage and explored the underlying mechanisms.

## Results

### Detection of BMP signaling in the LC lineage

**Fig. 1:**
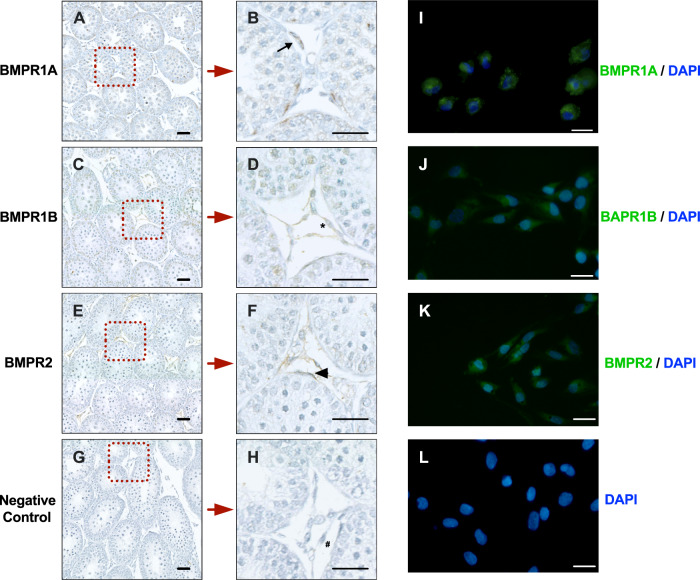
Detection of BMP4 receptors in rat testis and progenitor Leydig cells (PLCs). **A**–**F** Immunohistochemistry of BMPR1A, BMPR1B, and BMPR2 in cross-sections of 21-day-old rat testis (black arrow: BMPR1A^+^; asterisks: BMPR1B^+^, black arrowhead: BMPR2^+^. **G**, **H** Negative control (#: Leydig cells). Scale bar = 100 μm. **I**–**L** Immunofluorescence of BMPR1A, BMPR1B and BMPRII in PLCs. **H** Negative control. Scale bar = 20 μm.

To determine whether BMP ligands have autocrine regulation on LC development, we re-analyzed previous arrays of SLCs, FLCs, ILCs, ALCs, and BMSCs [15]. As shown in Fig. S1. Three BMP ligands (*Bmp3*, *Bmp4,* and *Bmp6*) were expressed in the LC lineage; *Bmp3* levels was about 60 units in SLC, were low in PLC, ILC, and ALC and close to 100 units in BMSC. *Bmp4* levels in all cell types were well below 100 units. *Bmp6* levels were showed an increasing trend across the cell types-low in SLC and highest in ALC and very low levels in BMSC. The levels of *bmp3* and *4* are low but high in *bmp6*. This result indicates that the expression trends of *Bmp4* and *Bmp6* are opposite. In this study, we focused on BMP4 and its signaling. BMP4 ligand dimers exert signaling effects by promoting the assembly of heteromeric complexes of BMP type I (BMPR1A and BMPR1B) and type II receptors (BMPR2) at the cell surface. Since PLCs are mainly present in prepubertal rat testis (21-day-old), we performed immunohistochemistry for BMPR1A, BMPR1B, and BMPR2 in 21-day-old rat testis. BMPR1A, BMPR1B, and BMPR2 were all detected in 21-day-old rat testis, and they were mainly expressed in PLCs, SCs, and spermatocytes (Fig. 1A–H). We then purified PLCs and performed immunofluorescence detection of these receptors in PLCs, all of which were present in isolated PLCs (Fig. 1I–L), indicating that PLCs are the target of BMP4.

### BMP4 inhibits PLC differentiation in vitro

**Fig. 2:**
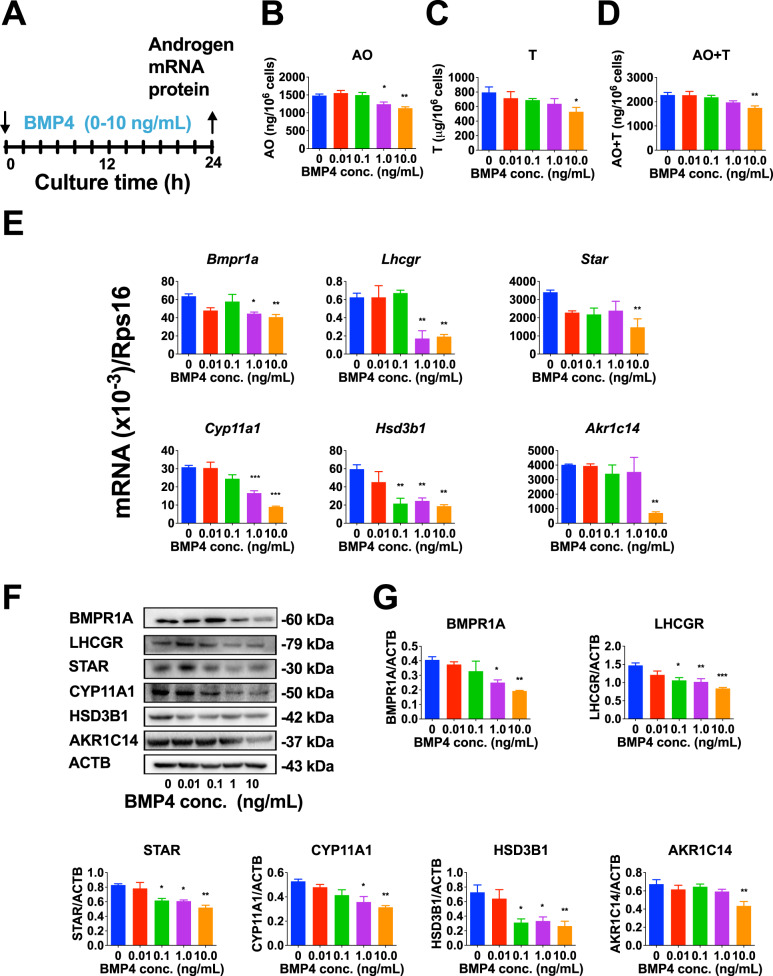
BMP4 inhibits progenitor Leydig cell (PLC) differentiation in vitro. **A** PLCs were incubated with BMP4 for 24 h. **B**–**D** Androsterone (AO), T, AO + T output after BMP4 treatment; Mean ± SEM, *n* = 4. **E** QPCR detection of *Bmpr1a*, *Lhcgr*, *Star*, *Cyp11a1*, *Hsd3b1*, and *Akr1c14* expression which was normalized to *Rps16* (an internal control); Mean ± SEM, *n* = 4. **F** Western blot. **G** Quantitative results of BMPR1A, LHCGR, STAR, CYP11A1, HSD3B1, and AKR1C14 protein levels which were normalized to ACTB (an internal control); Mean ± SEM, *n* = 3. *P < 0.05, **P < 0.01 and ***P < 0.001 indicate significant differences compared to control (0 ng/mL BMP4).

Since the expression of *Bmp4* was significantly downregulated from PLCs to ALCs (Fig. S1), we hypothesized that BMP4 might inhibit PLC differentiation into ALCs. Primary PLCs were incubated with a range of concentrations of BMP4 (0, 0.01, 0.1, 1.0, and 10.0 ng/mL) for 24 h (Fig. 2A). PLCs mainly secrete the major androgen androsterone (AO) and the minor androgen T [5]. We measured the output of these androgens in PLCs following BMP4 treatment. BMP4 significantly reduced the output of AO, T, and AO + T levels (Fig. 2B–D), indicating that BMP4 blocks PLC steroidogenesis. To dissect which points are affected by BMP4, we performed qPCR on a series of mRNAs associated with steroidogenesis and its regulation. We first explored whether BMP4 affected the expression of BMP4 receptors (*Bmpr1a*, *Bmpr1b*, and *Bmpr2*) and found that BMP4 markedly downregulated *Bmpr1a* expression (Fig. 2E) without affecting *Bmpr1b* and *Bmpr2* expression (Fig. S2). Then, we examined the expression of genes in the steroidogenic cascade. BMP4 significantly downregulated the expression of *Lhcgr*, *Star*, *Cyp11a1*, *Hsd3b1*, and *Akr1c14* (Fig. 2E), whereas the expression of *Scarb1*, *Srd5a1*, and *Nr5a1* was unchanged (Fig. S2). Western blot revealed that BMP4 significantly reduced BMPR1A, LHCGR, STAR, CYP11A1, HSD3B1, and AKR1C14 levels (Fig. 2F, G). These data indicate that BMP4 blocks PLC differentiation by downregulating the expression of steroidogenesis-related genes and their proteins, and the downregulation of *Bmpr1a* may be due to a feedback protective mechanism.

### BMP4 inhibits PLC differentiation through the SMAD pathway

**Fig. 3:**
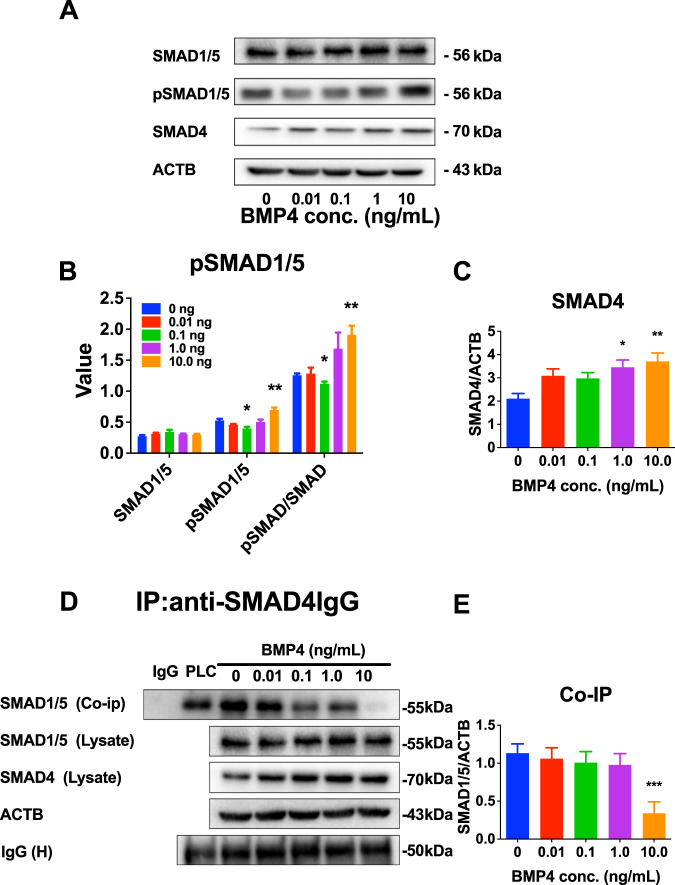
BMP4 acts via the SMAD pathway in progenitor Leydig cells (PLCs) in vitro. **A** Western blot. **B** Quantitative protein levels of pSMAD1/5, SMAD1/5, and their ratio; Mean ± SEM, *n* = 3. **C** Quantitative levels of SMAD4; Mean ± SEM, *n* = 5. **D** Western blot for Co-P proteins; **E** Quantitative data of Co-IP protein levels, Mean ± SEM, *n* = 6. *P < 0.05 and **P < 0.01 indicate significant differences compared to control (0 ng/mL BMP4).

Following BMP4 ligand binding, type II receptors typically phosphorylate and activate type I receptors, which in turn phosphorylate and activate receptor-associated SMADs (R-SMAD) [14]. R-SMADs form complexes with co-operating SMAD (co-SMAD, SMAD4) to regulate gene transcription in the nucleus [14]. Typically, BMP4 recruits SMAD1 and SMAD 5 (SMAD1/5) [14]. To dissect whether BMP4 transduces R-SMAD signaling, SMAD1/5, pSMAD1/5, and SMAD4 levels were measured in PLCs treated with BMP4 for 24 h. BMP4 did not alter total SMAD1/5 levels in PLCs, but significantly increased pSMAD1/5 at 10.0 ng/mL, resulting in similar changes in the ratio of pSMAD1/5 to SMAD1/5 (Fig. 3A, B). BMP4 at 1 and 10 ng/mL also significantly increased SMAD4 levels (Fig. 3A, C). Previous studies have shown that pSMAD1/5 forms a complex with SMAD4 to regulate gene transcription [14]. Co-IP was used for the interaction between pSMAD1/5 and SMAD4. The results showed that BMP4 at 10 ng/mL decreased total SMAD1/5 after Co-IP with SMAD4, indicating that the relative SMAD4-Co-IPed pSMAD5 is increased (Fig. 3D, E). These results indicate that BMP4 regulates PLC differentiation by activating the SMAD pathway.

### BMP4 inhibits SLC proliferation in vitro

**Fig. 4:**
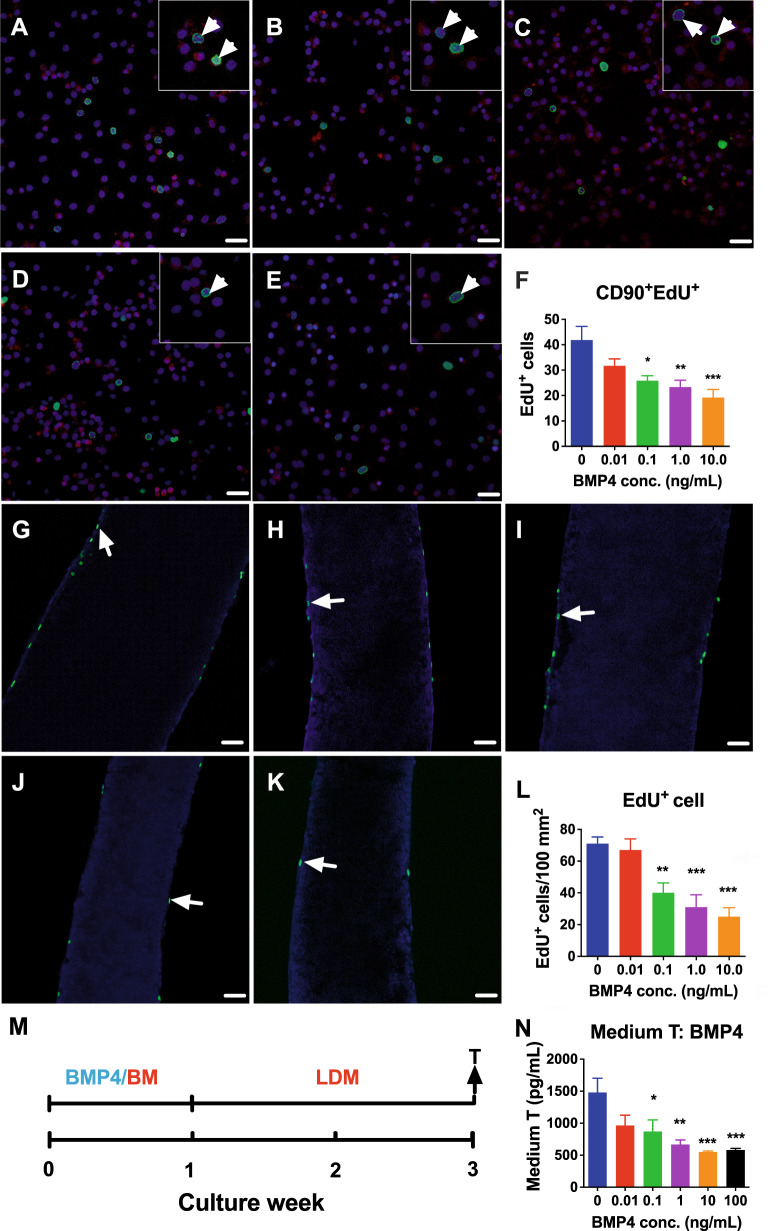
The effect of BMP4 on stem Leydig cell (SLC) proliferation in vitro. **A**–**F** CD90^+^ SLCs were incubated with 0 (**A**), 0.01 (**B**), 0.1 (**C**), 1.0 (**D**), and 10.0 (**E**) ng/mL BMP4 for 24 h. **F** Quantitative data for EdU labeling index, mean ± SEM, *n* = 6. White arrowheads indicate EdU^+^ SLCs: CD90, red membrane; EdU, green nucleus; DAPI, blue nucleus. Bar = 50 μm. SLCs on ST seminiferous tubule (ST) surface treated with 0 (**G**), 0.01 (**H**), 0.1 (**I**), 1.0 (**J**), and 10.0 (**K**) ng/mL of BMP4 for 7 d and then EdU (green) was incorporated; **L** Quantitative results for EdU^+^ SLCs per square centimeter, mean ± SEM, *n* = 6. White arrows indicate EdU^+^ SLCs. Bar = 50 μm. **M** ST culture protocol; **N** Medium testosterone (T) levels after BMP4 treatment in the first week; Mean ± SEM, *n* = 6. *P < 0.05, **P < 0.01, and ***P < 0.001 indicate significant differences compared to control (0 ng/mL BMP4).

*Bmp4* was also expressed in SLCs (Fig. S1). Previous studies have shown that CD90^+^ cells on the surface of STs are SLCs [7, 16]. To explore whether BMP4 had an effect on SLC proliferation. We purified CD90^+^ SLCs (characterized in Fig. S7C, D), incubated with BMP4 for 24 h, and used EdU incorporation into SLCs to determine labeling index. BMP4 at 0.1 ng/mL and higher concentrations significantly reduced the ability of EdU to incorporate into CD90^+^ SLCs compared to the control (Fig. 4A–F). We also treated STs with BMP4 to observe the incorporation of EdU into SLCs on the ST surface as previously described [17] and found that BMP4 as low as 0.1 mg/mL significantly reduced EdU^+^ SLC number on the ST surface (Fig. 4G–L). Our previous study used a pretreatment method in basal medium (BM) for 1 week to increase the number of SLCs by growth factors, and then treated STs for SLC differentiation into ALCs for secreting T to judge SLC proliferation in the 3D ST culture system [7]. In this method, STs were treated with BMP4 (0, 0.01, 0.1, 1.0, 10.0, and 100.0 ng/mL) in BM, then in the second week, STs were switched to LC differentiation medium (LDM) without BMP4 for 2 weeks, and at the end of treatment, medium T was measured (Fig. 4M). BMP4 significantly reduced T levels in a dose-dependent manner (Fig. 4N), indicating a reduction in the number of SLCs during the first week of BMP4 treatment. To support this assertion, we measured LC steroidogenesis-related gene and protein expression and showed that BMP4 at 1.0 ng/mL significantly downregulated the expression of *Lhcgr*, *Scarb1*, *Hsd11b1* and *Insl3* (Fig. S3), indicating that BMP4 reduces SLC proliferation.

### BMP4 inhibits SLC differentiation in vitro

**Fig. 5:**
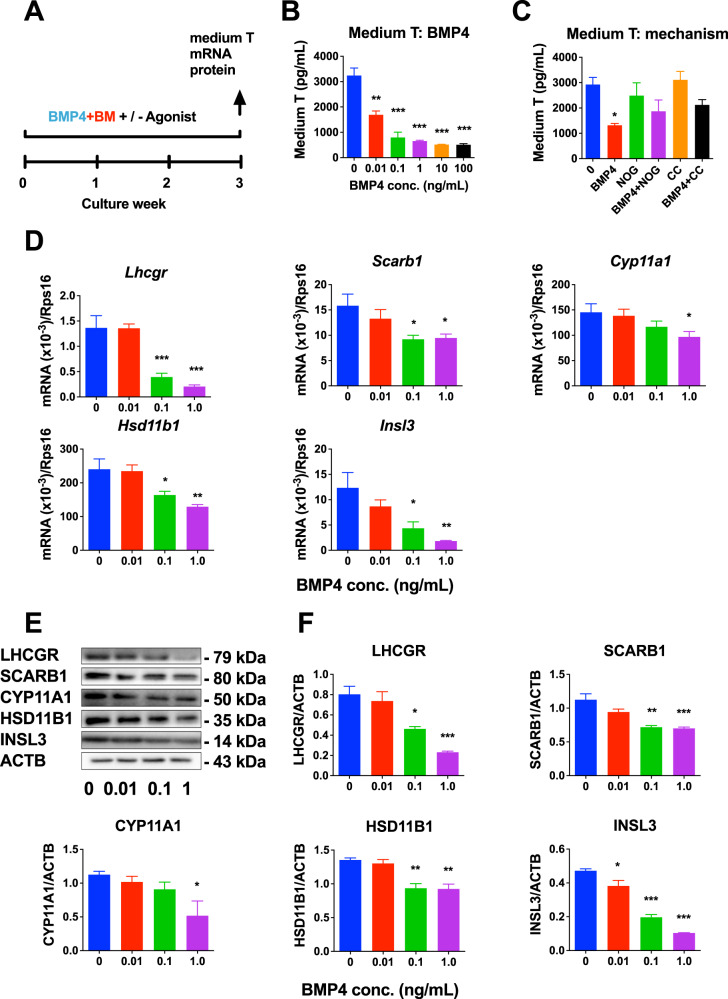
Effects of BMP4 on stem Leydig cell (SLC) differentiation in vitro. **A** Seminiferous tubule (ST) culture protocol. **B** Medium testosterone (T) levels after BMP4 treatment; Mean ± SEM, *n* = 6; **C** Medium T with 1 ng/mL BMP4 with or without NOG (1 ng/mL) or CC (5 ng/mL). Mean ± SEM, *n* = 5; **D** *Lhcgr, Scarb1, Cyp11a1, Hsd11b1*, and *Insl3* mRNA levels in STs treated with BMP4 for 3 weeks by qPCR and normalized to *Rps16*; Mean±SEM, *n* = 6. **E** Western blot. **F** Protein levels of LHCGR, SCARB1, CYP11A1, HSD11B1, and INSL3 in STs treated with BMP4 and normalized to ACTB; Mean ± SEM, *n* = 3. *P < 0.05, **P < 0.01, and ***P < 0.001 indicate significant differences compared to control (0 ng/mL BMP4).

Previous studies have shown that SLCs on the ST surface can be induced to differentiate into ALCs after 3 weeks of induction in LDM [7]. We treated STs with BMP4 in LDM for 3 weeks and measured medium T levels at the end of the third week (Fig. 5A). BMP4 at 0.01 ng/mL and higher concentrations markedly reduced T levels in a dose-dependent manner (Fig. 5B). The half maximum inhibitory concentration (IC_50_) of BMP4 in SLC differentiation was 17.57 nM. To dissect the mechanism of BMP4, we used two BMP4 antagonists, noggin (NOG) and dorsorphin (CC). The results showed that NOG or CC alone did not affect T output (Fig. S4A). However, NOG and CC completely reversed T reduction incubated with 1.0 ng/mL BMP4, which markedly inhibited T biosynthesis (Fig. 5C). We further examined the mRNA levels in the steroidogenic pathway of *Lhcgr*, *Scarb1*, *Star*, *Cyp11a1*, *Hsd3b1*, *Hsd17b3*, *Hsd11b1*, and *Insl3*. We found that BMP4 downregulated *Lhcgr*, *Scarb1*, *Cyp11a1*, *Hsd11b1*, and *Insl3* expressions (Fig. 5D), and BMP4 had no effect on *Star*, *Cyp17a1*, and *Hsd3b1* expression (Fig. S4B). We analyzed protein levels by Western blot. The results showed that the protein levels of LHCGR, SCARB1, CYP11A1, HSD11B1, and INSL3 were significantly reduced by BMP4 compared to the control (0 ng/mL BMP4) (Fig. 5E, F). This was consistent with corresponding mRNA changes.

### BMP4 inhibits SLC differentiation through SMAD-independent pathway

**Fig. 6:**
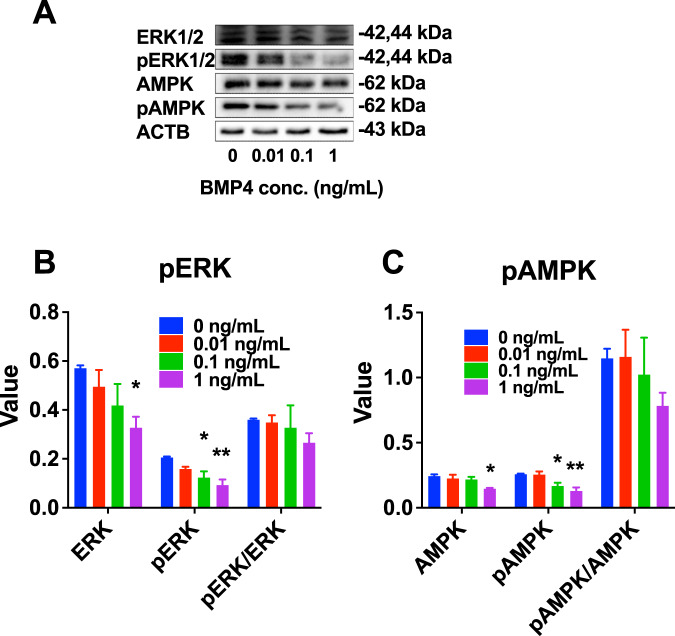
Kinase and phosphorylated kinase protein levels after 3-week BMP4 treatment to seminiferous tubules (STs) in vitro. **A** Western blot; **B**, **C** Quantitative data of pERK1/2, ERK1/2, pAMPK, AMPK, and their ratio. Mean ± SEM, *n* = 3. *P < 0.05 and **P < 0.01 indicate significant differences compared to control (0 ng/mL BMP4).

In PLC differentiation, we already demonstrated that BMP4 exerted an action via classic SMAD pathway. Previous studies have also shown that TGFβ/BMPs can activate R-SMAD-independent pathways, including mitogen-activated protein kinase (MAPK) pathways such as extracellular signal-regulated kinases 1 and 2 (ERK1/2) [18] and AMP-activated kinase (AMPK) pathways [19]. BMP-activated MAPK family members rely on adaptor proteins to bridge receptors for ERK1/2 [18]. We further measured ERK1/2 and AMPK signaling in BMP4-treated STs and results showed that BMP4 significantly reduced total ERK1/2 and AMPK and phosphorylated ERK1/2 (pERK1/2) and pAMPK levels, although the pERK/ERK1/2 and pAMPK/AMPK ratios did not change (Fig. 6A–C).

### BMP4 inhibits LC regeneration in the EDS model

**Fig. 7:**
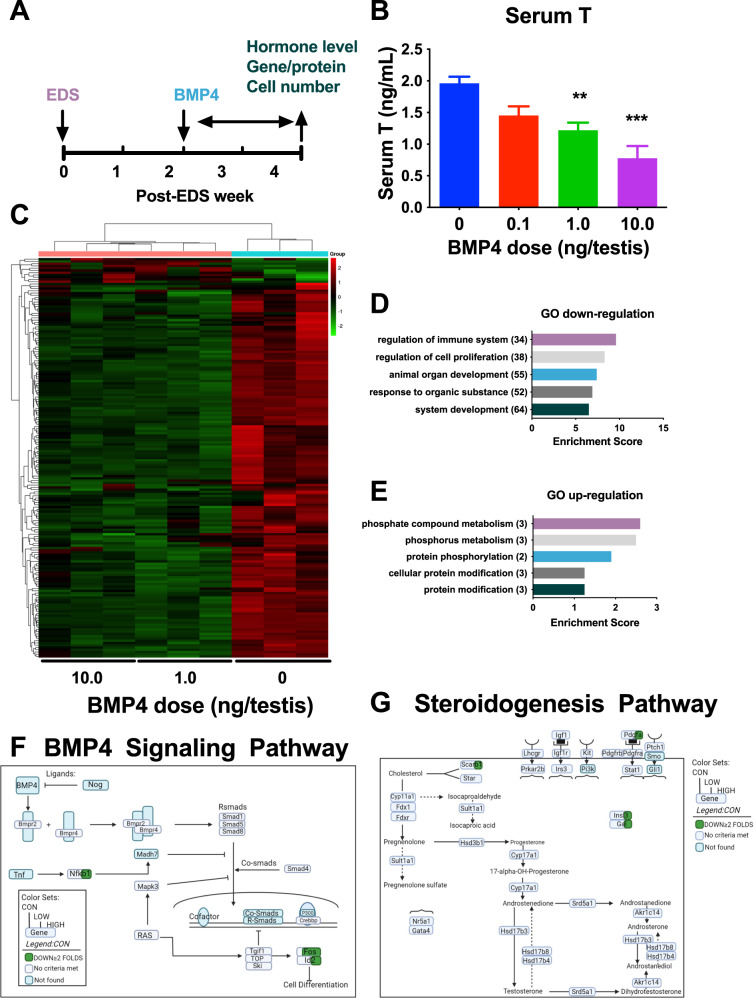
Serum testosterone (T) levels and RNA-seq analysis BMP4-treated testis in vivo. **A** Animal protocol i.t. of 0, 0.1, 1, and 10 ng/testis BMP4 on day 14 to day 28 after EDS (ethanedimethane sulfonate); **B** Serum T levels, Mean ± SEM, *n* = 6; **C** mRNA HeatMap between BMP4 (1 and 10 ng/testis) and control (0 ng/testis) samples; red indicates upregulated genes, green indicates downregulated genes, mean ± SEM, *n* = 3; **D** downregulated gene GO, mean ± SEM, *n* = 3; **E** upregulated gene GO, mean ± SEM, *n* = 3; **F** BMP4 pathway; **G** steroidogenic pathway; Green indicates downregulated genes ≥2 fold, purple indicates genes not found. **P < 0.01 and ***P < 0.001 indicate significant differences compared to control (0 ng/testis BMP4).

Previous studies have shown that ALCs can regenerate from SLCs after complete elimination using the drug EDS [6, 20]. After EDS injection, ALCs disappeared completely, and some SLCs in the interstitium developed into PLCs starting from postnatal day 12–14. To explore the role of BMP4 in the progression of SLCs into PLCs and further into ILCs, rats were treated by intratesticular injection of BMP4 (0, 0.1, 1.0, and 10.0 ng/testis) at the reappearance of PLCs from day 14 to day 28 after EDS (Fig. 7A). Intratesticular administration of BMP4 was used to avoid systemic effects. After treatment, we found no effect of BMP4 on body weight, testicular and epididymal weights (Table S1). On post-EDS day 28, BMP4 at 1 and 10 ng/testis significantly reduced serum T levels, indicating decreased LC function (Fig. 7B). However, BMP4 did not alter serum LH and FSH levels (Fig. S5A), confirming no systemic effects.

### BMP4 reduces steroidogenesis without affecting LC number in the EDS model

**Fig. 8:**
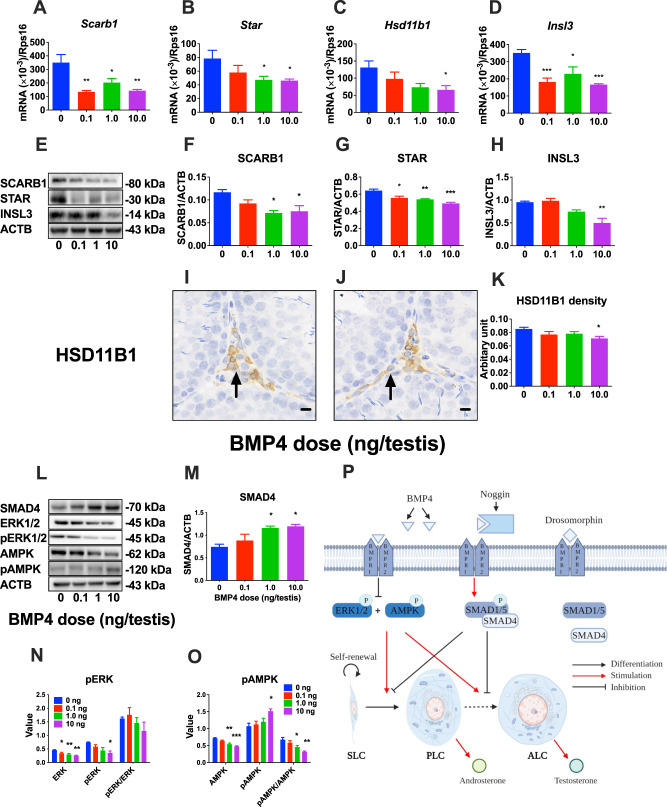
Gene and protein expression in Leydig cells after BMP4 treatment in vivo. **A**–**D** *Scarb1, Star, Hsd11b1*, and *Insl3* mRNAs in the testes of rats treated with 0, 0.1, 1.0, and 10.0 ng/testis BMP4 for 14 d after EDS were analyzed by qPCR and normalized to *Rps16*; Mean ± SEM, *n* = 6. **E**–**H** Protein levels of SCARB1, STAR, INSL3 were measured by Western blot and normalized to ACTB, Mean ± SEM, *n* = 3. **I**–**K** Semi-quantitative measurement of HSD11B1 density after in vivo BMP4 treatment (0 and 10 ng/testis). Black arrows indicate HSD11B1^+^. Mean ± SEM, *n* = 6, Bar = 50 μm. **L** Protein levels of kinase, phosphorylated kinase protein, and SMAD pathway protein levels after BMP4 treatment in vivo. (M-O) Quantitative data for SMAD4, ERK1/2, and AMPK, and their phosphorylated proteins as well as their ratio; Mean ± SEM, *n* = 3–4. *P < 0.05, **P < 0.01, and ***P < 0.001 indicate significant differences compared to control (0 ng/testis BMP4). **P** Graphic Abstract. Mechanism of BMP4 regulating the development of stem/progenitor Leydig cells into adult Leydig cells. BMP4 has SMAD-dependent signaling: it binds to the BMPR to form complex, activating BMP-SMAD signaling; and SMAD-independent signaling: it blocks ERK1/2 and AMPK phosphorylation; thereby leading to the delay of Leydig cell regeneration or development.

The amount of androgens depends on the number of LCs and their ability to produce steroids. A previous study showed that by day 28 after EDS injection, ILCs appeared in the interstitium, and they expressed the LC lineage biomarker CYP11A1 and the ILC biomarker HSD11B1 [21]. We immunohistochemically stained CYP11A1 (for LCs), HSD11B1 (for ILCs), and SOX9 (for SCs). Since SC number maintains stable after 21 days postpartum as previously reported [22], we used SC as a reference. Interestingly, CYP11A1^+^ LCs and HSD11B1^+^ ILCs were unchanged compared to controls (Fig. S6A–D), indicating that BMP4 reduces T biosynthesis without affecting LC number although BMP4 inhibited SLC proliferation. This confirmed that the regeneration of LCs is not from proliferating SLCs but from a pre-existing subset of committed SLCs [23]. To determine the mechanism of inhibition of T biosynthesis mediated by BMP4, we performed RNA-seq on intratesticular BMP4-treated testes. 14047 transcripts were identified in testes of three groups (0, 1, and 100 ng/testis BMP4). Among these transcripts, 23 were significantly upregulated (*P* < 0.05) and 192 were significantly downregulated (*P* < 0.05) (Fig. 7C). GO analysis revealed that most downregulated genes were related to immune system, cell proliferation, organ development, regulation of organic subspace and phylogeny (Fig. 7D, Table S2), and most upregulated genes were related to phosphate compound metabolism, phosphorus metabolism, protein phosphorylation, cellular protein modifications, and protein modifications (Fig. 7E, Table S3). Further pathway analysis revealed that the expression of 3 genes (*Nfkb1*, *Fos*, and *Id2*) in the BMP4 signaling pathway was downregulated ≥2 fold (Fig. 7F), and 4 genes (*Scarb1*, *Pdgfa*, *Insl3*, and *Gal*) involved in LC steroidogenesis and regulation were downregulated ≥2 fold (Fig. 7G). To validate the RNA-seq data, we performed qPCR. As shown in Fig. 8A–D, BMP4 significantly downregulated the expression of LC functional genes including *Scarb1*, *Star*, *Insl3*, and *Hsd11b1*, although *Star* and *Hsd11b1* did not achieve a 2-fold downregulation in the RNA-seq analysis. Other LC genes *(Lhcgr*, *Cyp11a1*, *Hsd17b3*, *Cyp17a1*, and *Hsd3b1*) and SC genes (*Sox9*, *Amh*, and *Fshr*) were not altered (Fig. S5C). This indicates that BMP4 delays LC regeneration targeting a specific set of LC functional genes. To determine whether LC function at the protein level was lower, we performed Western blot and found that SCARB1, STAR, and INSL3 levels were significantly reduced in BMP4-treated testes (Fig. 8E–H). We further measured HSD11B1 density in individual ILCs and compared it with other LC biomarkers CYP11A1 and SC biomarker SOX9 and showed that 10 ng/testis of BMP4 significantly reduced HSD11B1 density without affecting CYP11A1 density and SOX9 density, consistent with Western blot data (Fig. 8I–K, and Fig. S6E–G).

### BMP4 inhibits LC regeneration through the SMAD-dependent and SMAD-independent pathways

We measured BMP4 down-stream effector SMAD4 in the classic SMAD pathway (Fig. 8L, M). The results showed that BMP4 at 1 and 10 ng/testis significantly increased the levels of SMAD4, indicating that BMP4 regulates LC regeneration through the SMAD signaling pathway. Previous studies have also shown that TGFβ/BMPs can activate R-SMAD-independent pathways, including MAPK pathways such as ERK1/2 [18] and AMPK pathway [19]. We further measured ERK1/2 and AMPK signaling and results showed that BMP4 significantly reduced total ERK1/2 and phosphorylated ERK1/2 (pERK1/2) levels, although the pERK/ERK1/2 ratio did not change. BMP4 significantly decreased total AMPK while increasing phosphorylated AMPK (pAMPK) levels, thereby increasing the pAMPK/AMPK ratio (Fig. 8N, O). These data indicate that BMP4 also regulates ERK1/2 and AMPK signaling pathways.

## Discussion

ALCs developed from SLCs are the main source of male androgens. This study used CD90^+^ SLCs and 3D ST culture system and an in vivo EDS-treated LC regeneration model in rats to explore autocrine factors that play critical roles in regulating SLC/PLC development. The results indicate that BMP4 is an autocrine factor released by LC lineage cells and other testicular cells and it plays a key role in the progression of SLCs/PLCs to ALCs.

It has been established that a new generation of ALCs is regenerated after ALCs in the rat testis are eliminated following EDS administration [6]. This indicates that SLCs exist in the adult testis [6]. Previous studies have shown that cells attached to the ST surface are SLCs and they are CD90^+^ and are capable of differentiating into ALCs [7]. In a recent study using single-cell RNA-seq to identify SLCs in an EDS-treated rat testicular interstitium and found that interstitium contained a heterogeneous pool of mesenchymal stem cells and CD90 was the best marker for SLCs in the EDS model [23]. Growing efforts have been made to identify growth factors that regulate SLC proliferation and differentiation and it has been found that TGFβ, desert hedgehog, platelet-derived growth factor AA and BB, fibroblast growth factor 1, 2, and 16, activin A, and kit ligand can regulate SLC proliferation and differentiation (see review [4]). We hypothesized that BMP signaling might also be involved in the regulation of SLC development in the LC lineage as autocrine factors. BMP4 is a member of TGFβ family and it exerts a major influence male fertility since knockdown of BMP4 caused male infertility in mice. Microarray analysis also showed that BMP4 was expressed at the highest level in PLCs and declined during the progression to PLCs into ALCs (Fig.S1). As TGFβ1 has been reported to block SLC proliferation and differentiation in rats [7], as a member of the TGFβ family, BMP4 might have a similar effect.

Due to the highest expression of *Bmp4* in PLCs, which emerge as the first cell type in the LC lineage, we identified the BMP4 receptors in PLCs. Data revealed that PLCs contained all three BMP4 receptors (BMPR1A, BMPR1B, and BMPR2), and it can respond to BMP4 stimulation. We found that BMP4 can inhibit PLC differentiation through SMAD-dependent pathways after establishing ligand-receptor interactions. Although *Bmp4* expression levels in SLCs were slightly lower, SLCs were still responsive to BMP4 stimulation, thereby inhibiting SLC proliferation and differentiation through SMAD-dependent and SMAD-independent (ERK1/2 and AMAPK) pathways. To dissect BMP4 signaling, we used two BMP4 antagonists NOG and CC. BMP4-mediated inhibition on SLC differentiation was completely reversed by both NOG and CC. CC is a selective BMPR1 inhibitor that blocks BMP-mediated phosphorylation [24, 25] NOG is an extracellular antagonist of BMP that binds directly to BMP4 [26]. NOG binds to the BMP dimer by mimicking ligand-receptor binding, wrapping the BMP dimer and blocking the binding of BMP to its type I or type II receptors to inactive BMPs [26, 27]. In this study, both CC and NOG can reverse BMP4-mediated inhibition. The complete antagonism by NOG and CC to BMP4 actions indicates that BMP4 exerts inhibitory effects on SLC development.

BMP4 clearly blocked SLC proliferation using two models: SLCs on ST surface and CD90^+^ SLCs. BMP4 significantly inhibited EdU incorporation into SLCs on ST surface and reduced EdU labeling index of CD90^+^ SLCs. However, we did not observe the change of LC number in the in vivo EDS-treated LC regeneration model, indicating that the proliferative SLCs after EDS treatment does not differentiate into LC lineage. It is true that very few of the newly differentiated LCs originated from the early dividing SLCs after single-cell RNA-seq analysis [23]. It is well known that highest proliferation occurs in EDS-treated testis within the first week [28] and labeling the proliferative SLCs using EdU for tracing for 3 weeks and very few PLCs that contained EdU co-staining, indicating that the cells differentiating to the LC lineage are not the dividing cells following EDS treatment but are possibly from pre-existed committed SLCs [23].

Following BMP4 ligand binding, type II receptors typically phosphorylate and activate type I receptors, which in turn phosphorylate and activate receptor-associated SMADs (R-SMAD) [14]. R-SMADs form complexes with co-operating SMAD (co-SMAD, SMAD4) to regulate gene transcription in the nucleus [14]. Typically, BMP4 recruits SMAD1 and SMAD 5 (SMAD1/5) [14]. To dissect whether BMP4 transduces R-SMAD signaling, SMAD1/5, pSMAD1/5, and SMAD4 levels were measured in PLCs treated with BMP4 for 24 h. BMP4 did not alter total SMAD1/5 levels in PLCs, but significantly upregulated pSMAD1/5 at high concentrations (10.0 ng/mL), resulting in similar changes in the ratio of pSMAD1/5 to SMAD1/5 (Fig. 3A, B). BMP4 also significantly increased SMAD4 levels (Fig. 3A, C). Previous studies have shown that SMAD1/5 forms a complex with SMAD4 to regulate gene transcription [14]. Co-IP was used for the interaction between SMAD1/5 and SMAD4. The results showed that SMAD1/5 and SMAD4 formed a complex to exert biological activity, and BMP4 at 10.0 ng/mL significantly reduced total SMAD1/5 Co-IP with SMAD4, indicating that the relative pSMAS1/5 is increased (Fig. 3D, E). These results indicate that BMP4 regulates PLC differentiation by activating the SMAD pathway.

In PLC differentiation, we already demonstrated that BMP4 exerted an action via classic SMAD pathway. Previous studies have also shown that TGFβ/BMPs can activate R-SMAD-independent pathways, including MAPK pathways such as ERK1/2 [18] and AMPK [19]. BMP-activated MAPK family members rely on adaptor proteins to bridge receptors for ERK1/2 [18]. We further measured ERK1/2 and AMPK signaling in BMP4 treated STs and results showed that BMP4 significantly reduced total ERK1/2 and AMPK and pERK1/2 and pAMPK levels. Studies have shown that ERK1/2 and AMPK pathways are involved in LC development [29]. Epidermal growth factor (EGF), insulin-like growth factor 1, and LH can regulate ERK1/2 or AMPK [30–32]. When ERK1/2 is phosphorylated and it activates steroid hormone biosynthesis by activating STAR, a key protein for cholesterol transport to the mitochondrial inner membrane [33, 34]. Indeed, the decrease of ERK1/2 phosphorylation may lead to the decrease of STAR after BMP4 treatment. Studies have shown that AMPK downregulates cAMP and it is related to the activity and gene expression of steroid enzyme promoters such as STAR, HSD3B1, and CYP17A1 [35, 36].

The normal ALC development and regeneration require an adequate amount of BMP4 for controlling SLC/PLC development. Knowledge regarding signaling pathways that regulate the development of different populations of cells in the LC lineage is still incomplete. This study is the first to (1) identify BMP4 as an autocrine factor to regulate SLC/PLC development; (2) establish the molecular mechanism behind BMP4-mediated SLC/PLC differentiation.

In conclusion, the results from this study demonstrated the critical role of BMP4 as an autocrine factor to control SLC/PLC development. BMP4 inhibits the proliferation and differentiation of rat SLCs and the differentiation into PLCs, and down-regulates steroidogenesis-related gene and protein expression, thereby reducing androgen levels. This process may act through SMAD-dependent and SAMD-independent (ERK1/2-AMPK) signaling pathways (Fig. 8P).

## Materials and methods

### Chemicals and kits

BMP4 (Cat#No.120-05ET) and Noggin (NOG, Cat#No.120-10 C) were purchased from PeproTech Asia (Shanghai, China). Dorsomorphin (CC) 2HCl (Cat#No.S7840) was purchased from Selleck (Shanghai, China). EDS was a gift from Jinan University (Guangzhou, China). Click-iT EdU Imaging Kit and the culture media (M-199 and DMEM/F12) were purchased from Invitrogen (Carlsbad, CA). Immulite 2000 Total Testosterone Kit was purchased from Sinopharm Group Medical Supply Chain Services Co (Hangzhou, China). Androsterone Elisa Kit (Cat#No.LS-F24986) was purchased from LifeSpan BioSciences (Seattle, WA). All other reagents were obtained from Sigma-Aldrich (St. Louis, MO). Primer information was listed in Table S4 and antibody information was listed in Table S5.

### Animals

Male Sprague-Dawley rats were purchased from Shanghai Laboratory Animal Center (Shanghai, China). Forty adult (60-day-old) male Sprague Dawley rats were used for EDS-treated LC regeneration model and 3D ST culture. Ten neonatal (7-day-old) male rats were used for CD90^+^ SLC isolation. Eighty male prepubertal (21-day-old) rats were used to purify PLCs for primary culture. The animal procedure was approved by the Institutional Animal Care and Use Committee of Wenzhou Medical University (protocol number wydw2019-0204) and was performed in accordance with the Guide for the Care and Use of Laboratory Animals.

### Isolation of PLCs and BMP4 treatment in vitro

Rats (21-day-old) were euthanized by CO_2_ for isolation of PLCs as previously described [5]. All testes were removed, and STs were extruded into dissociation buffer containing 0.25 mg/mL collagenase-D and DNase (0.25 mg/mL), digested at 34 °C for 10–15 min and terminated by pre-chilled buffer. Digested cells were then filtered through two layers of nylon mesh (200 μm) and washed. Filtered cells were centrifuged at 250 × *g* for 10 min and cells were resuspended in 55% isotonic Percoll. PLC fractions were collected between densities of 1.064 and 1.070 g/mL after centrifugation at a density gradient of 15,000 × *g* for 45 min at 4 °C. Cells were resuspended in phenol red-free DMEM:F12 supplemented with 1 mg/mL BSA and the purity was judged by HSD3B1 staining method as described [5]. The purity of PLCs was more than 95% (Fig. S5A, B). PLCs were seeded in plates and incubated with different concentrations of BMP4 (0, 0.01–10.0 ng/mL) for 24 h. Culture medium were harvested for measurement of T and androsterone, and cells are washed with PBS and harvested for mRNA and protein analysis.

### Isolation of ST and 3D ST culture system

EDS (75 mg/kg) was intraperitoneally injected into 60-day-old male rats. After 4 days of EDS (all ALCs were eliminated), the rats were sacrificed, the testes were taken and STs were extruded into the M199 medium and separated with interstitium and rinsed for 3-4 times with M199 medium. STs were equally resuspended in a 12-well plate with basal medium (BM, containing M199) or LC differentiation medium (LDM, containing 10 ng/mL LH, insulin-transferrin-selenium). Different concentrations (0, 0.01–100.0 ng/mL) of BMP4 and its inhibitor NOG (1 ng/mL) or CC (5 ng/mL) were added, and the cells were cultured in a 37 ^o^C in CO_2_ incubator. Media were changed twice each week. Previous study has shown that EdU incorporated cells were SLCs [17]. SLC proliferation assay was performed as described [17]. Briefly, STs were treated with BMP4 in BM for 1 week, and EdU was added for 16 h to perform EdU incorporation at the end of first week, or STs were switched to LDM for 2 weeks for induction of LC differentiation to produce T. At the end of 3 weeks, medium was collected for measuring T levels and STs were collected for mRNA and protein determination.

### Purification of CD90^+^ SLCs and BMP4 treatment in vitro

CD90^+^ SLCs were purified and cultured as previously described [7]. STs were isolated from 7-day-old male rats and collagenase D was added to obtain testicular cells. Cells were incubated with CD90 antibody in BD IMag™ Buffer for 20 min on ice. Magnetic beads were then added to incubate with CD90 antibody and cells for 30 min. After washing, cells were detached by BD IMagnet™ for 10 min. CD90^+^ cells (SLCs) were collected (Fig. S5C, D). To study the proliferation of SLCs, CD90^+^ cells (1 × 10^4^ cells/well) in M199 medium were seeded in 12-well plates and incubated with different concentrations of BMP4 (0, 0.01–10.0 ng/mL) for 24 h. Cells were then washed with PBS and incubated with EdU for 16 h as previously described in the following section.

### In vivo LC regeneration model after EDS-induced ALC elimination

24 male rats (60-day-old) were raised in an animal center for 7 d. The rats were then randomly divided into 4 groups (A–D groups) based on body weight with 6 rats in each group. The investigator was not blinded to the group allocation during the experiment. Each rat was intraperitoneally injected with 75 mg/kg EDS. On post-EDS day 14, the rats were simultaneously given normal saline (as a control) and BMP4 (0.1, 1.0, and 10.0 ng/testis) by intratesticular injection (10 μL/testis) from rete testis. The intratesticular injection was verified by 0.4% Trypan Blue (Sigma) and the results showed that the dye only retained within interstitium of the whole testis (Fig. S8). No inflammation was found by RNA-seq analysis. After daily administration of BMP4 for 14 d, all rats were euthanized with CO_2_, and blood samples were collected to measure serum T, LH, and FSH concentrations. One testis was collected to extract total RNA for qPCR and protein for Western blot. The contralateral testis was immersed in Bouin’s solution for immunohistochemistry (IHC) and immunofluorescence (IF) staining.

### Chemiluminescent measurement of medium and serum T

Medium and serum T measurement was performed in accordance with clinical standards in the clinical chemistry department of our hospital as previously described [37]. T concentration was detected by Immulite2000 total T kit. Serum was thaw at room temperature, vortexed well, and centrifuged. Normal rat serum and blank medium were used as positive and negative controls. 350 μL medium or serum was used for testing. The total T was measured by the analyzer using the solid-phase competitive chemiluminescence method and the lowest limit of detection was 0.2 ng/mL, and the intra- and inter-assay coefficients of variation were both within 15%.

### ELISA measurement of medium androsterone (AO) level

Medium AO level was determined using an AO ELISA kit. Medium was mixed with assay diluent A solution and incubated at 37 °C for 60 min. Then detection reagent B was mixed and incubated at 37 °C for 30 min. Finally, substrate was added and reacted in the dark at 37 °C for 15–30 min and terminated by stop solution. The microplate reader parameters were set to 550 nm, and the calibration wavelength was 450 nm. The lowest limit of detection of AO was 0.2 ng/mL, and the intra- and inter-assay coefficients of variation were within 15%.

### EdU incorporation into SLCs

STs were cultured in BM containing various concentrations of BMP4 (0, 0.01–100.0 ng/mL) for 7 d, and CD90^+^ SLCs were cultured in DMEM:F12 containing various concentrations of BMP4 (0, 0.01–10.0 ng/mL) for 24 h. EdU (1:1000) was added and incubated in the dark for 24 h. ST or CD90^+^ SLCs were washed with 3% BSA, then fixed with 4% paraformaldehyde and stained with reagents as described [7]. Photographs were taken with an Olympus fluorescence microscope (Olympus, Japan). EdU-positive cells were counted by Image ProPlus 6.0 software (Media Cybernetics, USA). At least 200 CD90 + SLCs were counted, and of which EdU^+^ CD90^+^ SLCs were counted and calculated the percentage of EdU^+^ SLCs in total CD90^+^ SLCs as the EdU labeling index. For STs, EdU^+^ cells were counted and EdU^+^ cells per mm^2^ of the ST area were calculated.

### Serum FSH and LH concentration analysis

LH and FSH levels were determined using ELISA kits as previously described [38]. Serum samples and assay solutions were mixed and incubated at room temperature for 2 h. The peroxidase-conjugated IgG anti-LH or anti-FSH solution was then mixed and incubated at room temperature for 2 h. Substrate buffer was added and the plate was stored in the dark for 30 min and terminated. The microplate reader parameters were set to 550 nm, and the calibration wavelength was 450 nm. The sensitivities of the FSH and LH were 1 ng/mL and 0.1 ng/mL, respectively. The intra- and inter-assay CVs were within 10% for both hormones.

### Dehydration, embedment, and testis-tissue array preparation

Twelve tests were taken from each group and fixed in Bouin’s solution for 24 h. A testis-tissue array was prepared for IHC and IF experiments. One testis per rat was selected, cut into 8 discs, and two pieces were randomly selected. These two discs were further cut into 4 pieces each and one piece was randomly selected from these 8 pieces. The piece of testis was embedded in paraffin in a tissue array container. After the conventional dehydration and embedding process, 5–6 μm thick sections were cut for IHC and IF experiments.

### Immunohistochemistry and Immunofluorescence

For IHC, the slices were baked for 2 h, dewaxed, and rehydrated, and 3% H_2_O_2_ was added to block the endogenous peroxidase. Three antibodies, CYP11A1 (LC biomarker), HSD11B1 (ILC biomarker) and SOX9 (SC biomarker) polyclonal antibodies, were used. Diaminobenzidine solution was added to show the brown color of the target protein. Counterstaining was Mayer hematoxylin. Non-immune rabbit IgG was used as the negative control. To determine BMP4 receptors, BMPR1A, BMPR1B, and BMPRII antibodies were used for IHC in 21-old rat testis. HRP-conjugated secondary antibody ligation, diaminobenzidine staining and Mayer hematoxylin counterstaining were performed as above. For IF staining of BMPR1A, BMPR1B, and BMPRII, PLCs were isolated from 21-day-old male rats as above and grown on glass coverslips. Cell fixation with 4% paraformaldehyde, non-specific antigen blocking with 1% donkey serum, BMPR1A, BMPR1B, and BMPRII primary antibodies (1:200 dilution) incubation, Alexa-conjugated anti-rabbit IgG (1:100) linking and 4′,6-diamidino-2-phenylindole (DAPI) (Beyotime Biotech) counterstaining were performed. The images were taken with a fluorescence microscope and merged.

### Counting LCs in EDS regeneration model in vivo

A fractionator technique was used to count CYP11A1^+^, HSD11B1^+^ LCs, or SOX9^+^ SCs. a digital camera with a 10× objective for the tissue-array sections to count cells of the “current” microscopic field. Then, the total number of LCs was calculated by multiplying LC number counted in a known fraction of the testis by the inverse of the sampling probability.

### RNA-sequencing, data analysis, and biological pathway analysis in vivo

Total RNAs were extracted from testes by Trizol solution (Invitrogen, Carlsbad, CA). After the RNAs were purified, using the NanoDrop 2000 to read RNA concentrations. Gene MicroArray Pathway Profiler 2.1 (GenMAPP2.1) software was used to find the biological pathway [39] and GO pathway was founded. The data was imported to GenMAPP2.1 to provide a map of signal pathways for the potential cascade. All the genes changed or not would be shown.

### Microarray re-analysis

Affymetrix DNA microarray of SLCs, PLCs, ILCs, ALCs, and BMSCs were published previously by our group as GSE26703 in the National Center for Biotechnology Information Gene Expression Omnibus [15]. Re-analyses of the data for Bmp family was performed and *Bmp3*, *Bmp4*, and *Bmp6* expression levels were presented.

### Quantitative real-time PCR (qPCR) in vivo and in vitro

Testis and PLCs were used for extract RNA using Trizol solution (Invitrogen, Carlsbad, CA). After RNAs were purified, the NanoDrop 2000 was used to read RNA concentrations. RNA samples were reverse transcribed into cDNAs. A SYBR Green qPCR Kit (Roche, Basel, Switzerland) was used to measure the LC mRNAs (*Lhcgr, Scarb1, Star, Cyp11a1, Hsd3b1, Srd5a1, Akr1c14*, and *Nr5a1*) and the BMP4 receptors mRNAs (*Bmpr1a, Bmpr1b, and Bmpr2*) as previously reported [38]. Melting curve analysis and gel electrophoresis was selected to identify the specificity. A standard curve using Ct values was generated to calculate the concentrations of target mRNA. The Bio-Rad CFX Manager Software was used to analyze the qPCR data. We used *Rps16* as the internal control. All the target mRNA levels were adjusted to *Rps16*.

### Western blot analysis

Western blot was to detect protein level. The testis or PLCs were placed into RIPA lysis buffer (Bocai Biotechnology, China) and homogenized. The protein concentrations of samples were measured using the BCA Protein Assay Kit (Takara, Japan). An aliquot (30 μg) of proteins was loaded and the proteins were electrophoresed on the 10 or 15% polyacrylamide gels containing sodium dodecyl sulfate and the proteins were transferred onto the nitrocellulose membrane. The membrane was blocked with 5% non-fat milk in TBST buffer for 2 h and incubated with primary antibodies at 4 °C overnight. The membrane was washed and incubated with HRP-conjugated anti-rabbit or anti-mouse (1:2000; Multi Sciences, Hangzhou, China) for 2 h at room temperature. ACTB was used as the internal control. All the target protein levels were adjusted to ACTB.

### Immunohistochemical semi-quantitative density detection

CYP11A1 and HSD11B1 are LC proteins and SOX9 is a SC protein. Immunohistochemical staining of these proteins in the tissue array were performed as previously described [38]. Briefly, the densities of CYP11A1, HSD11B1 or SOX9 and background area nearby were measured using the Image-Pro Plus 6.0 (Media Cybernetics, Silver Spring, MD) and mean density was set as measurement parameter. The net density of CYP11A1, HSD11B1, or SOX9 was calculated after subtracting the density of the background area. More than 50 LCs or SCs in each testis were counted and the density of each testis was averaged as one sample size.

### CO-IP in vitro

To assess the changes in protein-protein interaction between SMAD1/5 and its binding proteins (SMAD4) in PLCs following treatment with BMP4, we harvested the PLCs treated by BMP4 for 24 h, then Co-IP was performed as earlier described [40]. Firstly, the protein was extracted. After centrifugation, IgG was added to the supernatant and upside down at 4 ^o^C for 1 h. Then the protein A/G plus agarose (Santa Cruz Biotechnology) was added for 1 h and centrifuged for 5 min. The supernatant was taken and primary antibody was added and incubated at 4 ^o^C overnight. Protein A/G plus agarose was added and incubated at 4 ^o^C for 5–7 h. Nonidet P-40 was used to wash protein A/G plus agarose. Loading buffer was added, heated, boiled, centrifuged, and supernatant was collected. Western blot was performed to detect the interaction between protein and protein.

### Statistics

The experiment was repeated at least three times. All experimental data were statistically analyzed and mapped by GraphPad Prism 9 software (GraphPad Inc, CA.) and expressed as mean±standard error (SEM). The differences between the data of each group were analyzed and compared by unpaired Student’s *t* test or one-way ANOVA. The indicated *P* values (*P < 0.05, **P < 0.01, and ***P < 0.001) were considered statistically significant.

## Supplementary information

aj-checklist

Supplementary Materials-1

Supplemental Material-2

## Data Availability

The datasets used and analyzed during the current study are available from the corresponding author.
