# Supplementary material for: Bone morphogenetic protein 4 inhibits rat stem/progenitor Leydig cell development and regeneration via SMAD-dependent and SMAD-independent signaling
Source: Cell Death Dis. 2022 Dec 13;13(12):1039. doi: 10.1038/s41419-022-05471-8 (PMC9748027; doi:10.1038/s41419-022-05471-8)
Supplement: Supplementary file 2 — Supplementary Materials-1 [file 41419_2022_5471_MOESM2_ESM.docx]

**Bone morphogenetic protein 4 inhibits rat stem/progenitor Leydig cell development and regeneration via SMAD-dependent and SMAD-independent signaling**

Xiaoheng Li ^1^, Yinghui Fang ^1, 2^, Lanlan Chen ^3^, Hehua Quan ^1^, Yiyan Wang ^1^, Qiqi Zhu ^1^, Ren-Shan Ge ^1,^*

^1^ Department of Anesthesiology, The Second Affiliated Hospital and Yuying Children's Hospital of Wenzhou Medical University, 109 Xueyuan West Road, Wenzhou, Zhejiang 325027, China

^2^ Teaching and Research Office, Clinical Skills Experiment Center, Wenzhou Medical University, Wenzhou, Zhejiang 325035, China

^3^ Department of Anesthesiology, Taizhou People's Hospital, Fifth Affiliated Hospital of Nantong University, Taizhou City, Jiangsu, China

*Corresponding author: Ren-Shan Ge, MD

Department of Anesthesiology, The Second Affiliated Hospital and Yuying Children's Hospital of Wenzhou Medical University, Wenzhou, Zhejiang 325027, China; r_ge@yahoo.com

Running title: Effects of BMP4 on Leydig cell regeneration


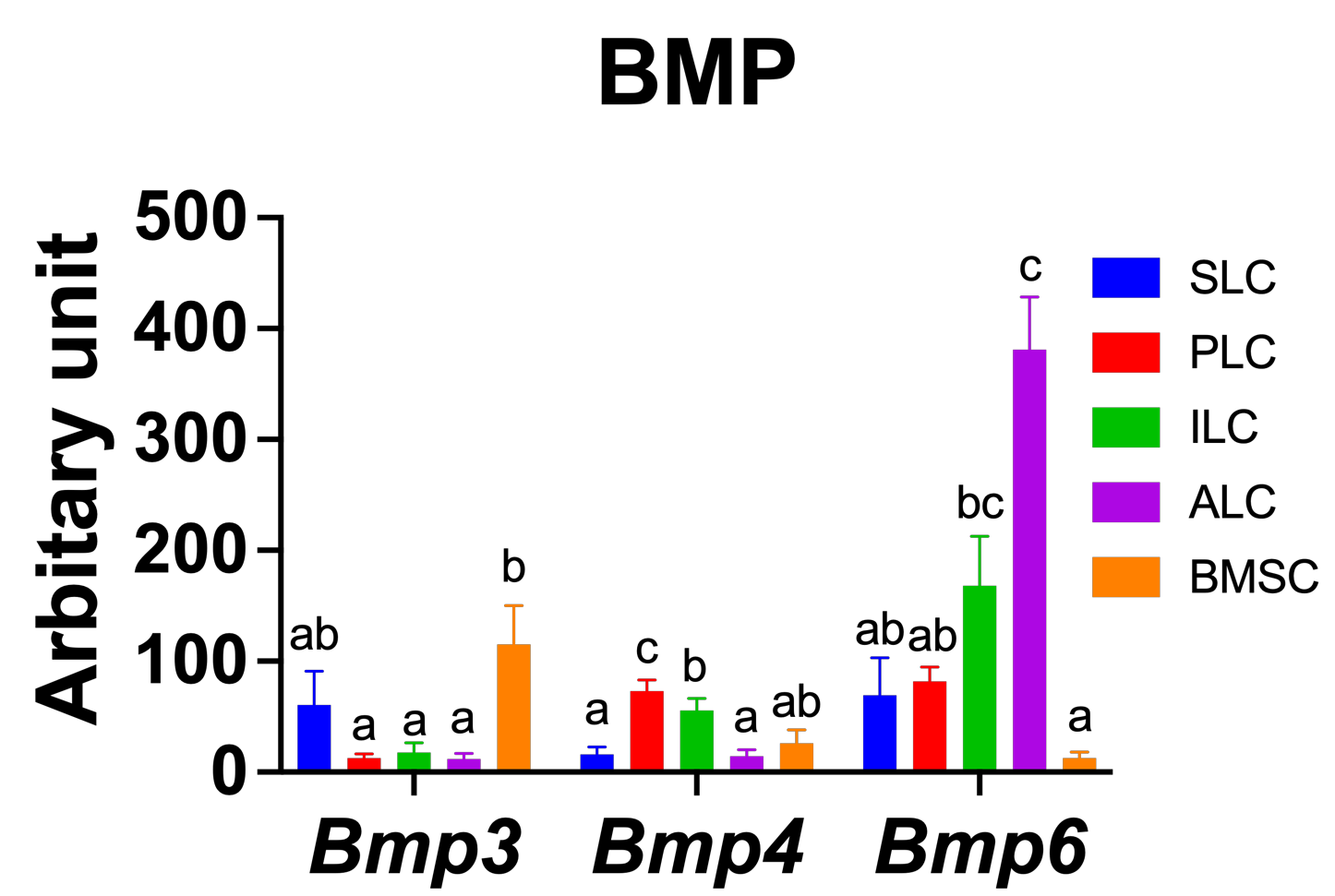


**Fig. S1** Microarray re-analysis showing gene expression of *Bmp3*, *Bmp4*, and *Bmp6* during Leydig cell development from stem Leydig cell (SLC) through progenitor Leydig cell (PLC), immature Leydig cell (ILC), and adult Leydig cell (ALC) stages and comparing them to bone marrow-derived mesenchymal stem cell (BMSC). *Bmp3* levels was about 60 units in SLC, were low in PLC, ILC and ALC and close to 100 units in BMSC. *Bmp4* levels in all cell types were well below 100 units. *Bmp6* levels were showed an increasing trend across the cell types-low in SLC and highest in ALC and very low levels in BMSC. The levels of *bmp3* and *4* are low but high in *bmp6*. Mean±SEM, n =4. Unidentical letter shows significant difference between two groups at P <0.05.


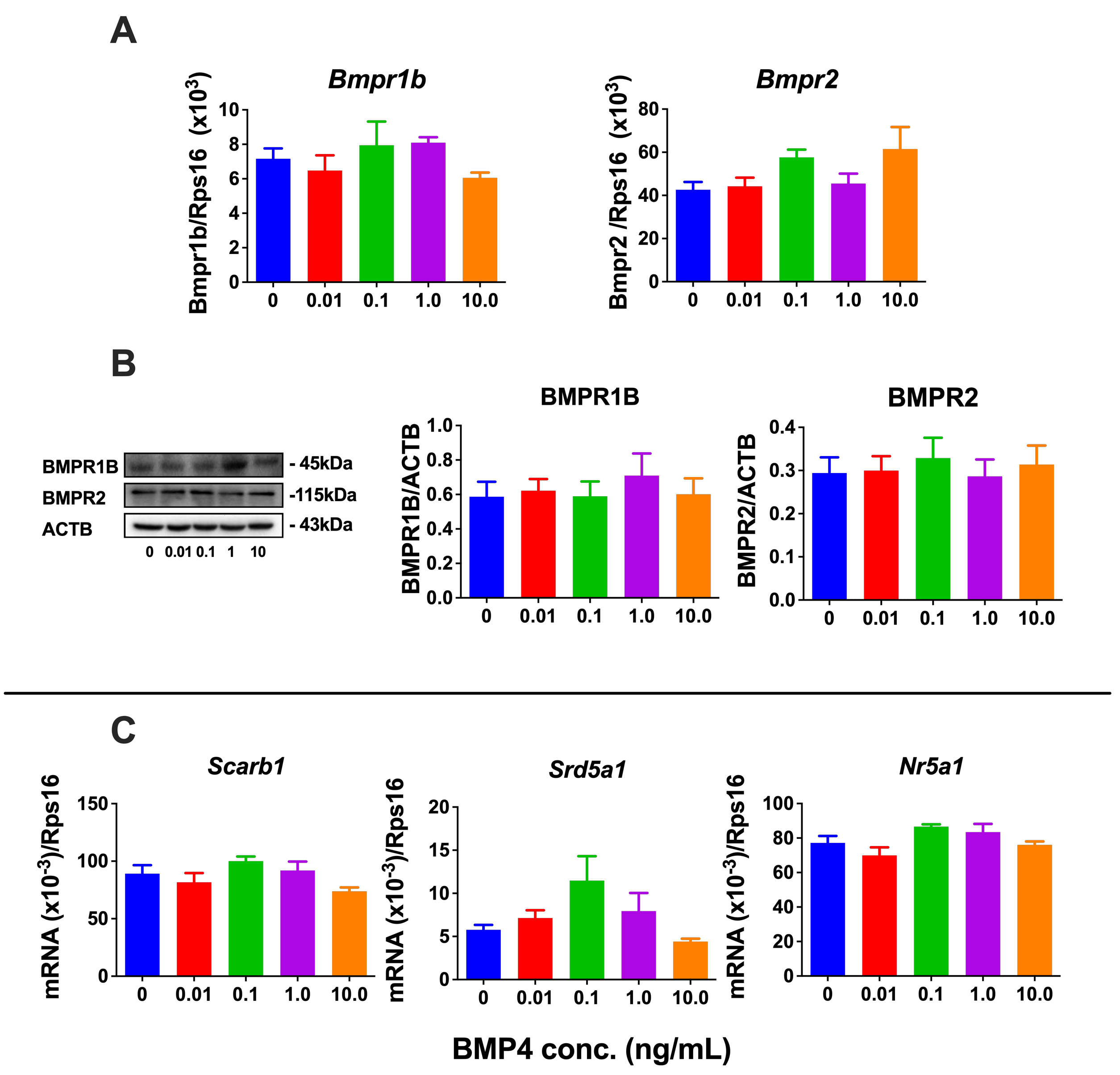


**Fig. S2** The effect of BMP4 on gene and protein expressions in progenitor Leydig cells (PLCs). PLCs were treated with BMP4 for 24 h. **(A, C)** Gene expression (*Bmpr1b* and *Bmpr2* as well as *Scarb1*, *Srd5a1*, *Nr5a1*) by qPCR, Mean±SEM, n=4; **(B)** BMPR1B and BMPR2 protein levels, Mean±SEM, n=4. No significant difference was observed between groups.


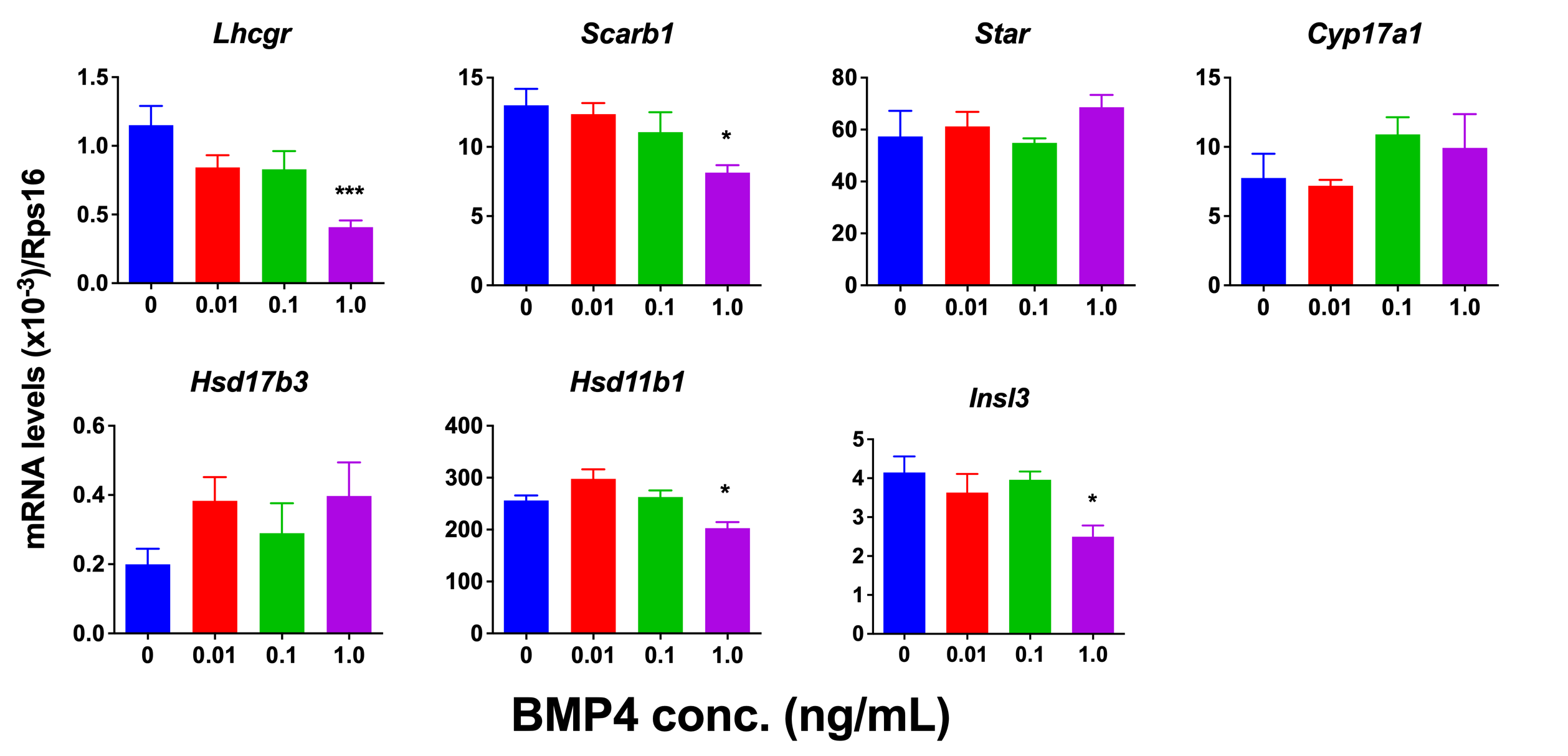


**Fig. S3** The effect of BMP4 on gene expressions in stem Leydig cells (SLCs) on seminiferous tubule (ST) surface. STs were cultured with BMP4 in basal medium for 1 week and then switched to Leydig cell differentiation medium for 2 weeks and gene expression was measured at the end of third week by qPCR. Mean±SEM, n=5-6; *P < 0.05, ***P < 0.001 indicate significant differences when compared to the control (0 ng/mL BMP4).


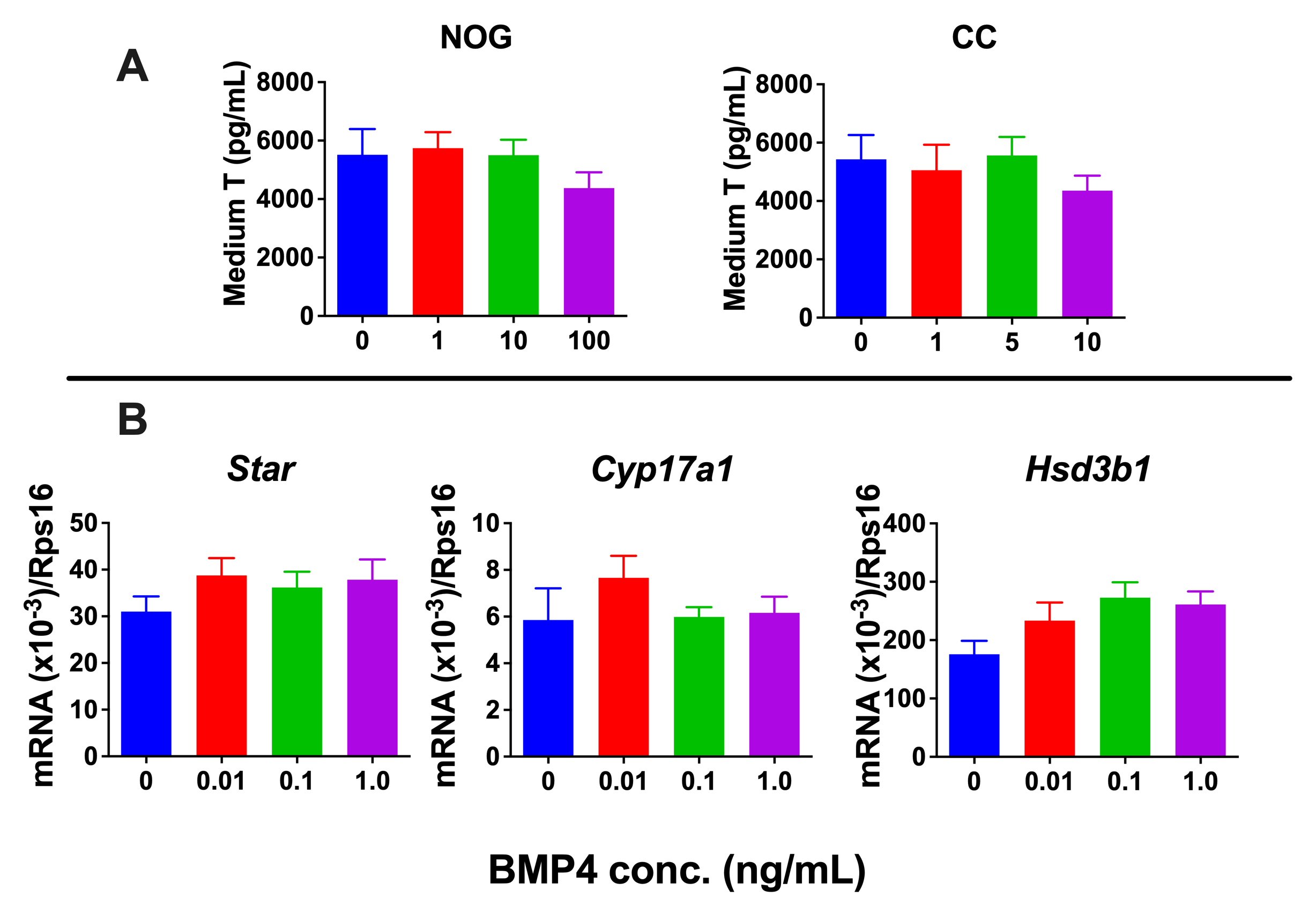


**Fig. S4** The effects of BMP4 on SLC differentiation *in vitro*. **(A)** The medium testosterone (T) levels after treatment of NOG or CC alone. Mean ± SE, n=6; **(B)** The mRNA levels of *Star*, *Cyp17a1*, and *Hsd3b1*, were analyzed by qPCR in the seminiferous tubules treated with BMP4 for 3 weeks. Mean±SEM, n=6. *Rps16* was used as internal reference. No significant difference was observed between two groups.

**Table S1.** General parameters of EDS-treated rats after intratesticular injection of BMP4 for 14 days

| Parameters |  | BMP4（ng/testis/day, intratesticular injection for 14 days） | | | |
| --- | --- | --- | --- | --- | --- |
|  |  | 0 | 0.1 | 1.0 | 10.0 |
| Rats number |  | 6 | 6 | 6 | 6 |
| Body weight (g) | before BMP4 | 313.50±8.33 | 329.60±11.29 | 334.50±9.81 | 316.50±10.18 |
|  | after BMP4 | 356.20±11.05 | 369.90±13.16 | 369.00±9.62 | 343.40±13.51 |
| Testis weight (g) |  | 2.28±0.26 | 2.52±0.14 | 2.33±0.27 | 2.35±0.33 |
| Epididymis weight (g) |  | 0.73±0.049 | 0.70±0.045 | 0.667±0.05 | 0.633±0.08 |

Mean±SEM, n =6. No significant difference was observed between two groups.


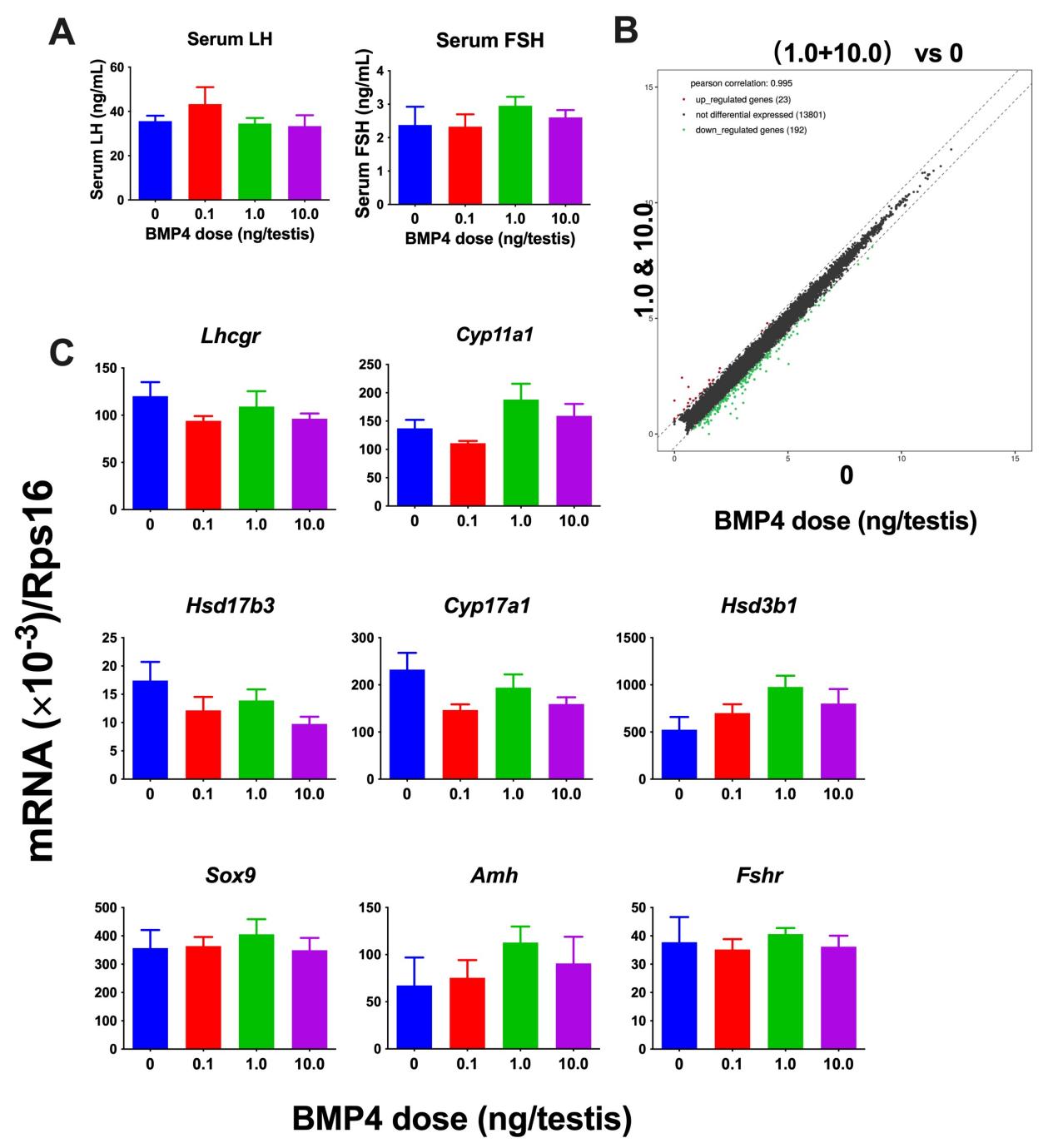


**Fig. S5** Serum LH, FSH levels and the expression levels of Leydig cell genes after BMP4 treatment for 14 days *in vivo.* **(A)** LH and FSH levels, Mean±SEM, n=6; **(B)** Scatter analysis of mRNAs between BMP4 (1.0 and 10.0 ng/testis) and control (0ng/testis) samples, Mean±SEM, n=3; **(C)** The mRNA levels of *Lhcgr*, *Cyp11a1*, *Hsd3b1*, *Hsd17b3*, *Cyp17a1*, *Sox9*, *Amh,* and *Fshr* were analyzed by qPCR in testes from the rats treated with 0, 0.1, 1.0, and 10.0ng/testis BMP4 on post-EDS day 14 for 14 days, Mean±SEM, n = 5 or 6. No significant difference was observed between two groups.


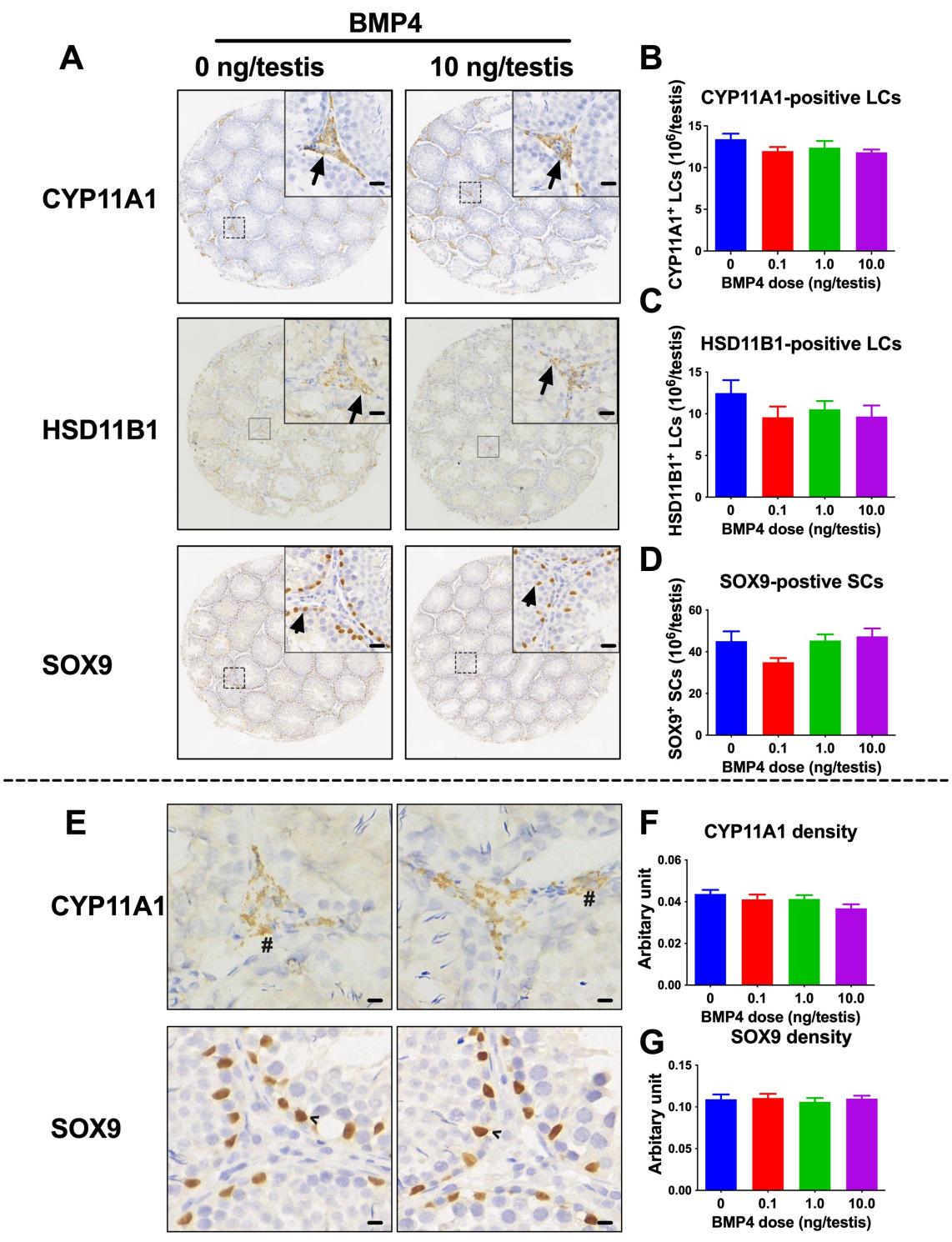


**Fig. S6** **(A-D)** Leydig cell (LC) and Sertoli cell (SC) numbers in the testes after BMP4 treatment *in vivo*. **(A)** Immunohistochemistry of CYP11A1, HSD11B1 and SOX9 of the testes from the rats treated with 0 and 10 ng/testis BMP4 on post-EDS day 14 for 14 days; **(B, C and D)** Quantitation. Arrow, CYP11A1^+^ and HSD11B1^+^ LCs; arrowhead, SOX9^+^ SCs. **(E-G)** Semi-quantitation of CYP11A1 and SOX9 density. **#,** CYP11A1^+^ LCs; **^**, SOX9^+^ SCs. Mean±SEM, n=6. Bar = 50 μm. No significant difference was observed between two groups.

**Table S2.** Genes were down-regulated after BMP4 treatment

| Gene Symbol | Gene Name | BMP4（ng/testis） | | |
| --- | --- | --- | --- | --- |
|  |  | 0(A) | 1(C) | 10(D) |
| **Cell structure, morphology and movement** | | | | |
| Rflnb | Refilin B | 1.74 | 0.44 (3.95) | 0.44 (3.98) |
| Synpo | Synaptopodin | 0.92 | 0.48 (1.94) | 0.27 (3.44) |
| Cd200 | CD200 Molecule | 3.03 | 1.29 (2.34) | 1.04 (2.91) |
| Sdc3 | Syndecan 3 | 1.22 | 0.63 (1.95) | 0.45 (2.71) |
| Cdh17 | Cadherin 17 | 3.94 | 2.12 (1.86) | 1.53 (2.57) |
| **Cytokines, growth factors and receptors** | | | | |
| Hamp | Hepcidin Antimicrobial Peptide | 5.73 | 1.72 (3.33) | 0.78 (7.35) |
| Scube2 | Signal Peptide, CUB Domain And EGF Like Domain Containing 2 | 0.96 | 0.22 (4.48) | 0.20 (4.73) |
| Scn3b | Sodium Voltage-Gated Channel  Beta Subunit 3 | 1.60 | 0.56 (2.87) | 0.36 (4.50) |
| Tnfrsf12a | TNF Receptor Superfamily Member 12A | 7.55 | 2.33 (3.24) | 1.66 (4.56) |
| S100a11 | S100 Calcium Binding Protein A11 | 15.99 | 7.48 (2.14) | 3.62 (4.42) |
| Testin | Testin LIM Domain Protein | 33.57 | 10.08 (3.33) | 7.80 (4.30) |
| Cx3cl1 | C-X3-C Motif Chemokine Ligand 1 | 2.91 | 1.13 (2.57) | 0.68 (4.29) |
| Nat8l | N-Acetyltransferase 8 Like | 1.90 | 0.73 (2.62) | 0.44 (4.29) |
| Il17re | Interleukin 17 Receptor A | 2.55 | 1.04 (2.46) | 0.60 (4.23) |
| Bgn | Biglycan | 24.41 | 10.69 (2.28) | 6.06 (4.03) |
| Il9 | Interleukin 9 | 2.48 | 1.91 (1.30) | 0.66 (3.77) |
| Gpnmb | Glycoprotein Nmb | 2.10 | 1.30 (1.62) | 0.56 (3.75) |
| Serpinb1a | Serpin Family B Member 1 | 8.23 | 3.40 (2.42) | 2.31 (3.57) |
| Gprc5a | G Protein-Coupled Receptor  Class C Group 5 Member A | 3.31 | 1.67 (1.99) | 0.94 (3.54) |
| Dmp1 | Dentin Matrix Acidic Phosphoprotein 1 | 1.19 | 0.49 (2.43) | 0.35 (3.40) |
| Il1r1 | Interleukin 1 Receptor Type 1 | 2.81 | 1.15 (2.44) | 0.83 (3.39) |
| Prrt1 | Proline Rich Transmembrane Protein 1 | 1.95 | 0.80 (2.43) | 0.58 (3.36) |
| Gal | Galanin And GMAP Prepropeptide | 1.63 | 0.81 (2.01) | 0.49 (3.34) |
| Sstr3 | Somatostatin Receptor 3 | 1.60 | 0.65 (2.45) | 0.48 (3.31) |
| Myof | Myoferlin | 2.88 | 1.05 (2.73) | 0.87 (3.30) |
| Prrg4 | Proline Rich And Gla Domain 4 | 2.03 | 1.18 (1.72) | 0.63 (3.20) |
| Scara3 | Scavenger Receptor Class A Member 3 | 2.01 | 0.79 (2.54) | 0.63 (3.19) |
| Slc22a25 | Solute Carrier Family 22 Member 25 | 1.27 | 0.85 (1.50) | 0.43 (2.98) |
| Tspan4 | Tetraspanin 4 | 1.80 | 0.92 (1.97) | 0.61 (2.94) |
| Fgf18 | Fibroblast Growth Factor 18 | 8.06 | 6.63 (1.21) | 2.76 (2.92) |
| Myc | MYC Proto-Oncogene, BHLH  Transcription Factor | 1.05 | 0.60 (1.75) | 0.36 (2.90) |
| Prl6a1 | Prolactin | 1.28 | 0.76 (1.69) | 0.45 (2.82) |
| Pdyn | Prodynorphin | 5.50 | 2.49 (2.21) | 1.96 (2.81) |
| Nfil3 | Nuclear Factor, Interleukin 3 Regulated | 1.39 | 0.70 (1.99) | 0.49 (2.81) |
| Lcn2 | Lipocalin 2 | 55.75 | 34.22 (1.63) | 19.94 (2.80) |
| Pla2g2a | Phospholipase A2 Group IIA | 1.38 | 1.06 (1.31) | 0.50 (2.79) |
| Pon2 | Paraoxonase 2 | 3.99 | 2.02 (1.97) | 1.47 (2.71) |
| Defb11 | Defensin Beta 110 | 8.89 | 4.80 (1.85) | 3.50 (2.54) |
| Mnda | Myeloid Cell Nuclear Differentiation Antigen | 1.93 | 0.89 (2.17) | 0.76 (2.53) |
| Timp1 | TIMP Metallopeptidase Inhibitor 1 | 42.72 | 21.40 (2.00) | 17.01 (2.51) |
| Shisa5 | Shisa Family Member 5 | 2.26 | 1.54 (1.46) | 0.90 (2.50) |
| **Intracellular Signing** | | | | |
| Pla2g5 | Phospholipase A2 Group V | 1.84 | 0.55 (3.37) | 0.40 (4.64) |
| Wnt4 | Wnt Family Member 4 | 6.49 | 1.93 (3.36) | 1.29 (5.03) |
| Fos | Fos Proto-Oncogene,  AP-1 Transcription Factor Subunit | 1.42 | 0.33 (4.35) | 0.41 (3.47) |
| Cd276 | CD276 Molecule | 1.67 | 0.90 (1.85) | 0.57 (2.96) |
| Gbp2 | Guanylate Binding Protein 2 | 10.69 | 5.23 (2.04) | 3.61 (2.96) |
| C1qtnf1 | C1q And TNF Related 1 | 2.23 | 1.12 (1.99) | 0.76 (2.93) |
| Ubd | Ubiquitin D | 1.27 | 0.72 (1.76) | 0.43 (2.92) |
| Pdgfa | Platelet Derived Growth Factor Subunit A | 2.82 | 1.33 (2.13) | 0.97 (2.92) |
| Gbp5 | Guanylate Binding Protein 5 | 1.72 | 1.03 (1.68) | 0.63 (2.72) |
| **Synthesis and Metabolism** | |  |  |  |
| Fah | Fumarylacetoacetate Hydrolase | 1.21 | 0.68 (1.78) | 0.26 (4.60) |
| Slc35d2 | Solute Carrier Family 35 Member D2 | 1.19 | 0.55 (2.17) | 0.29 (4.06) |
| Trim2 | Tripartite Motif Containing 2 | 1.14 | 0.34 (3.40) | 0.29 (3.92) |
| Nefh | Neurofilament Heavy | 2.73 | 0.91 (3.00) | 0.60 (4.56) |
| Dhtkd1 | Dehydrogenase E1  And Transketolase Domain Containing 1 | 1.87 | 1.83 (1.02) | 0.53 (3.53) |
| Tph1 | Tryptophan Hydroxylase 1 | 1.62 | 0.88 (1.84) | 0.48 (3.36) |
| Akr1cl | Aldo-Keto Reductase Family 1 Member C2 | 0.90 | 0.23 (3.92) | 0.31 (2.91) |
| Pam | Peptidylglycine Alpha-Amidating  Monooxygenase | 13.14 | 4.79 (2.74) | 3.84 (3.43) |
| Ust | Uronyl 2-Sulfotransferase | 1.16 | 0.63 (1.83) | 0.40 (2.90) |
| Fmo5 | Flavin Containing Monooxygenase 5 | 1.03 | 0.36 (2.85) | 0.36 (2.85) |
| Pah | Phenylalanine Hydroxylase | 5.32 | 2.08 (2.56) | 1.89 (2.82) |
| Hist2h3c2 | Histone Cluster 1 H3 Family Member G | 1.13 | 1.16 (0.97) | 0.40 (2.80) |
| Nat1 | N-Acetyltransferase 1 | 2.96 | 1.62 (1.83) | 1.06 (2.79) |
| Pld1 | Phospholipase D1 | 1.06 | 0.58 (1.83) | 0.42 (2.54) |
| C4b | Complement C4B (Chido Blood Group) | 2.43 | 1.85 (1.32) | 0.97 (2.52) |
| **Unknown** | | | | |
| AABR07071891.1 |  | 1.23 | 0.45 (2.73) | 0.24 (5.11) |
| Lrrn4 | Leucine Rich Repeat Neuronal 4 | 3.76 | 1.44 (2.60) | 1.00 (3.78) |
| Foxs1 | Forkhead Box S1 | 1.09 | 0.69 (1.58) | 0.41 (2.65) |

Note: () fold changes when compared to control. The table lists the gene symbol with down regulation multiple≥2.5 in group D compared to group A.

**Table S3.** Genes were upregulated after BMP4 treatment

| Gene Symbol | Gene Name | BMP4（ng/testis） | | |
| --- | --- | --- | --- | --- |
|  |  | 0(A) | 1(C) | 10(D) |
| **Intracellular Signing** | | | | |
| Skor1 | SKI Family Transcriptional Corepressor 1 | 2.11 | 3.4975（1.66） | 4.2475（4.25） |
| Nmral1 | NmrA Like Redox Sensor 1 | 0.97 | 1.9375（2.00） | 2.075（2.08） |

Note: () fold changes when compared to control. The table lists the gene symbol with up regulation multiple≥2.0 in group D compared to group A.

### Table S4. Primer information

| Primer  Symbol | Gene name | Primer direction | Sequences (5’to 3’) |
| --- | --- | --- | --- |
| *Bmpr1a* | Bone Morphogenetic Protein Receptor Type 1A | Forward  Reverse | GCTGTGCTCATCTCTATGGCTGTC  TGCTTCATCCTGTTCCAAGTCACG |
| *Bmpr1b* | Bone Morphogenetic Protein Receptor Type 1B | Forward  Reverse | ACGGTCCTGATGAGGCACGAG  AGGCCGCTGACAGACGAGTAG |
| *Bmpr2* | Bone Morphogenetic Protein Receptor Type 2 | Forward  Reverse | CCACTCAGTCCGCCTCATTCATC  GCCGCCTCCATCATGCTCAC |
| *Lhcgr* | Luteinizing hormone receptor | Forward | CTGCGCTGTCCTGGCC |
|  |  | Reverse | CGACCTCATTAAGTCCCCTGAA |
| *Scarb1* | high-density lipoprotein receptor | Forward | ATGGTACTGCCGGGCAGAT |
|  |  | Reverse | CGAACACCCTTGATTCCTGGTA |
| *Star* | Steroidogenic acute regulatory protein | Forward | CATCCAGCAAGGAGAGGAAG |
|  |  | Reverse | CACCTGGCACCACCTTACTT |
| *Cyp11a1* | Cholesterol side ch-ain cleavage enzyme | Forward | AAGTATCCGTGATGTGGGG |
|  |  | Reverse | TCATACAGTGTCGCCTTTTCT |
| *Hsd3b1* | 3β-Hydroxysteroid dehydrogenase 1 | Forward | CCCTGCTCTACTGGCTTGC |
|  |  | Reverse | TCTGCTTGGCTTCCTCCC |
| *Hsd17b3* | 17β-Hydroxysteroid dehydrogenase 3 | Forward | AACAGTTCCTCCTTTCCGTG |
|  |  | Reverse | AATGAATAGGCTTTCCCGAT |
| *Hsd11b1* | Hydroxysteroid 11β-dehydrogenase 1 | Forward | GAAGAAGCATGGAGGTCAAC |
|  |  | Reverse | GCAATCAGAGGTTGGGTCAT |
| *Insl3* | Insulin Like 3 | Forward  Reverse | GTGGCTGGAGCAACGACA  AGAAGCCTGGTGAGGAAGC |
| *Cyp17a1* | P450 17α-hydroxylase/ 17,20-lyase | Forward | TGGCTTTCCTGGTGCACAATC |
|  |  | Reverse | TGAAAGTTGGTGTTCGGCTGAAG |
| *Srd5a1* | Steroid 5-reductase 1 | Forward | CAATCCTGCAAGATTCCACC |
|  |  | Reverse | ATTGGTCCTTGGGTGCATTC |
| *Akr1c14* | 3-Hydroxysteroid dehydrogenase | Forward | GCAGCGTGGGGTTGTG |
|  |  | Reverse | TGGATGATTGGGATGGTCA |
| *Nr5a1* | Nuclear receptor steroidogenic factor 1 | Forward | CAGAGCTGCAAAATCGACAA |
|  |  | Reverse | CCCGAATCTGTGCTTTCTTC |
| *Fshr* | Follicle stimulating hormone receptor | Forward | CCACAAGCCAATACAAACTAACT |
|  |  | Reverse | CAAAAGTCCAGCCCAATACC |
| *Amh* | Anti-Mullerian Hormone | Forward | GCCCTAACCCTTCAACCA |
|  |  | Reverse | GGGAATCAGAGCCAAACAGA |
| *Sox9* | SRY box 9 | Forward | TGCTGAACGAGAGCGAGAAG |
|  |  | Reverse | ATGTGAGTCTGTTCGGTGGC |
| *Rps16* | Ribosomal  protein s16 | Forward  Reverse | AAGTCTTCGGACGCAAGAAA  TTGCCCAGAAGCAGAACAG |

### Table S5. Antibody information

| Antibody | **Species** | **Vendor (City, State)** | **Dilution** | | |
| --- | --- | --- | --- | --- | --- |
|  |  |  | WB | HS | IF |
| Actin (ACTB) | Mouse | Beyotime (Shanghai, China), AF0003 | 1:1000 | ND | ND |
| LHCGR | Rabbit | Multi Sciences (Hangzhou, China), ab7496 | 1:1000 | ND | ND |
| STAR | Rabbit | SANTA CRUZ (Dallas,TX), sc-166821 | 1:1000 | ND | ND |
| HSD3B1 | Mouse | Novus Biologicals(Littleton, CO),NB110-78644 | 1:1000 | 1:1000 | ND |
| CYP11A1 | Rabbit | Cell Signaling Technology (Danvers, MA), 14217S | 1:1000 | 1:200 | ND |
| HSD11B1 | Rabbit | Abcam (San Francisco, CA), ab169785 | 1:1000 | 1:200 | ND |
| SCARB1 | Rabbit | Multi Sciences (Hangzhou, China), ab1967 | 1:1000 | ND | ND |
| INSL3 | Rabbit | Abcam (San Francisco, CA), ab65981 | 1:1000 | ND | ND |
| HSD17B3 | Rabbit | Cloud-Clone (Wuhan, China), PAF173Hu01 | 1:500 | ND | ND |
| BMPR1A | Rabbit | Abcam (San Francisco, CA), ab38560 | 1:1000 | 1:100 | 1:200 |
| BMPR1B | Mouse | SANTA CRUZ (Dallas,TX), sc-515886 | 1:1000 | 1:100 | 1:200 |
| BMPR2 | Mouse | SANTA CRUZ (Dallas,TX), sc-393304 | 1:1000 | 1:100 | 1:200 |
| SMAD1/5 | Mouse | Abcam (San Francisco, CA), ab75273 | 1:1000 | ND | ND |
| pSMAD1/5 | Rabbit | Cell Signaling Technology (Danvers, MA), 9516S | 1:1000 | ND | ND |
| SMAD4 | Rabbit | Cell Signaling Technology (Danvers, MA), 45535S | 1:1000 | ND | ND |
| SOX9 | Rabbit | Abcam (San Francisco, CA), ab185230 | 1:1000 | 1:1000 | ND |
| AMPK | Rabbit | Cell Signaling Technology (Danvers, MA), 5832S | 1:1000 | ND | ND |
| pAMPK | Rabbit | Cell Signaling Technology (Danvers, MA), 2535L | 1:1000 | ND | ND |
| ERK1/2 | Mouse | Abcam (San Francisco, CA),ab54230 | 1:1000 | ND | ND |
| pERK1/2 | Mouse | Abcam (San Francisco, CA), ab50211 | 1:5000 | ND | ND |
| SIRT1 | Mouse | Abcam (San Francisco, CA), 8469S | 1:2000 | ND | ND |
| PGC-1α | Rabbit | Abcam (San Francisco, CA), ab72230 | 1:5000 | ND | ND |
| BCL-2 | Rabbit | Cell Signaling Technology (Danvers, MA), 2870S | 1:1000 | ND | ND |
| BAX | Rabbit | Absin (Shanghai, China), abs130057 | 1:1000 | ND | ND |
| Goat anti-mouse IgG(H+L) HRP conjugated | Mouse | Multi Sciences (Hangzhou, China), GAM0072 | 1:2000 | ND | ND |
| Goat anti-Rabbit IgG(H+L) HRP conjugated | Rabbit | Multi Sciences (Hangzhou, China), GAR0072 | 1:2000 | ND | ND |

WB = Western blot; HS = Histochemical staining; IF=Immunofluorescence; ND = Not detected.


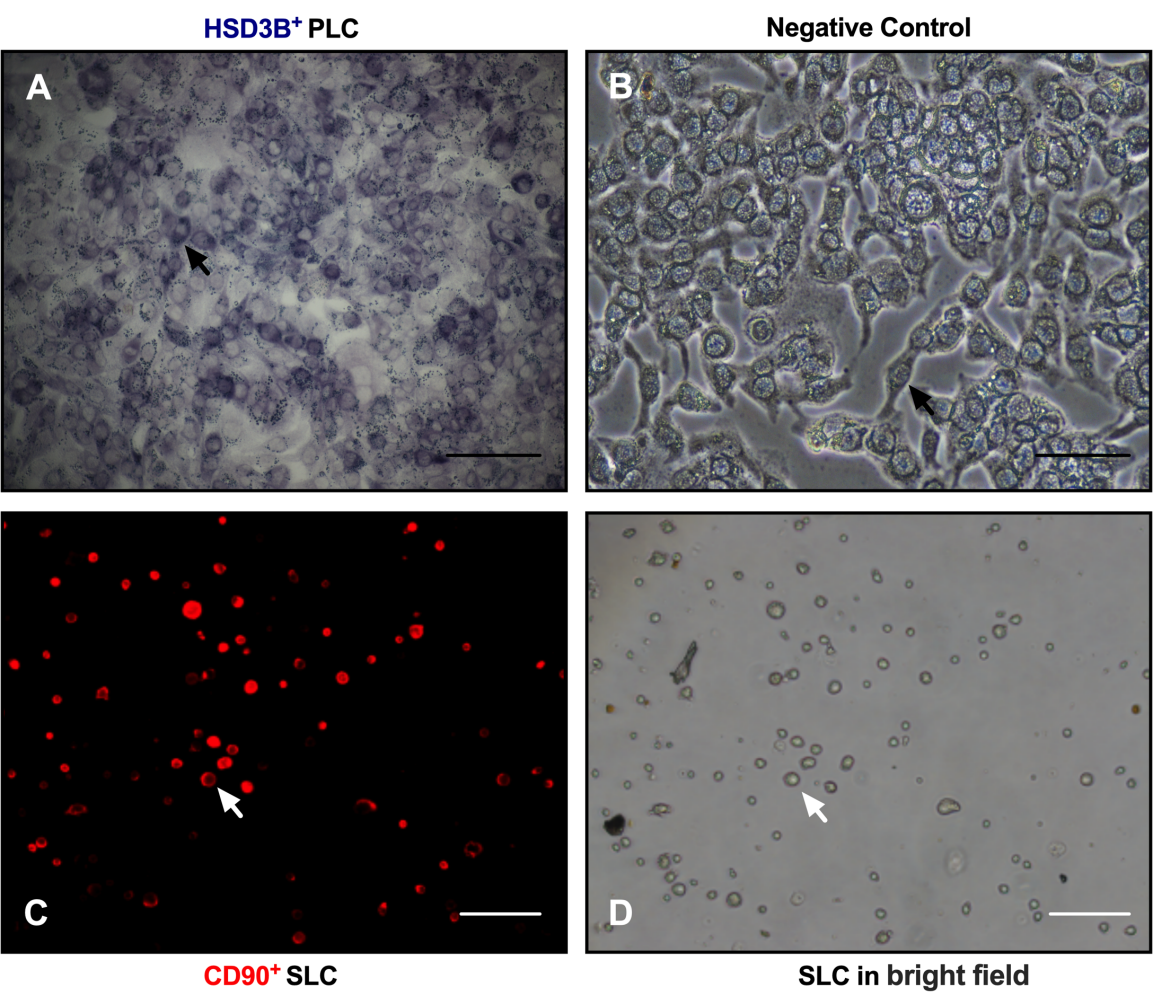


**Fig. S7** Characterization of progenitor Leydig cells (PLCs) and stem Leydig cells (SLCs). **(A)** HSD3B1 enzymatic staining of PLCs *in vitro*; Black arrow points to HSD3B1^+^PLC. **(B)** Negative control; Black arrows point to PLC (unstained). **(C)** CD90 staining of SLCs *in vitro*; White arrow points to CD90^+^SLC (red). **(D)** CD90^+^ SLCs and dead cells in the bright field; White arrow points to CD90^+^ SLC (bright).


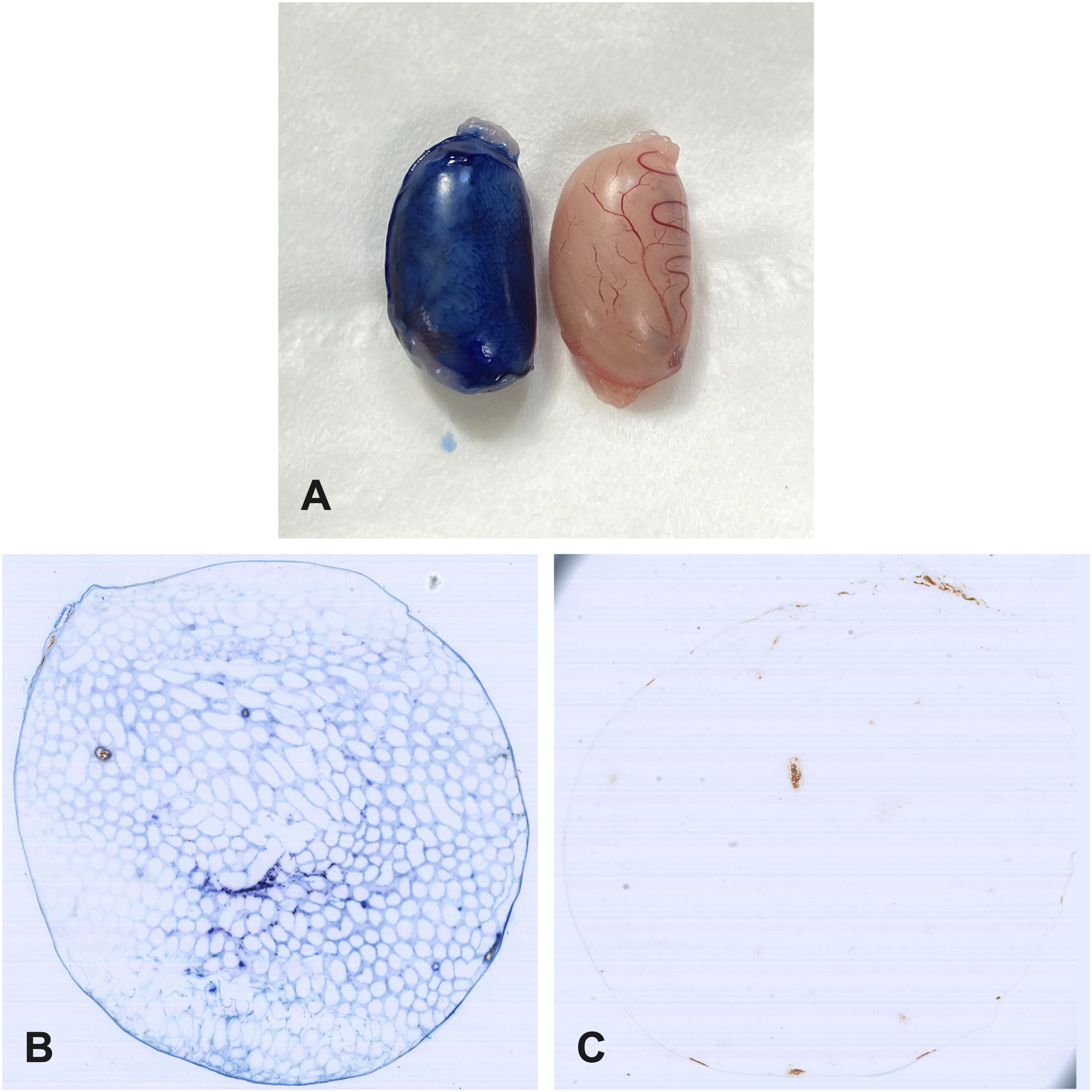


**Fig. S8** Verification of intratesticular injection using Trypan blue. **(A)** Testis with Trypan blue injection showing that whole testis was stained (left side), and the contralateral testis without Trypan blue (right side); **(B)** Frozen section of testis with Trypan blue, showing that Trypan blue was present in interstitium not within the seminiferous cords; **(C)** Negative control (saline injection).
