# Supplementary material for: Bone morphogenetic protein 4 inhibits rat stem/progenitor Leydig cell development and regeneration via SMAD-dependent and SMAD-independent signaling
Source: Cell Death Dis. 2022 Dec 13;13(12):1039. doi: 10.1038/s41419-022-05471-8 (PMC9748027; doi:10.1038/s41419-022-05471-8)

ACTB-FIG2:
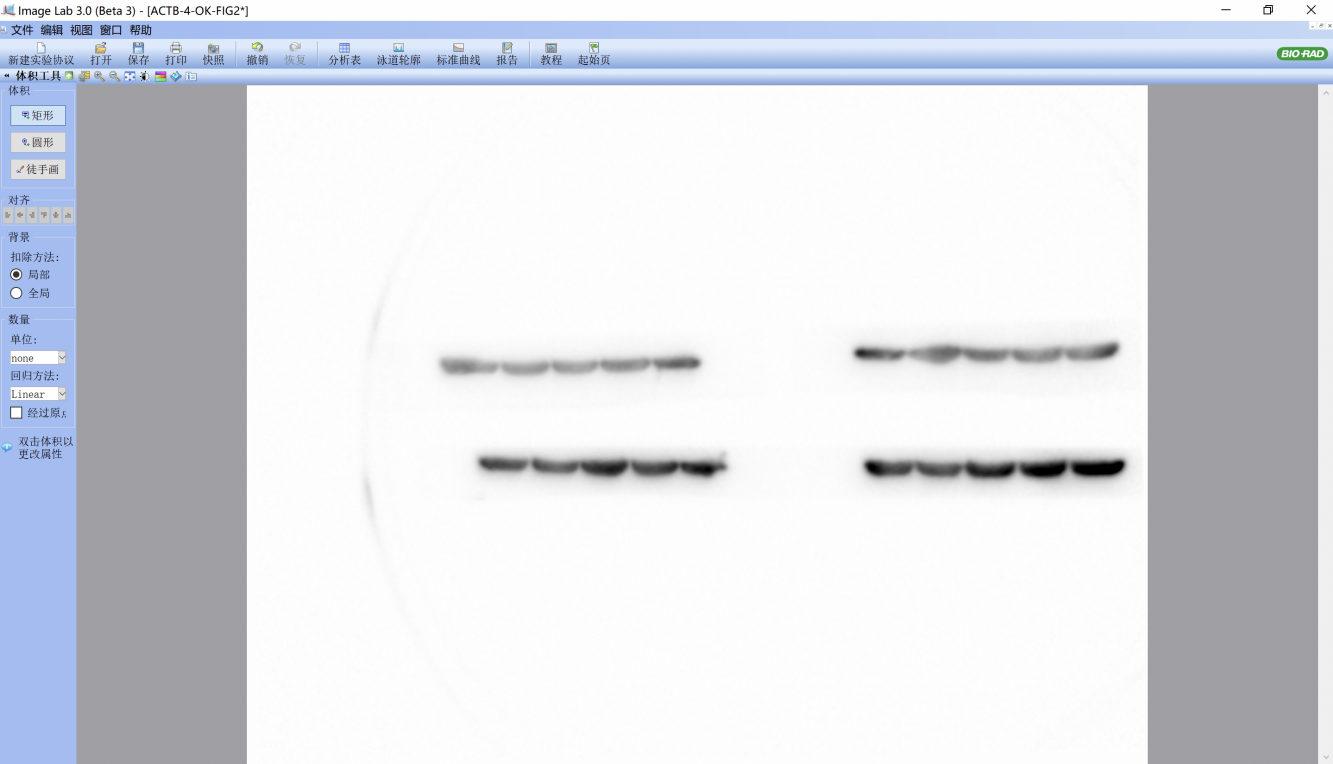


AKR1C14-FIG2:
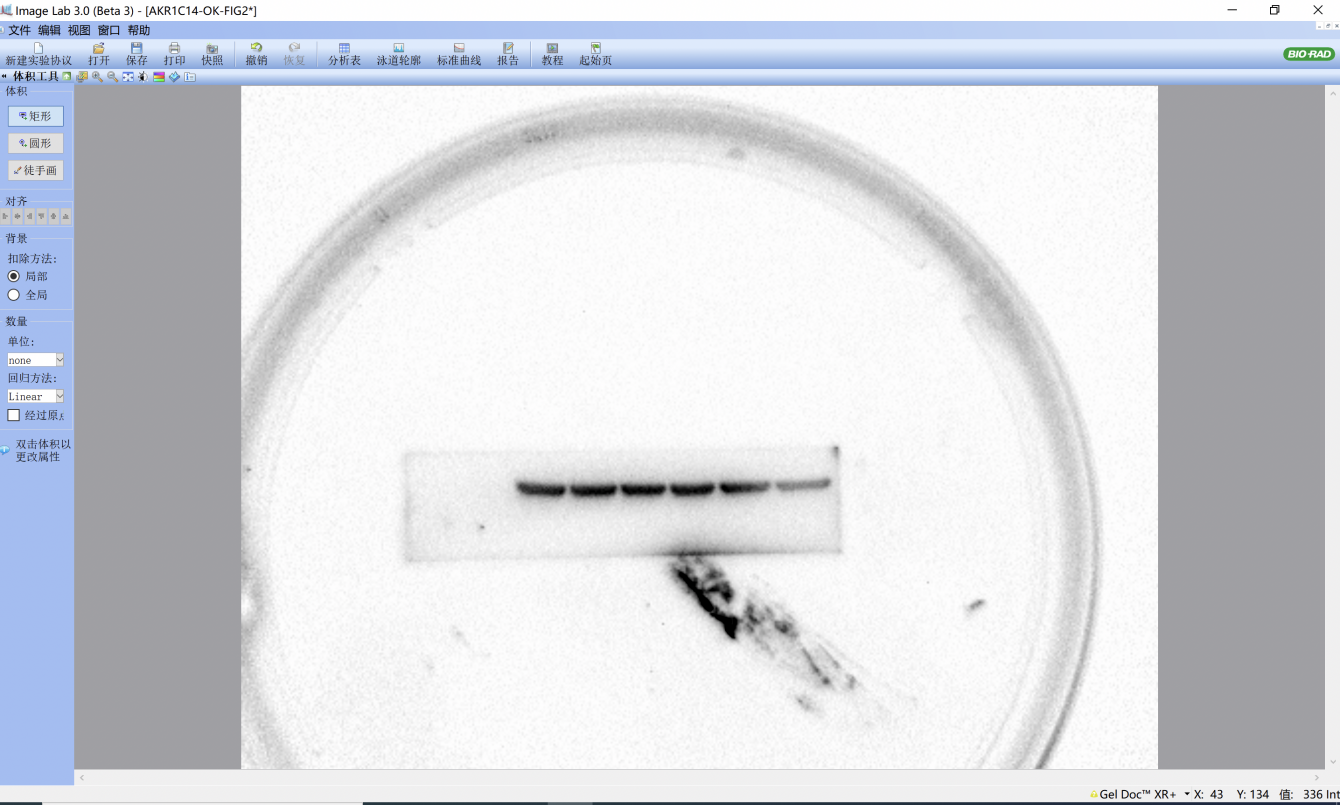


BMPR1A-FIG2:
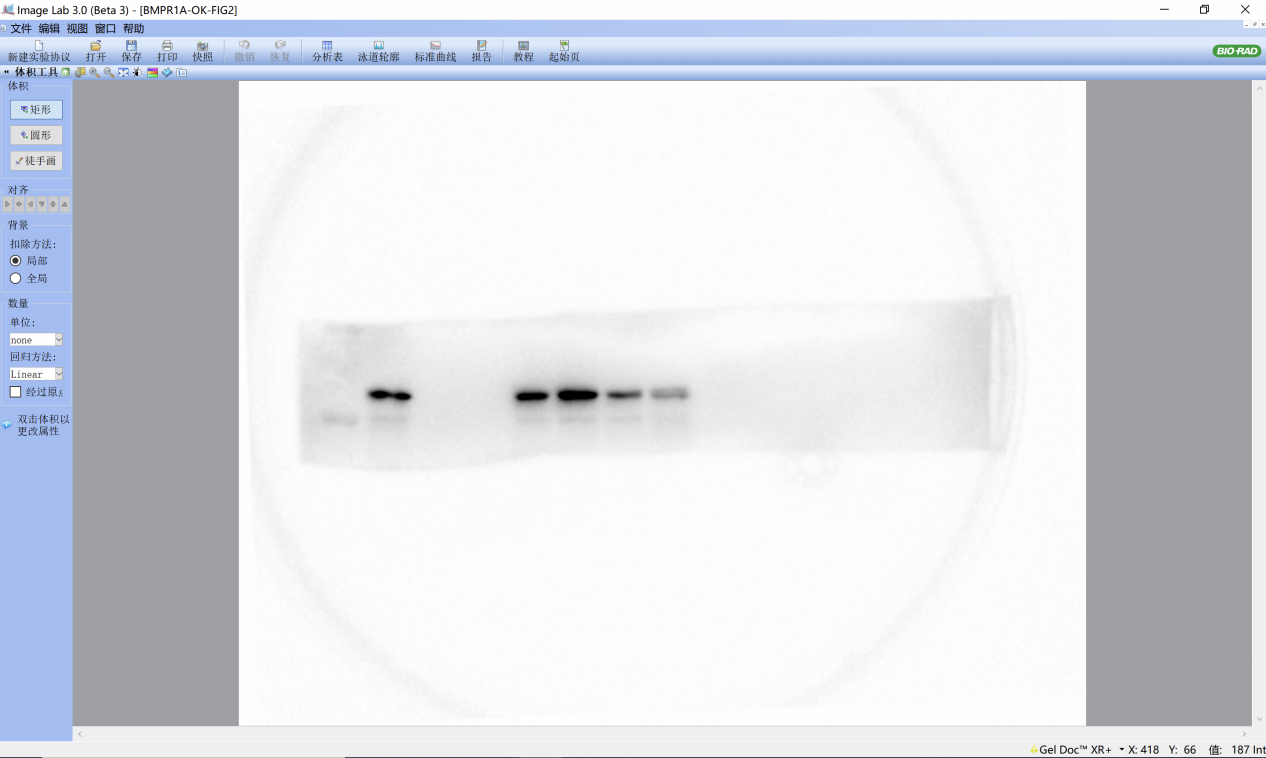


CYP11A1-FIG2:


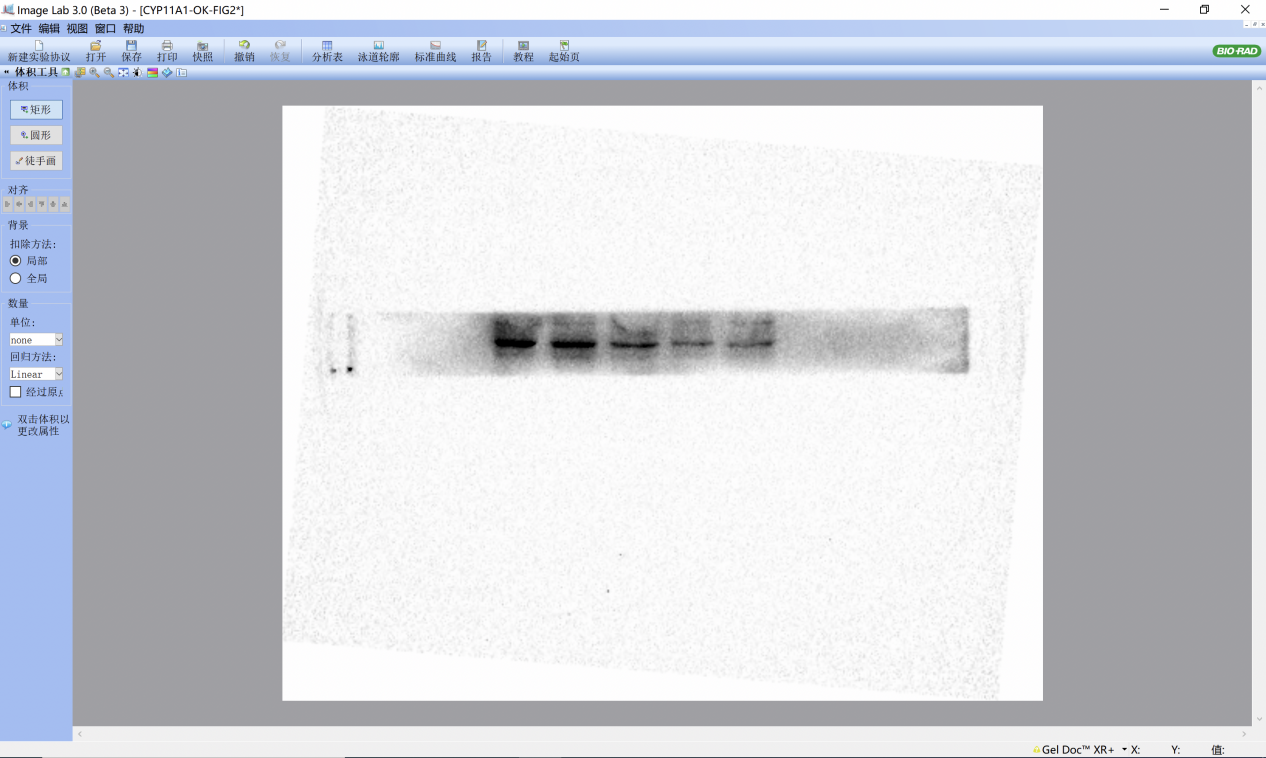


STAR-FIG2:
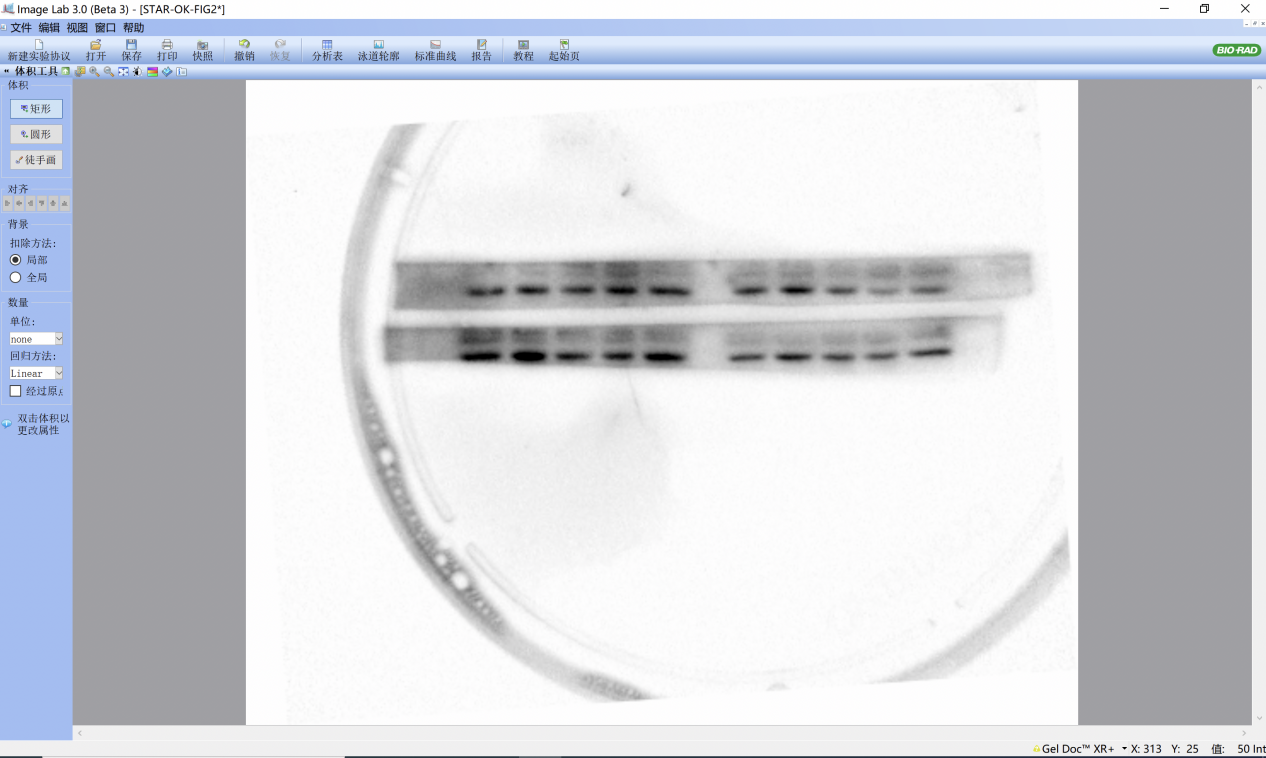


LHCGR-FIG2:
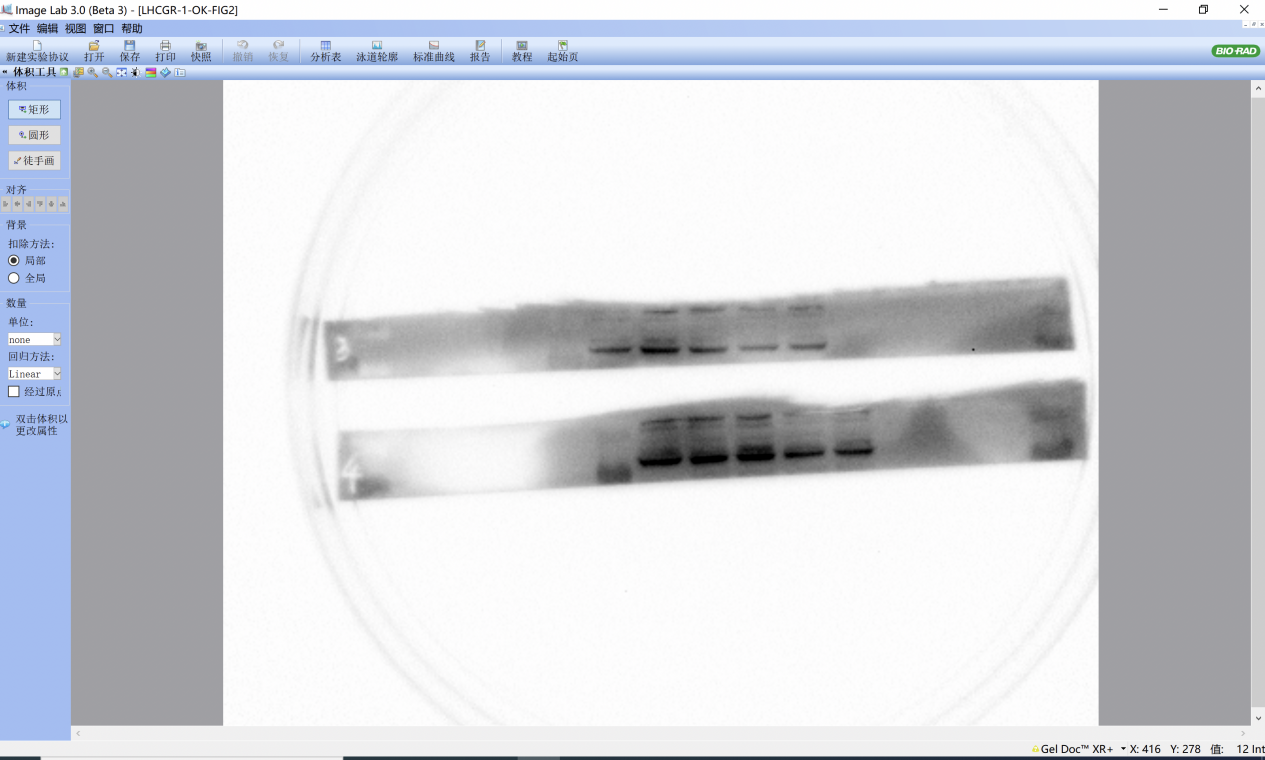


HSD3B1-FIG2:
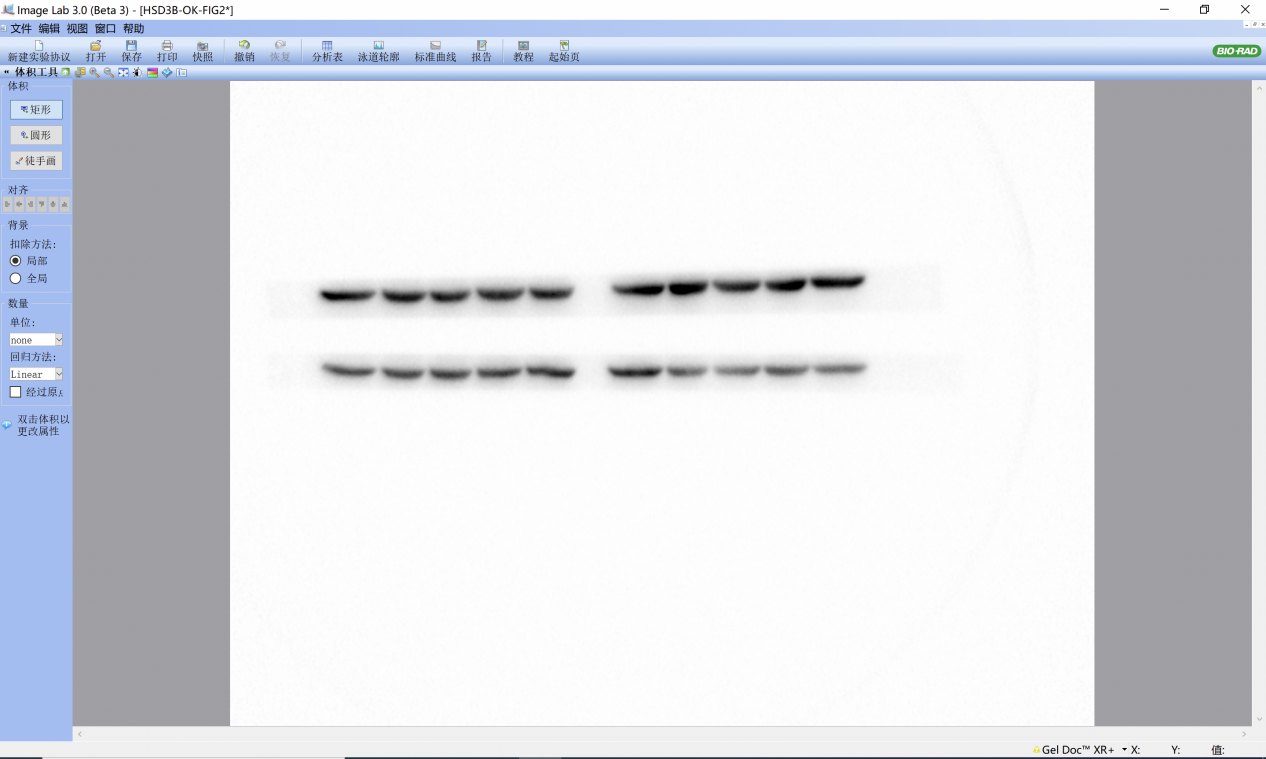


ACTB-FIG3:
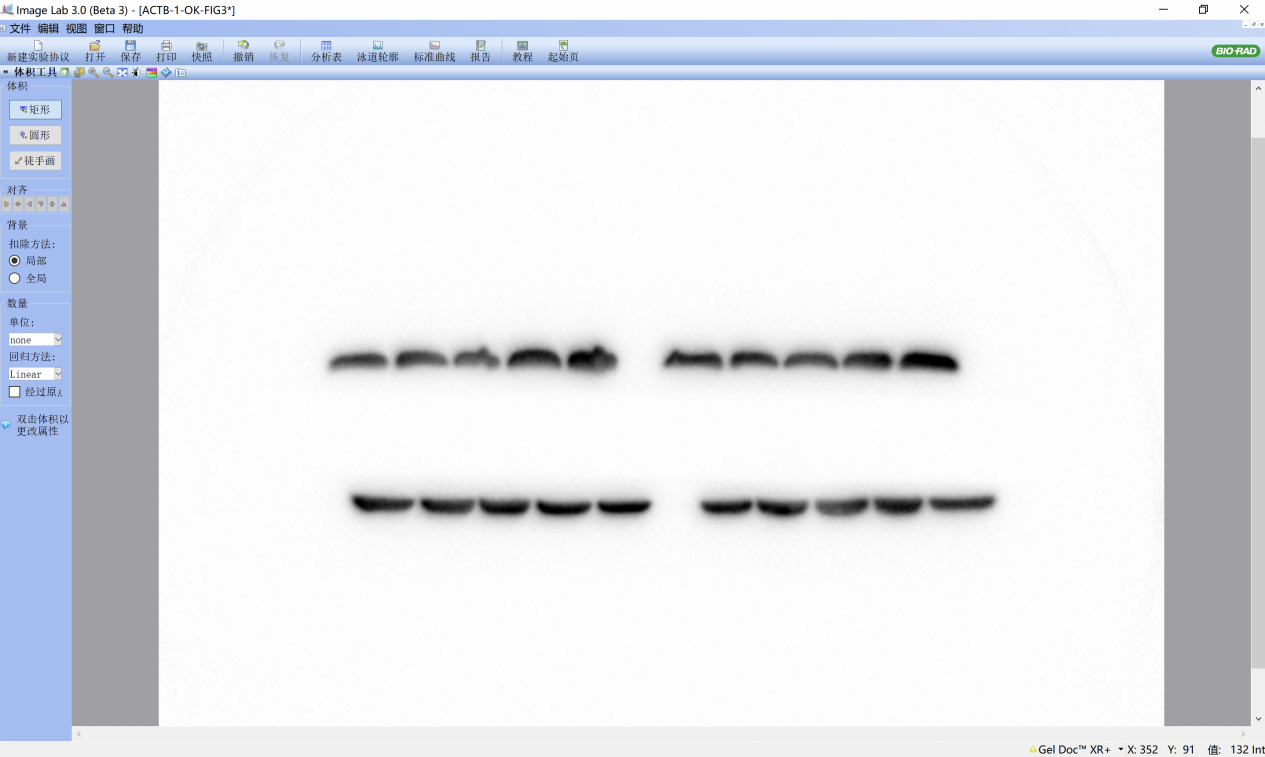


IgG-CoIP-FIG3:


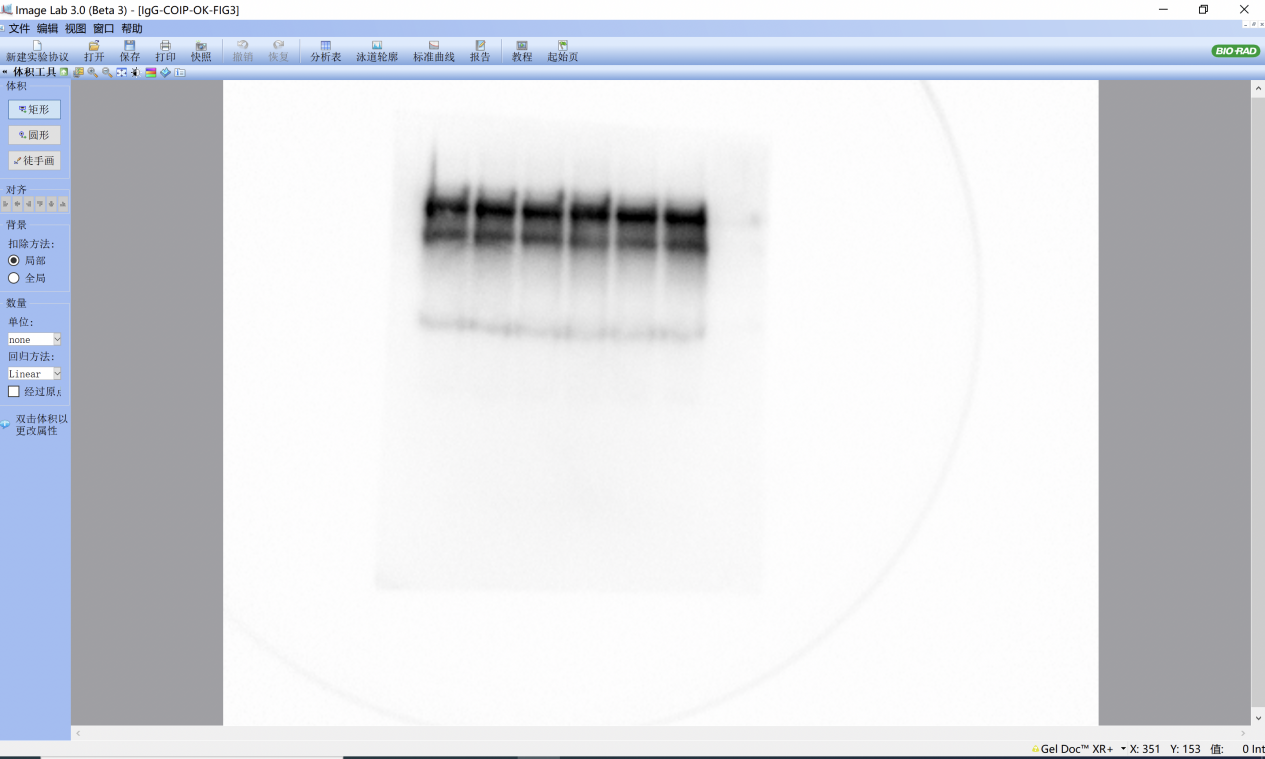


pSMAD1/5-FIG3:


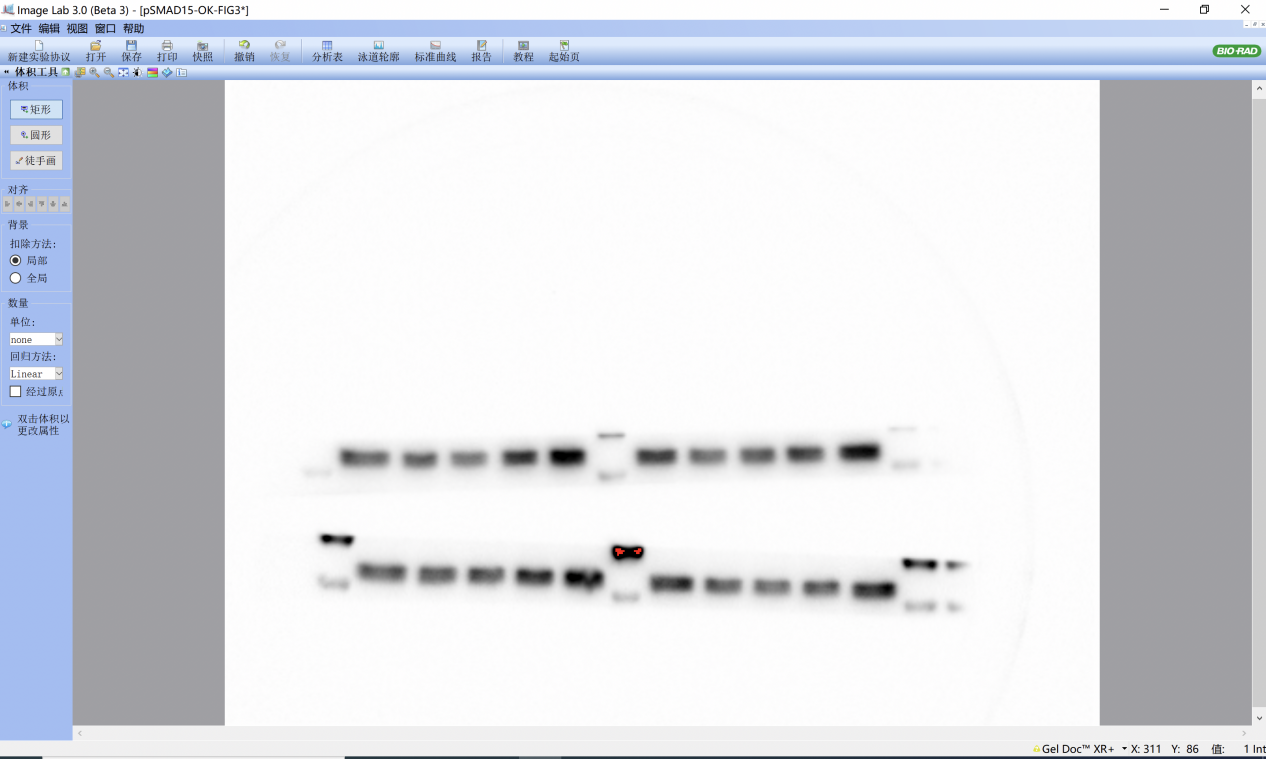


SMAD4-FIG3:


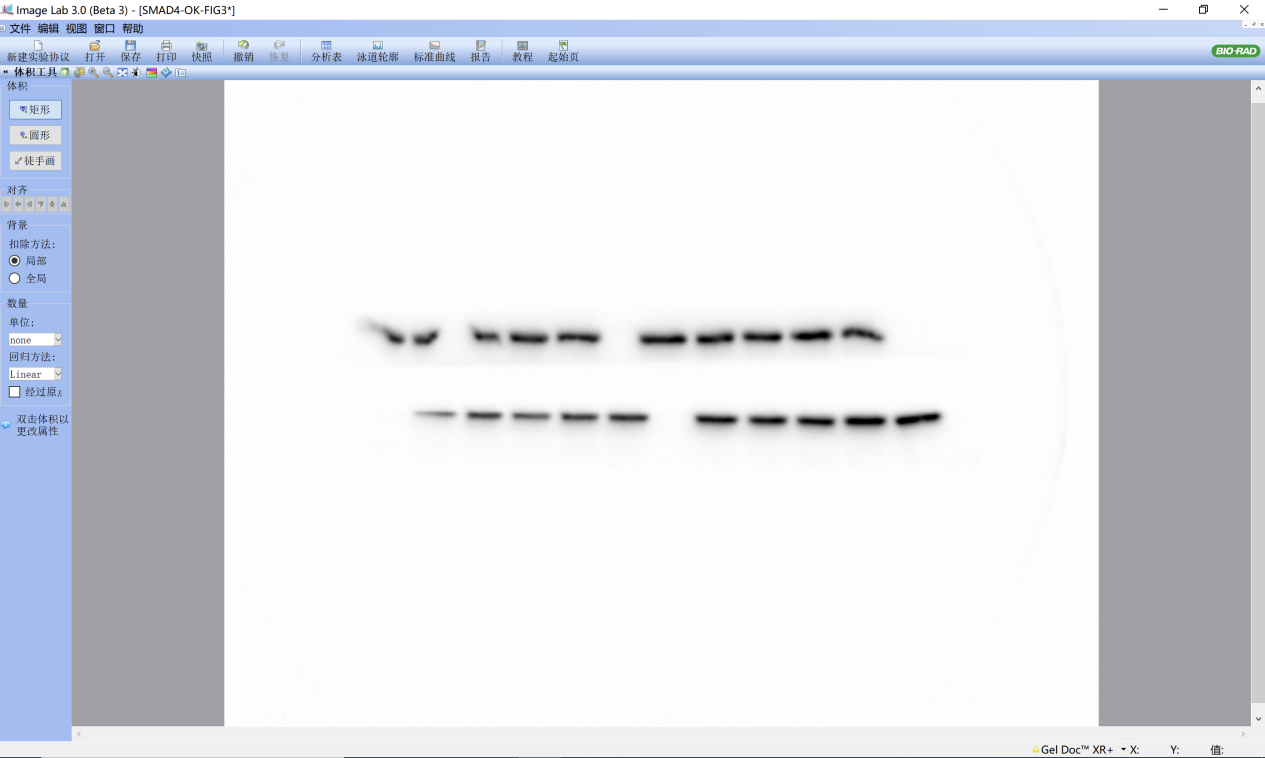


SMAD1/5+SMAD4-CoIP-FIG3:
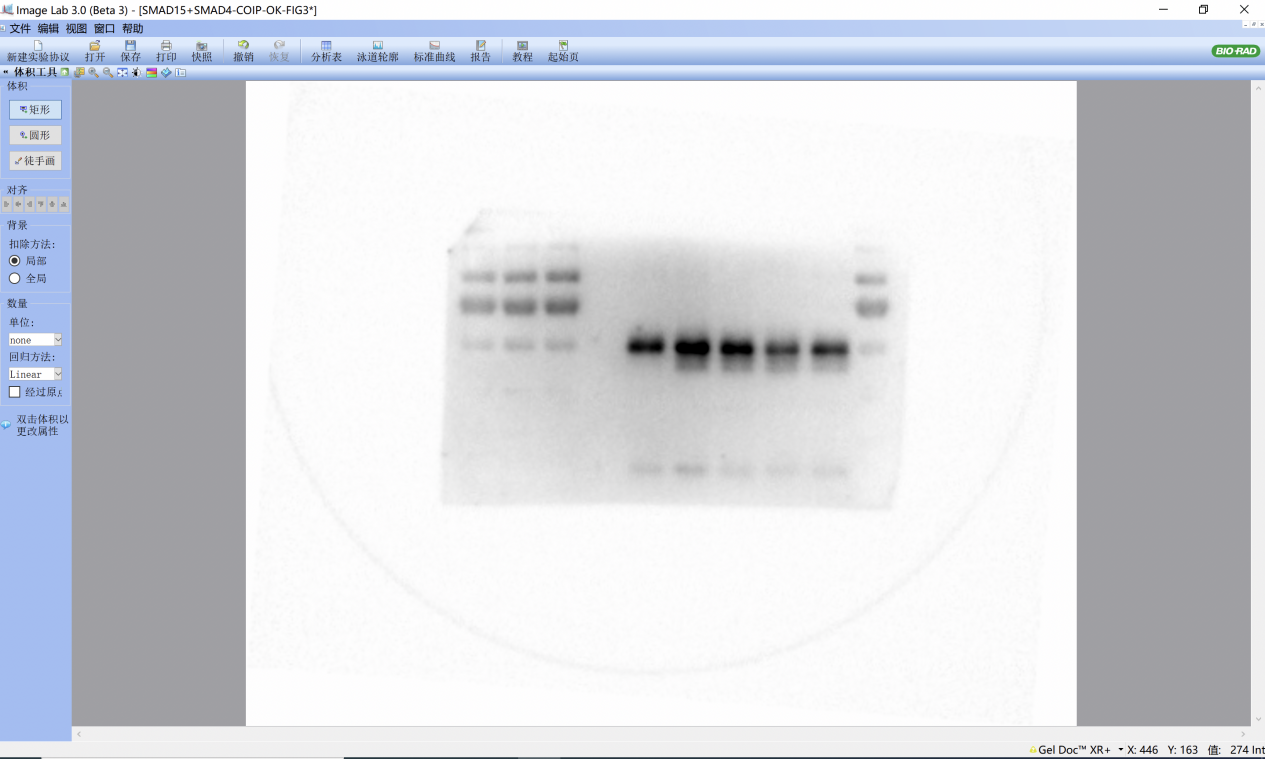


SMAD1/5-FIG3:


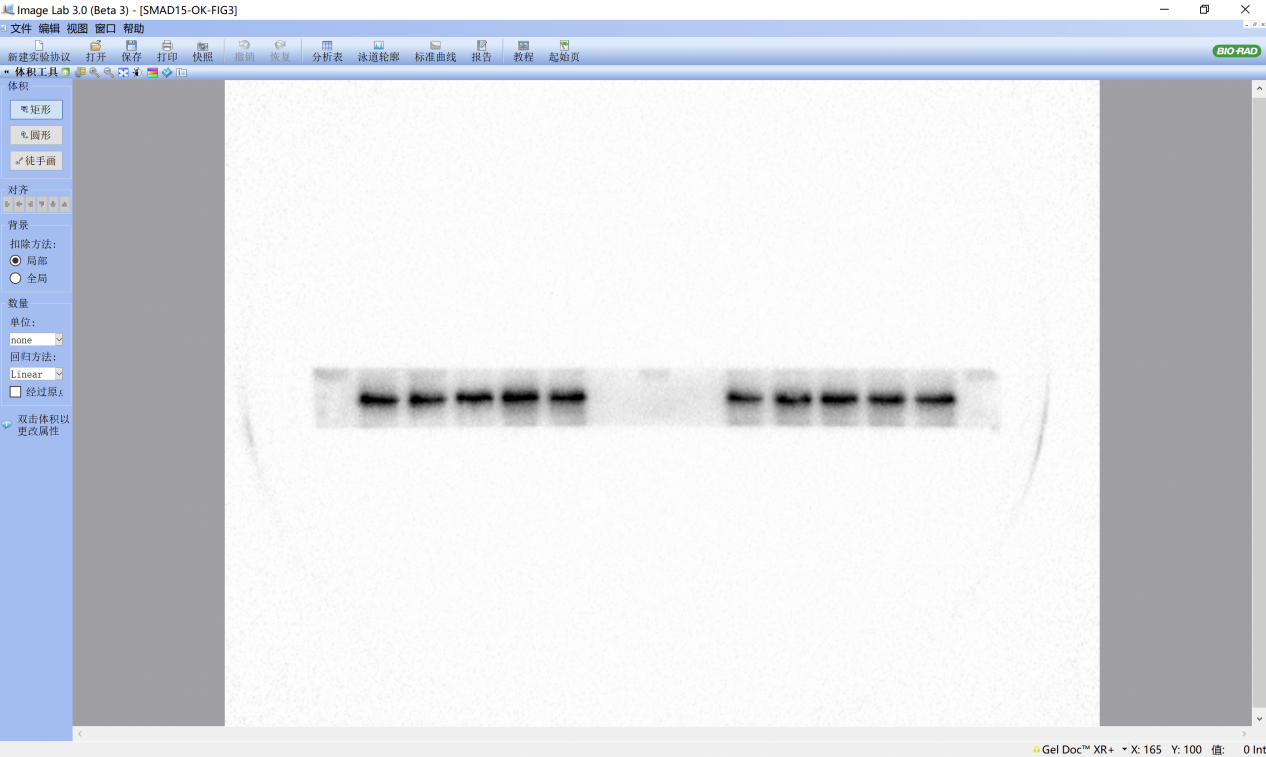


ACTB-FIG5:
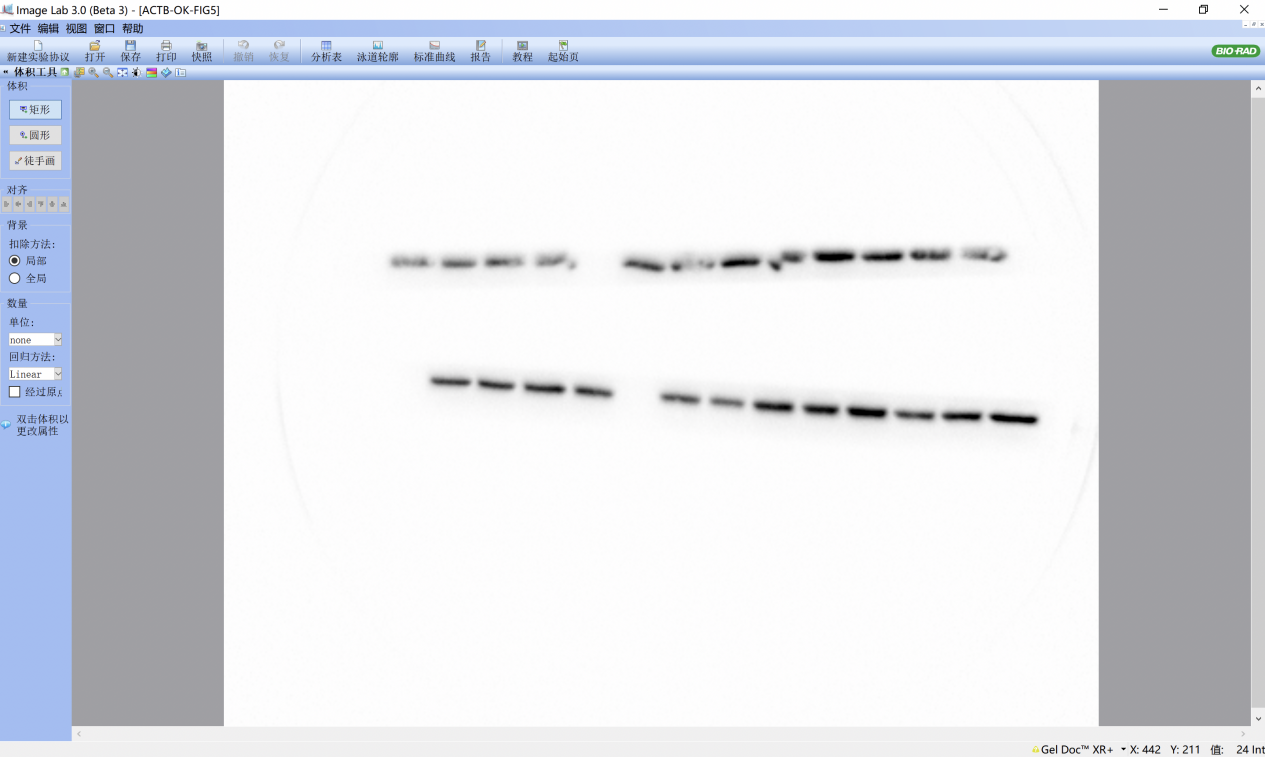


CYP11A1-FIG5:


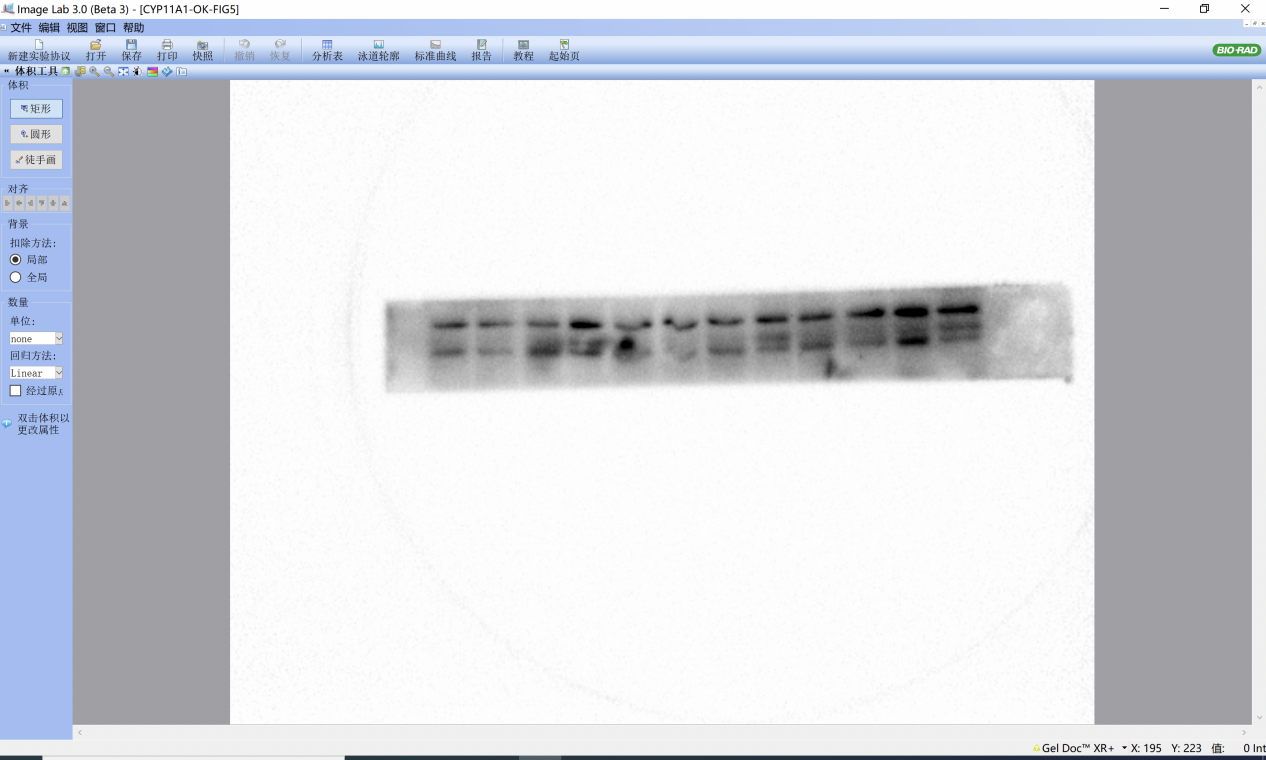


HSD11B1-FIG5:


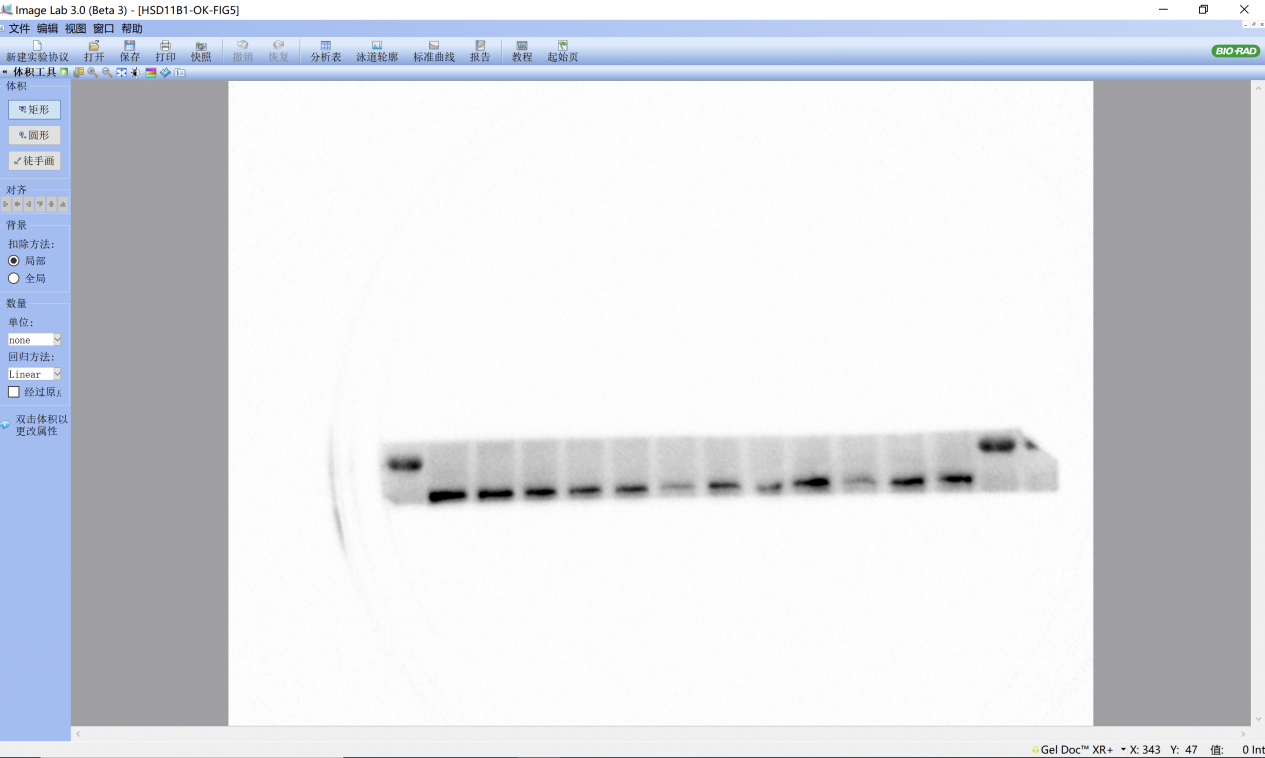


INSL3-FIG5:
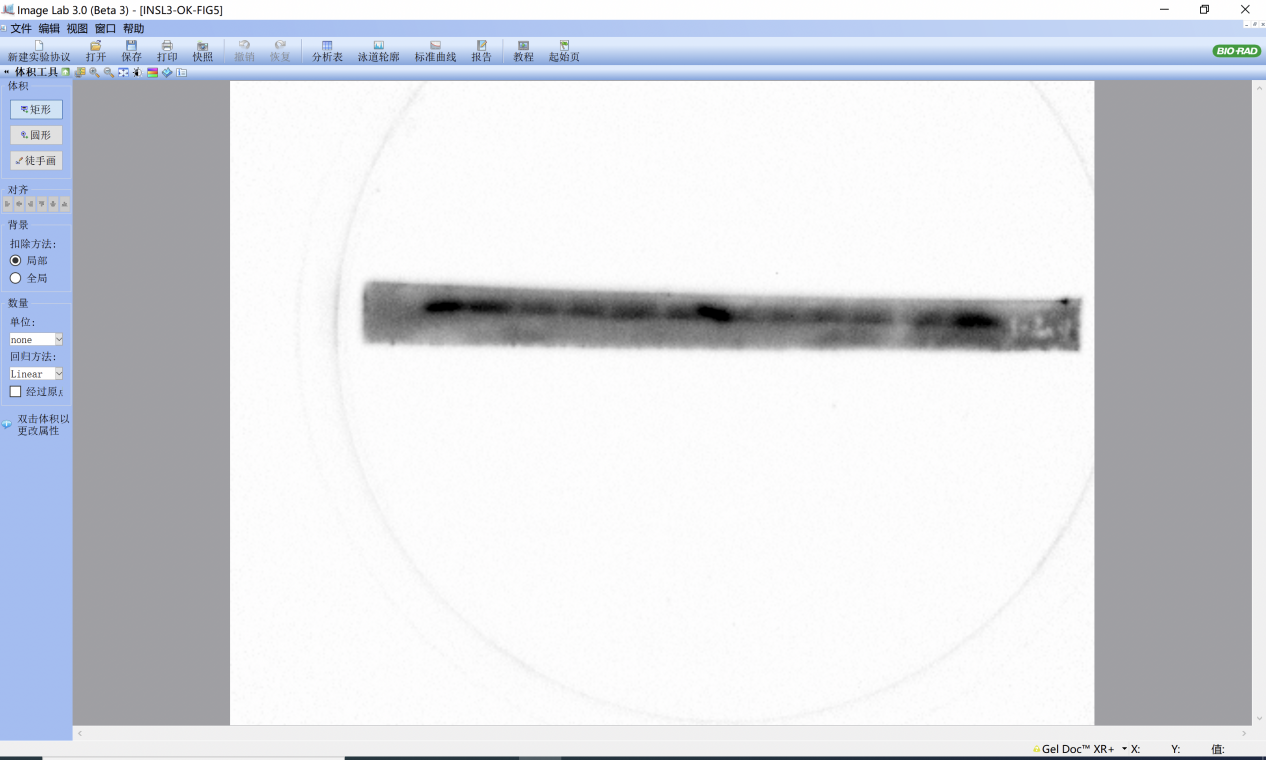


LHCGR-FIG5:
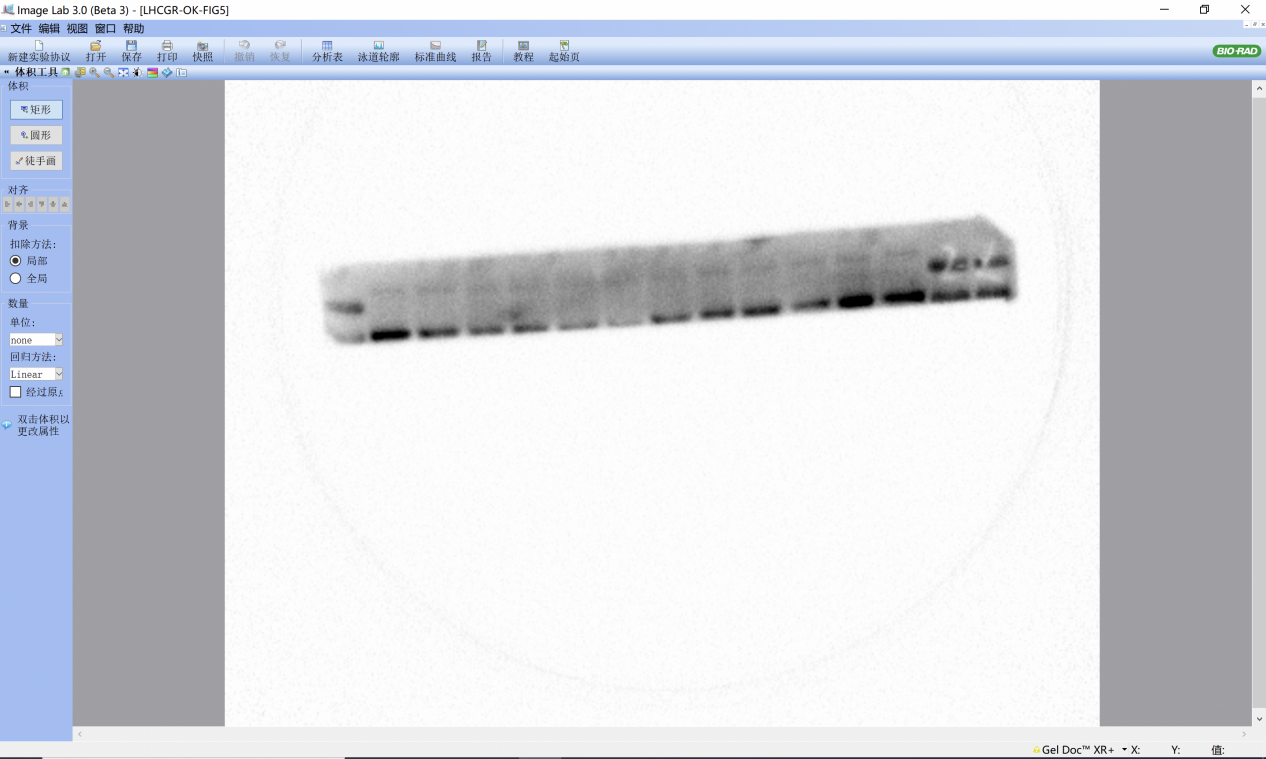


ACTB-FIG6:
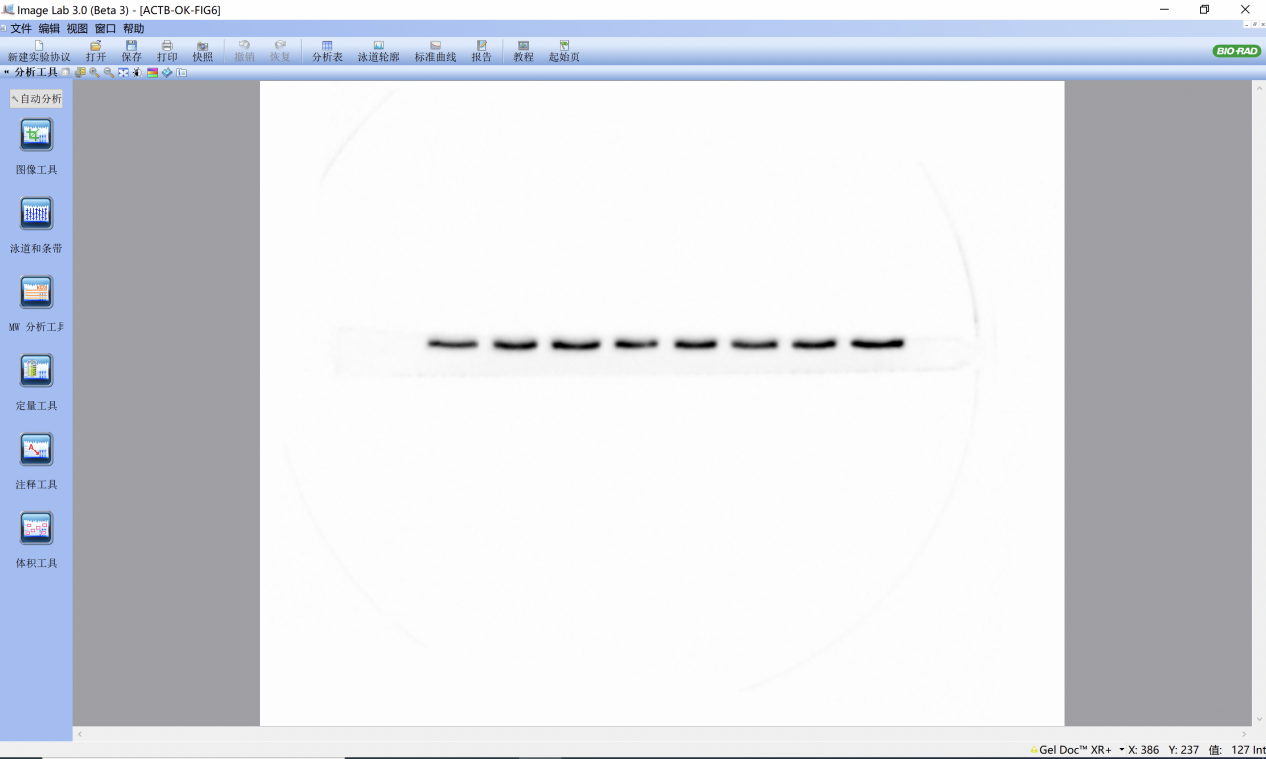


AMPK-FIG6:
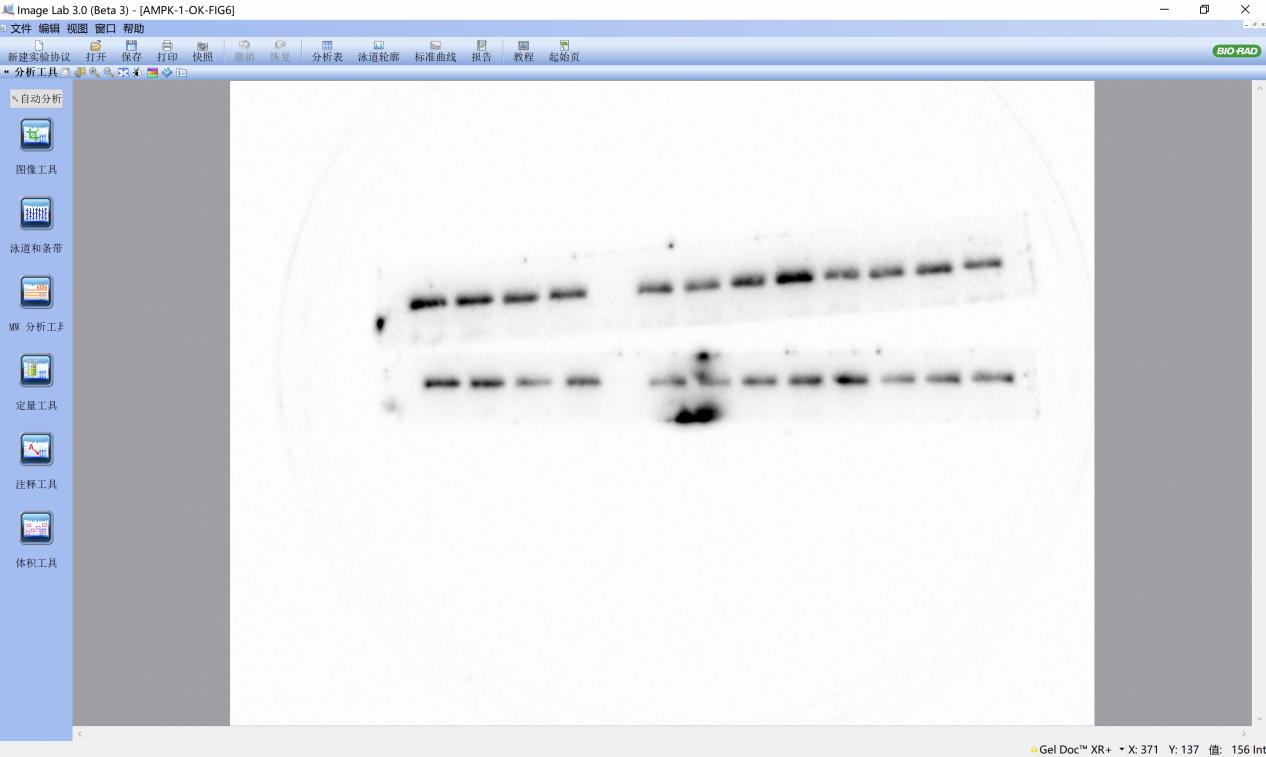


ERK-FIG6:


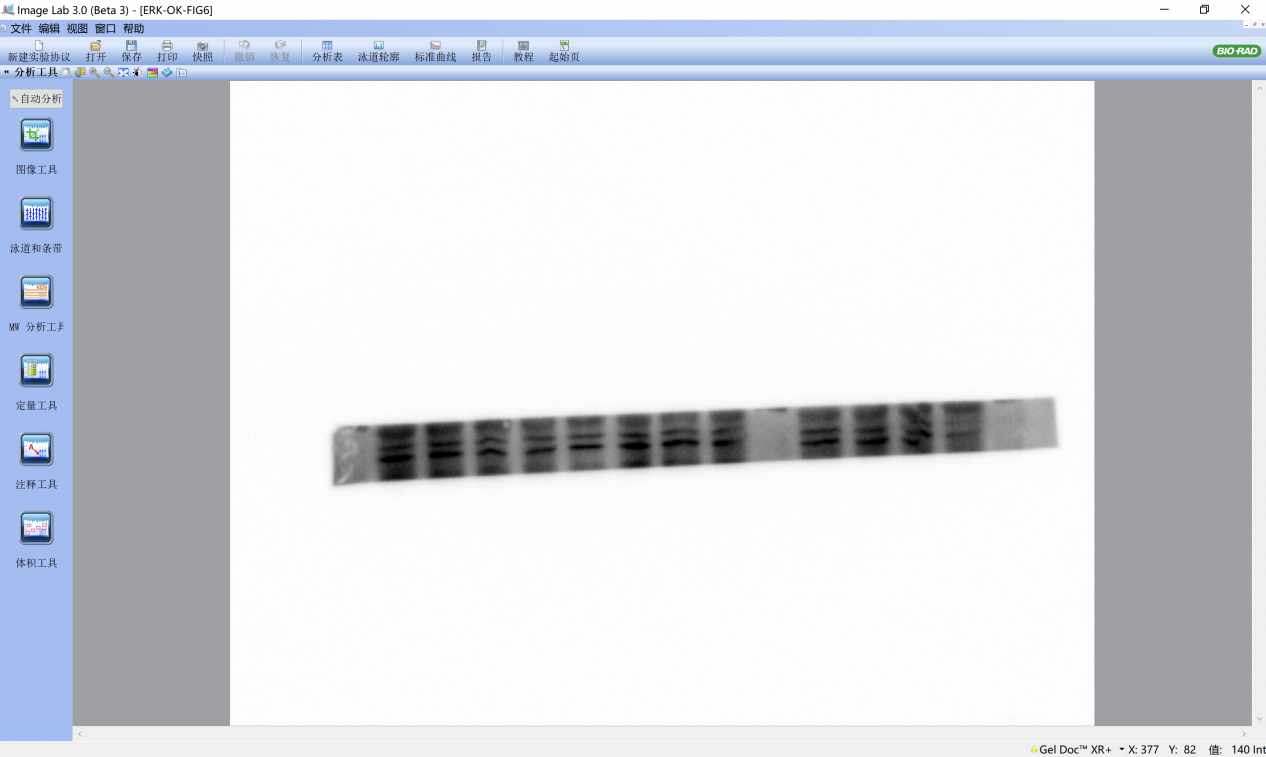


pAMPK-FIG6:
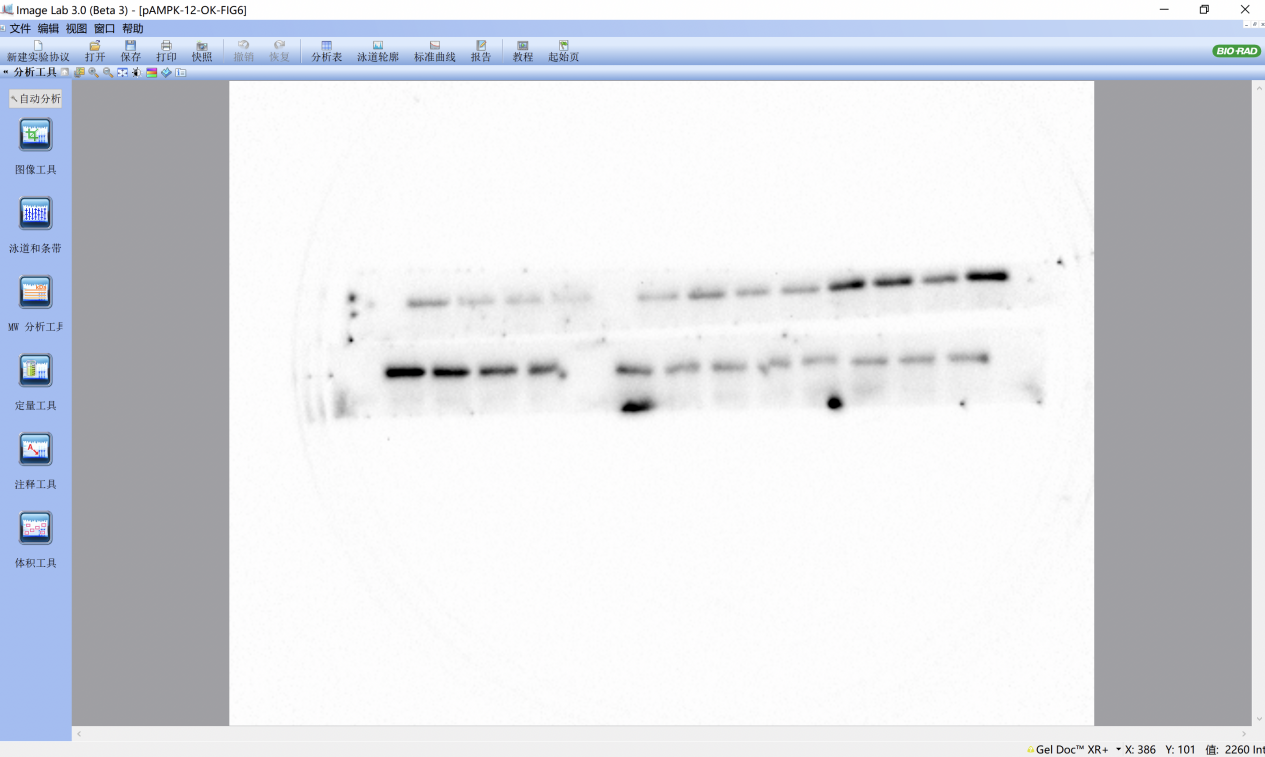


pERK-FIG6:
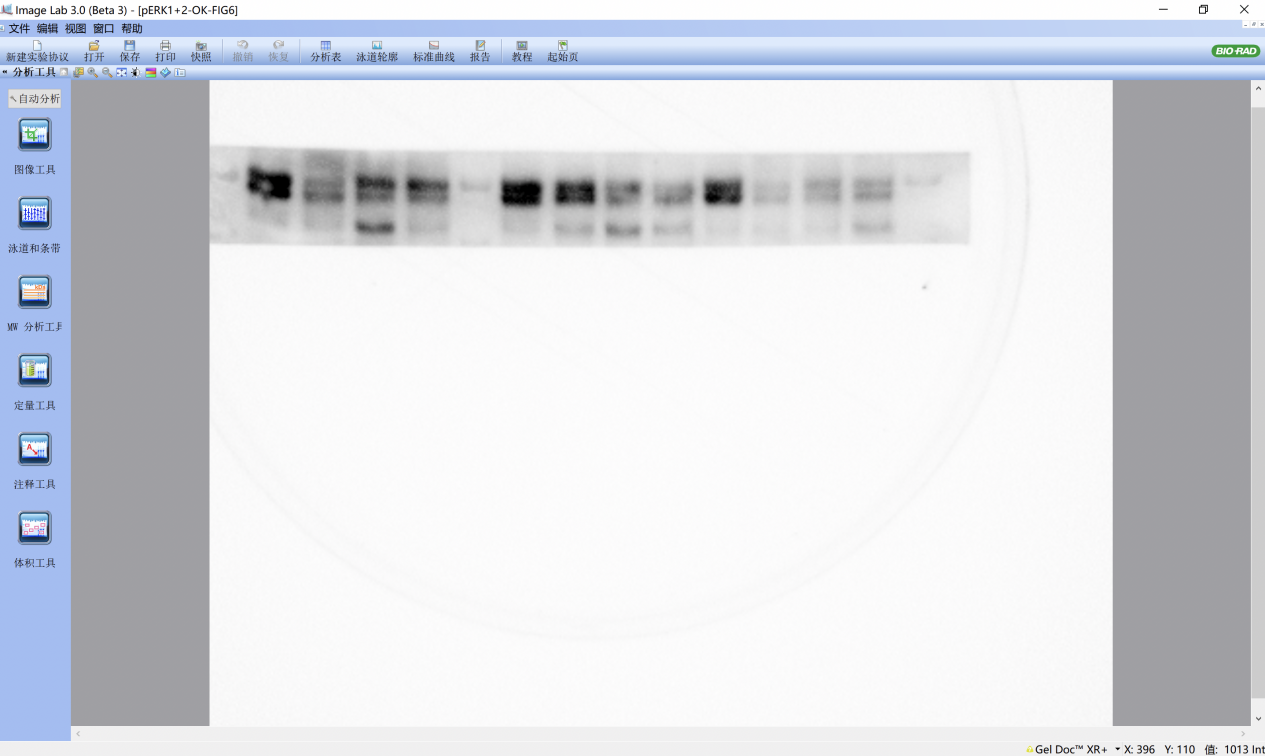


ACTB-FIG8-E:
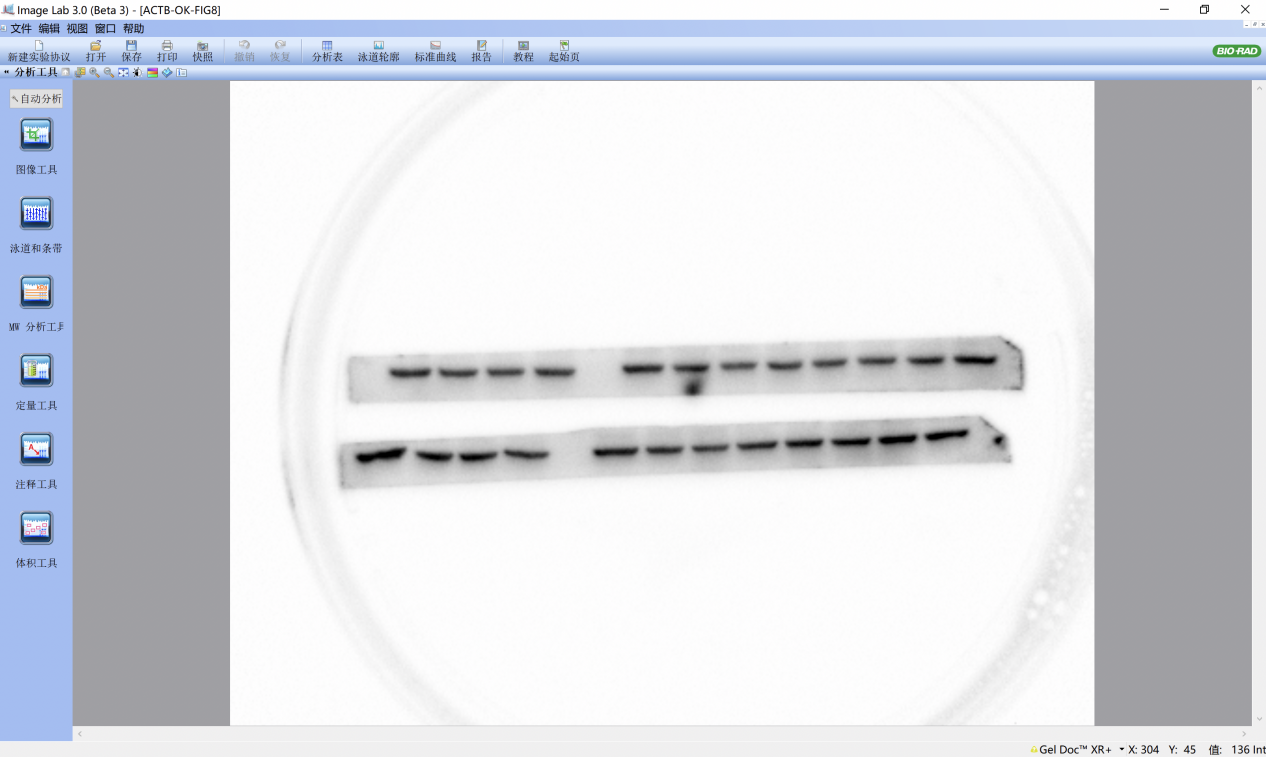


INSL3-FIG8-E:


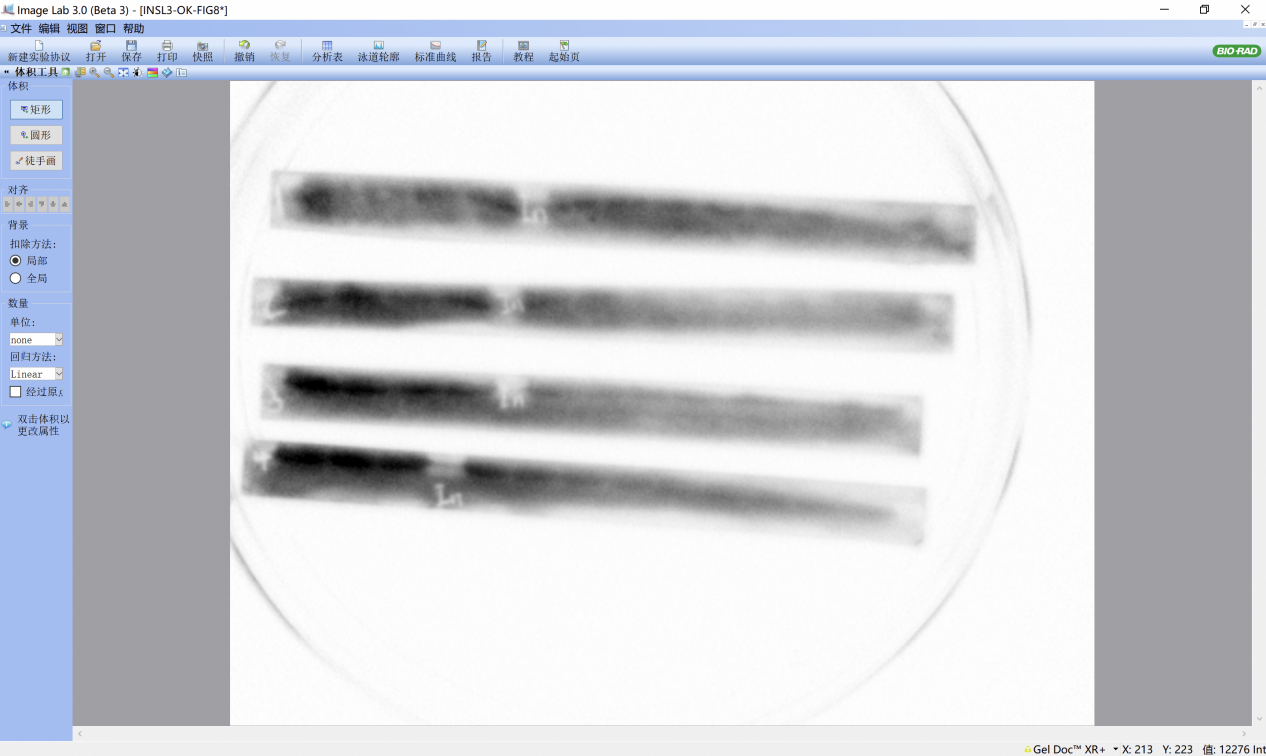


SCARB1-FIG8-E:


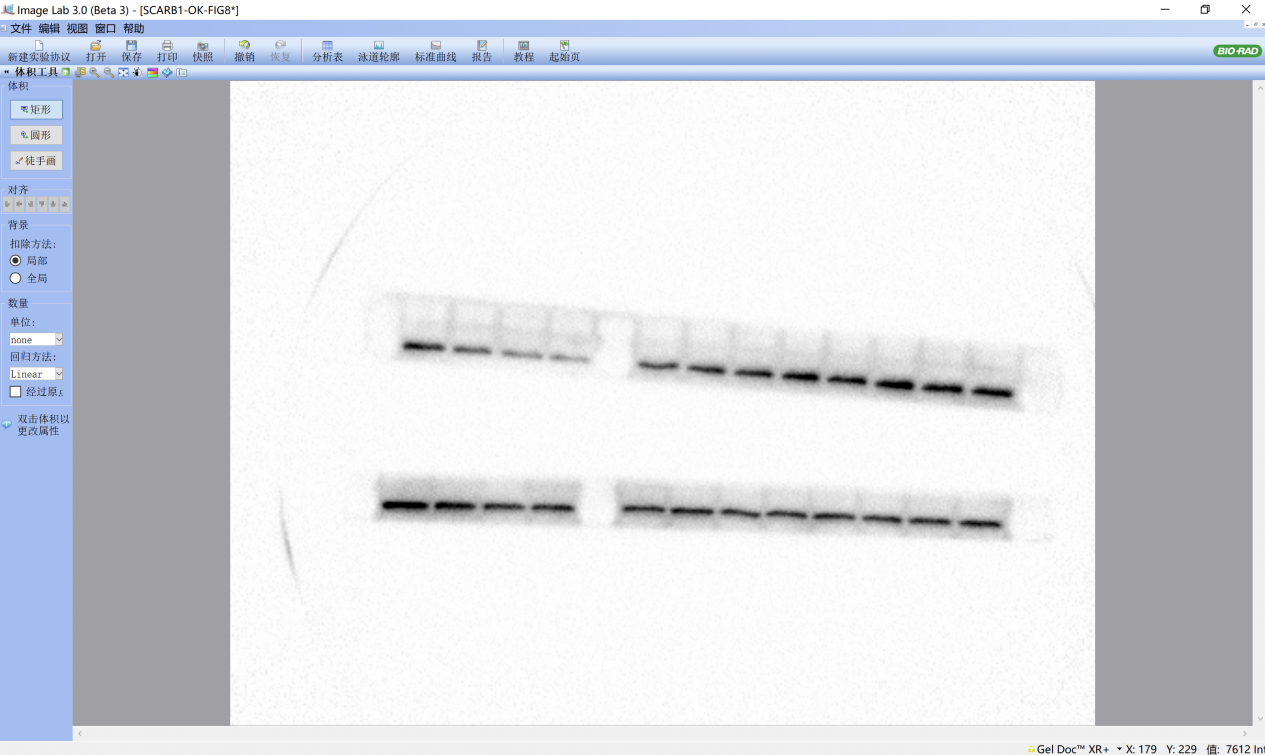


STAR-FIG8-E:


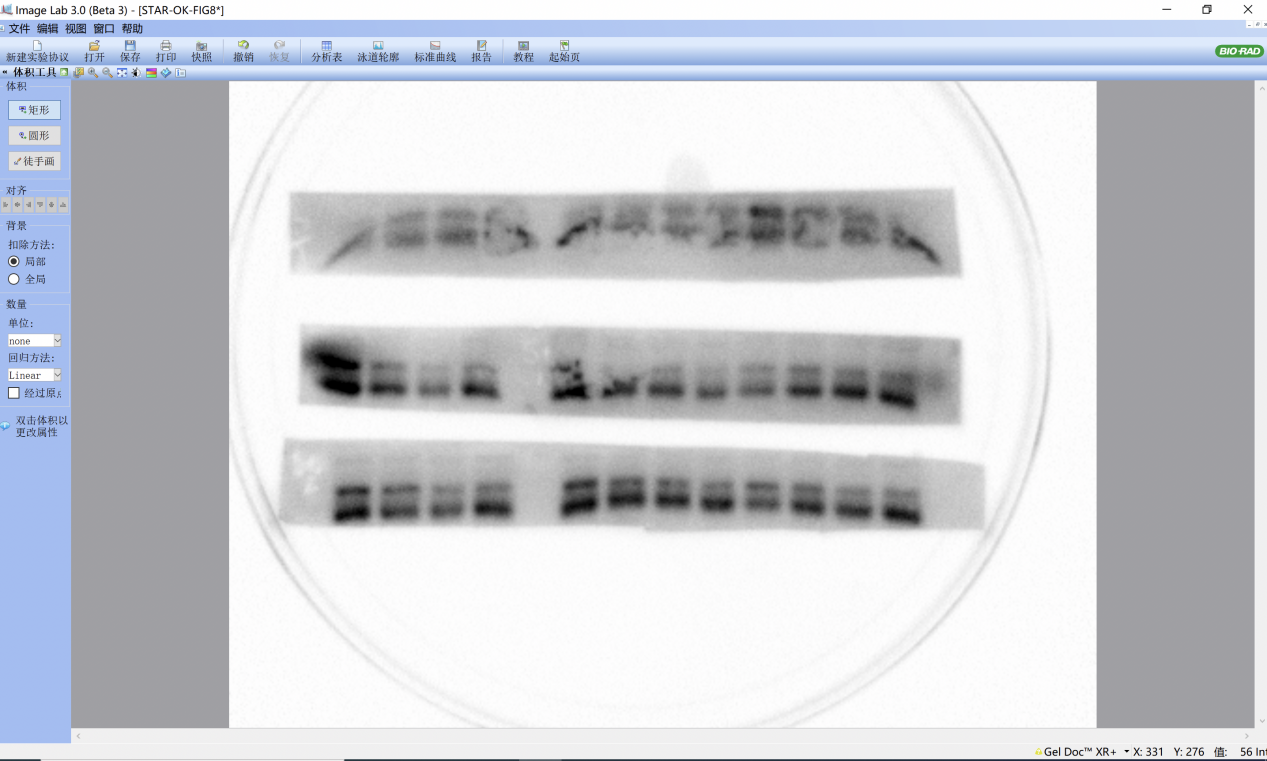


ACTB-FIG8-L:
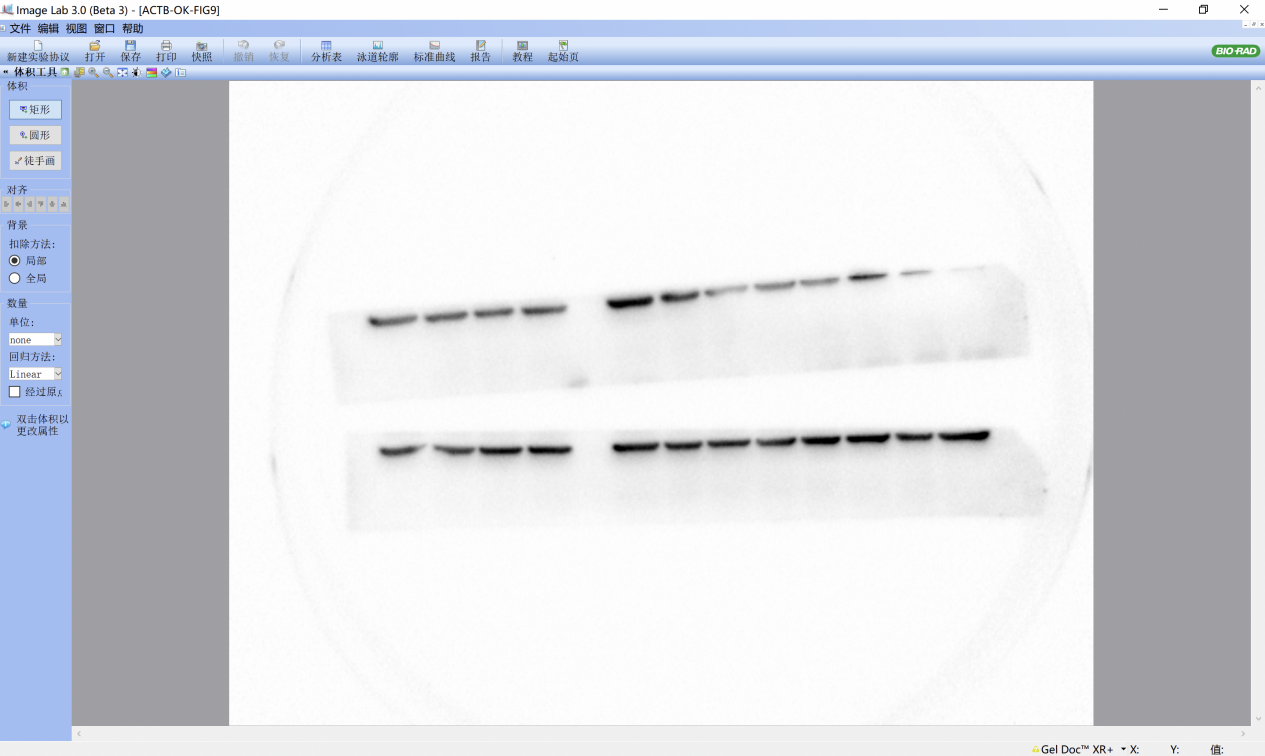


AMPK-FIG8-L:
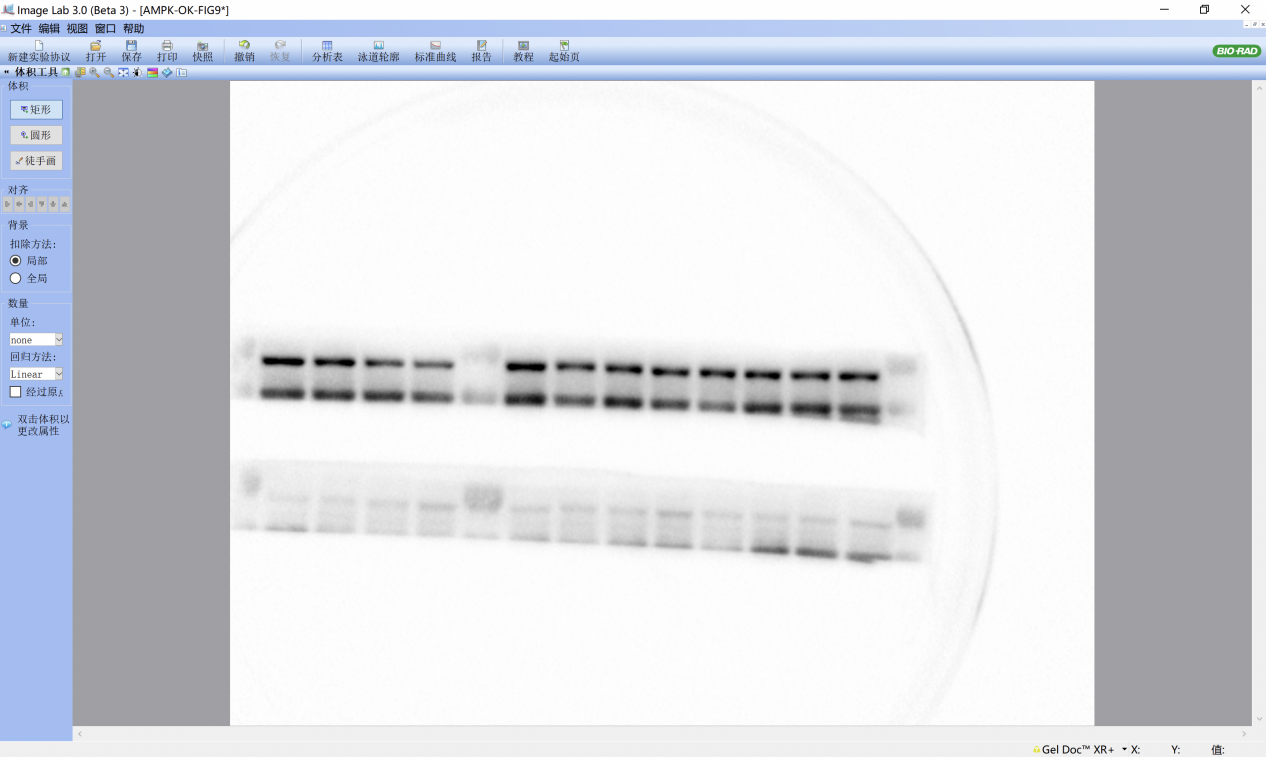


ERK-FIG8-L:


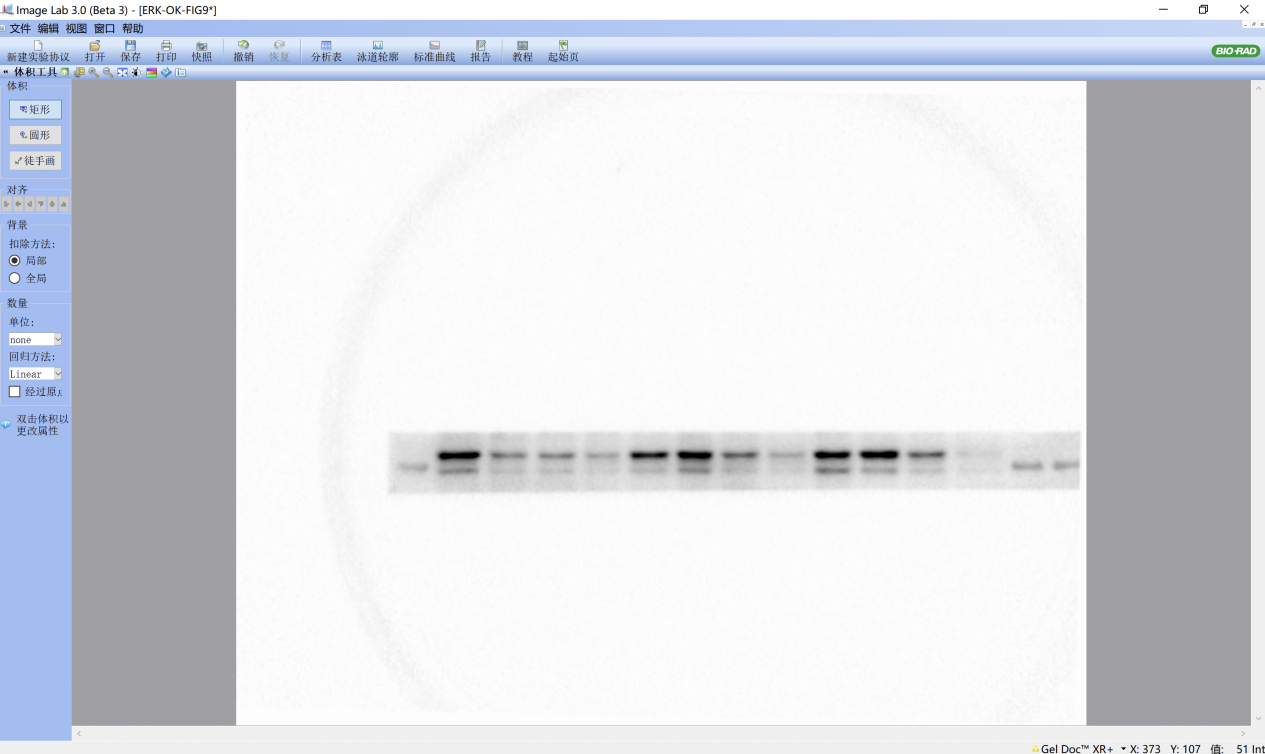


pAMPK-FIG8-L:
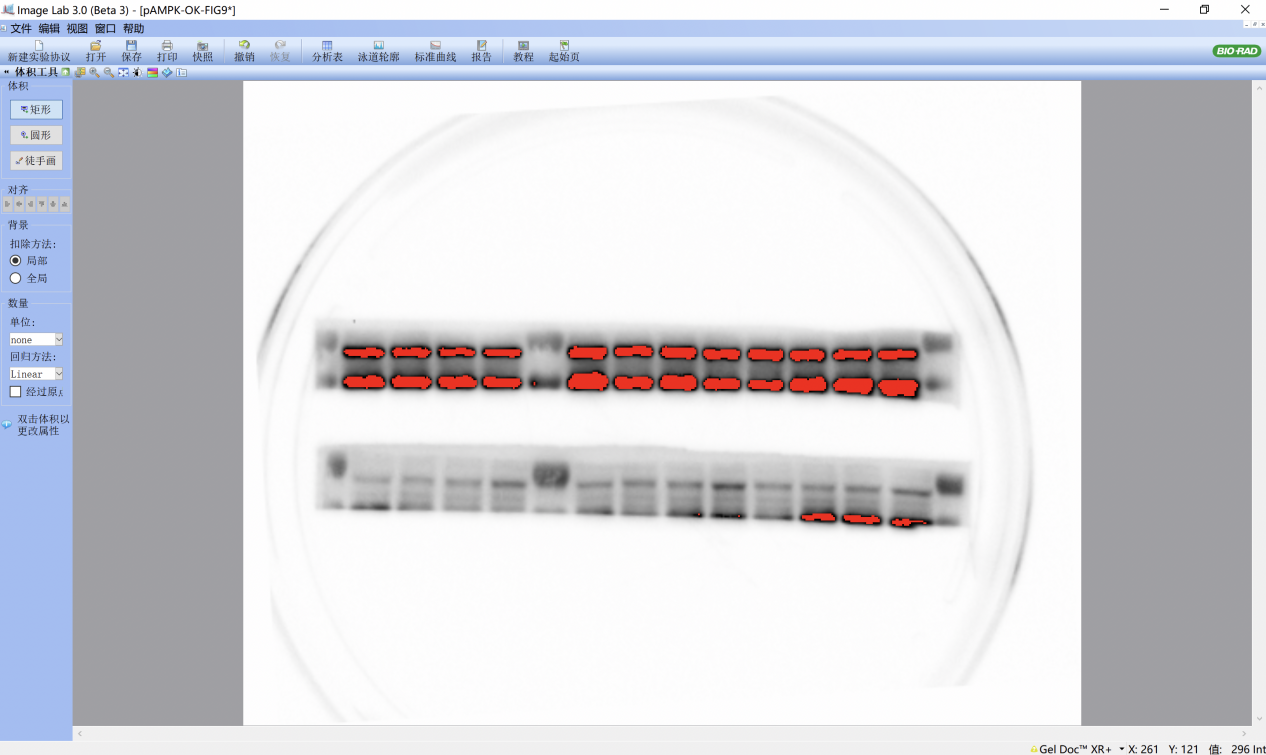


pERK-FIG8-L:
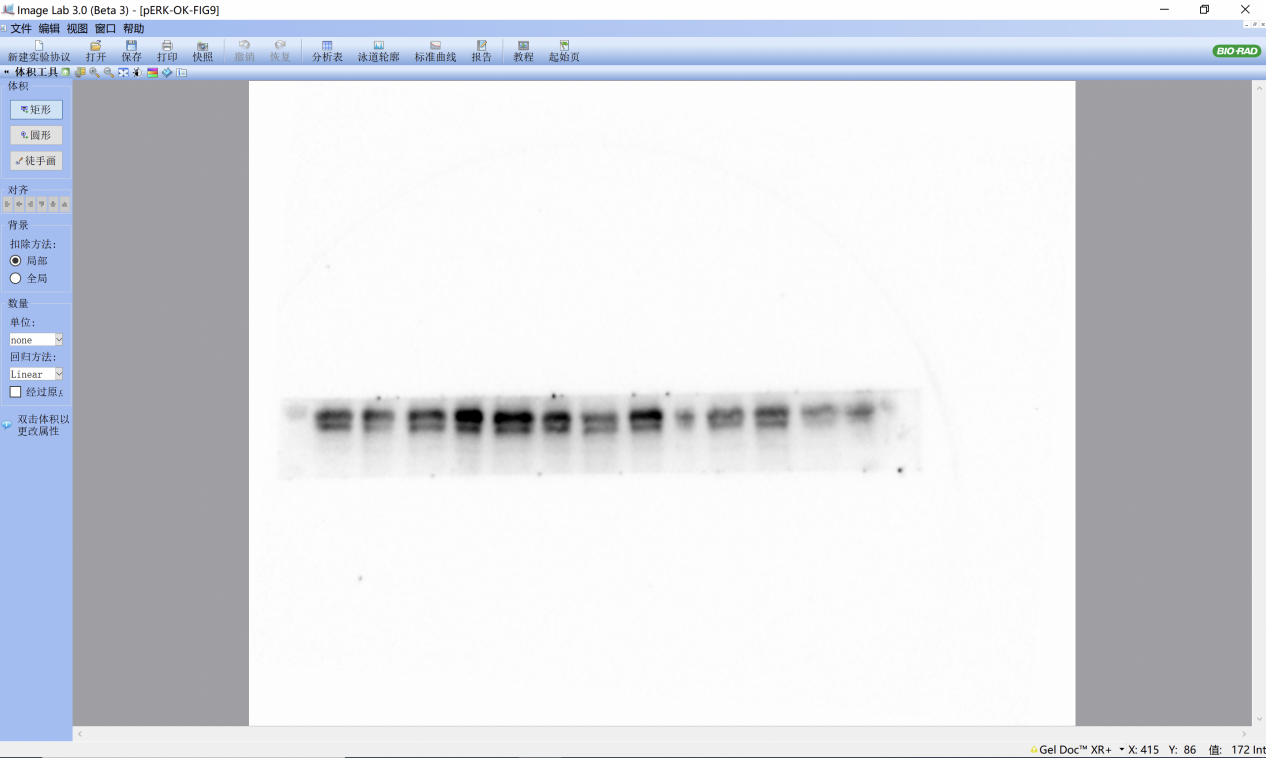


SMAD4-FIG8-L:
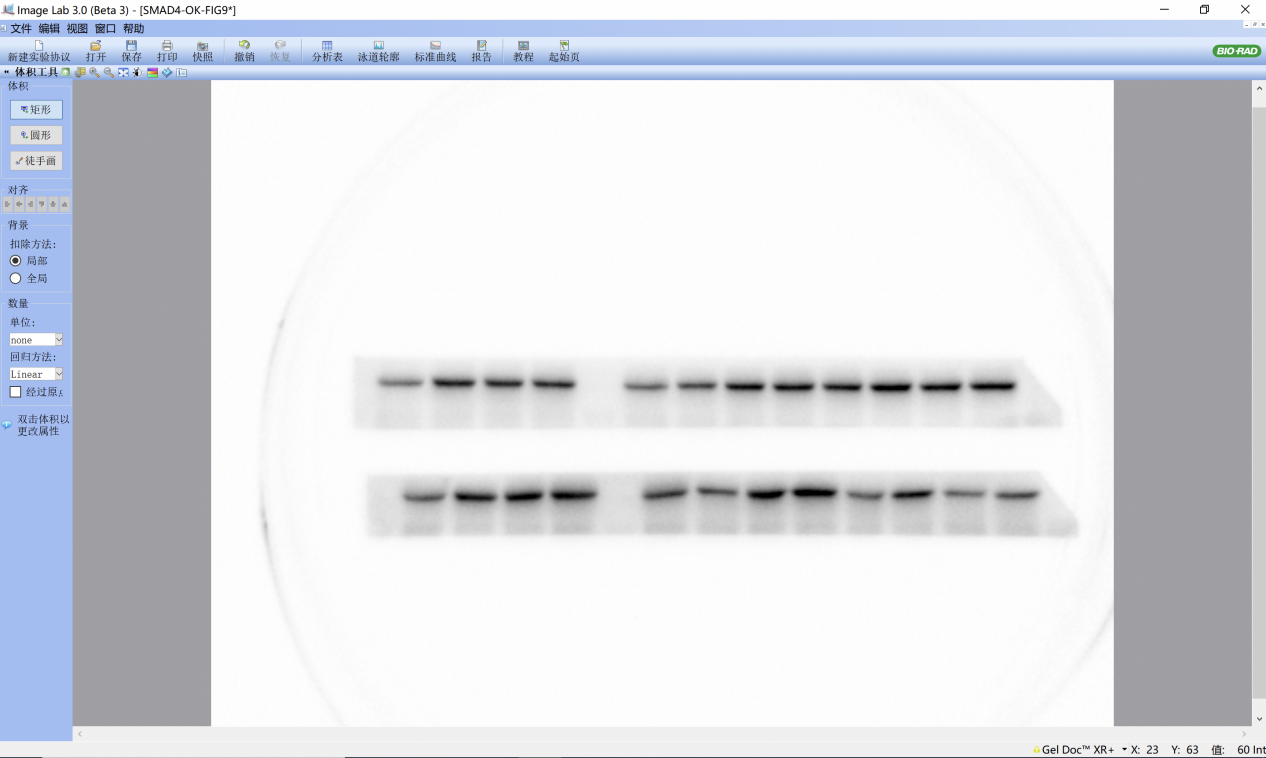


ACTB-FIGS2:
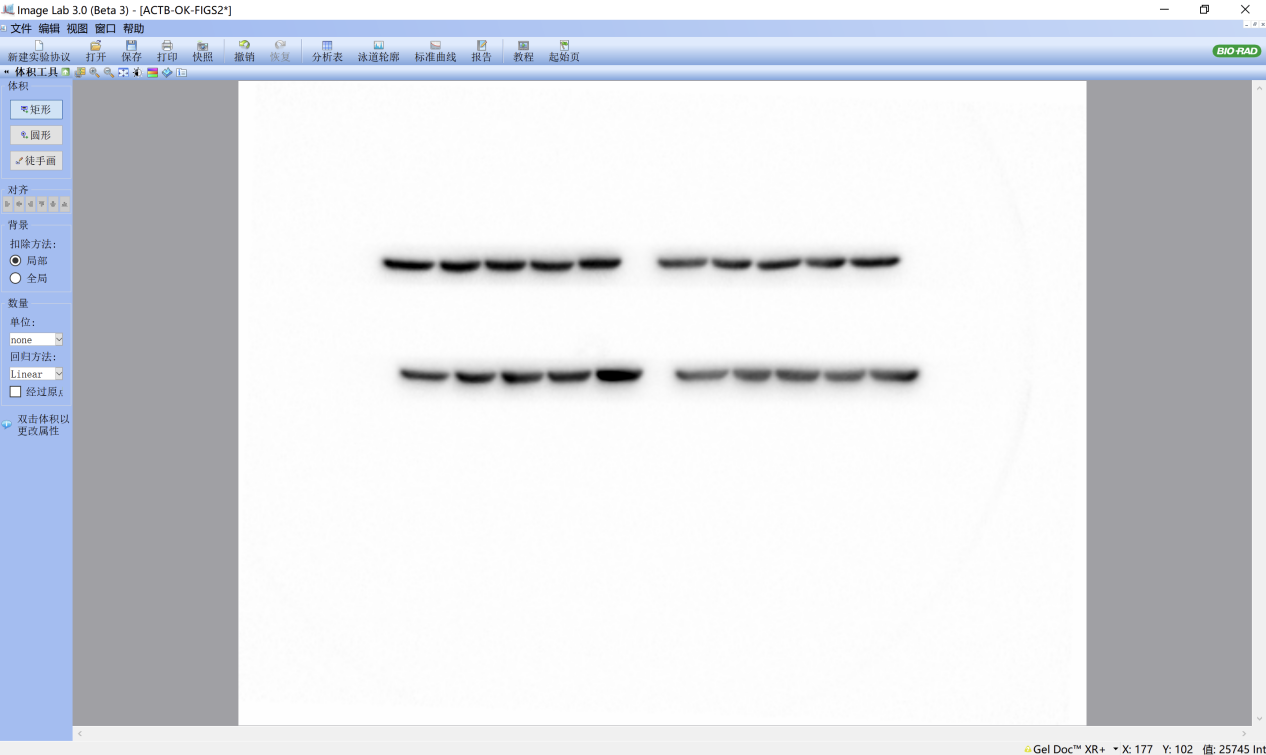


BMPR2-FIGS2:


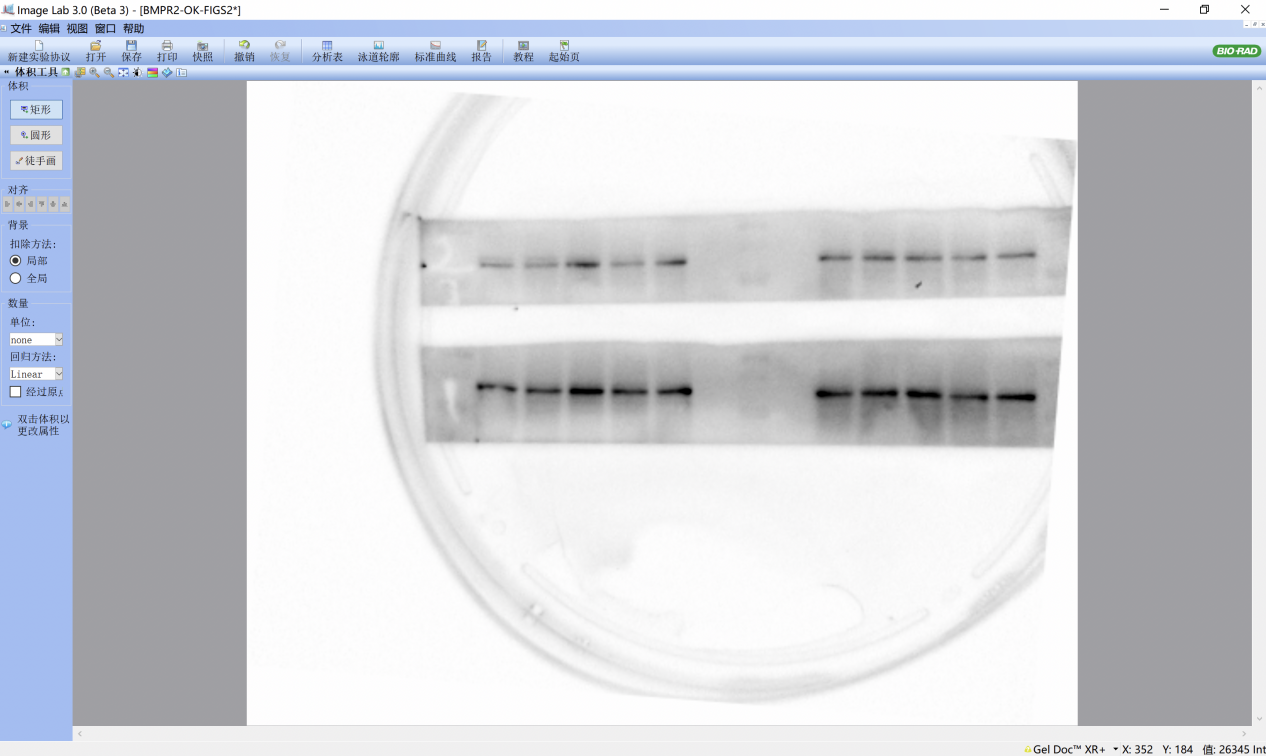


BMPR1B-FIGS2:


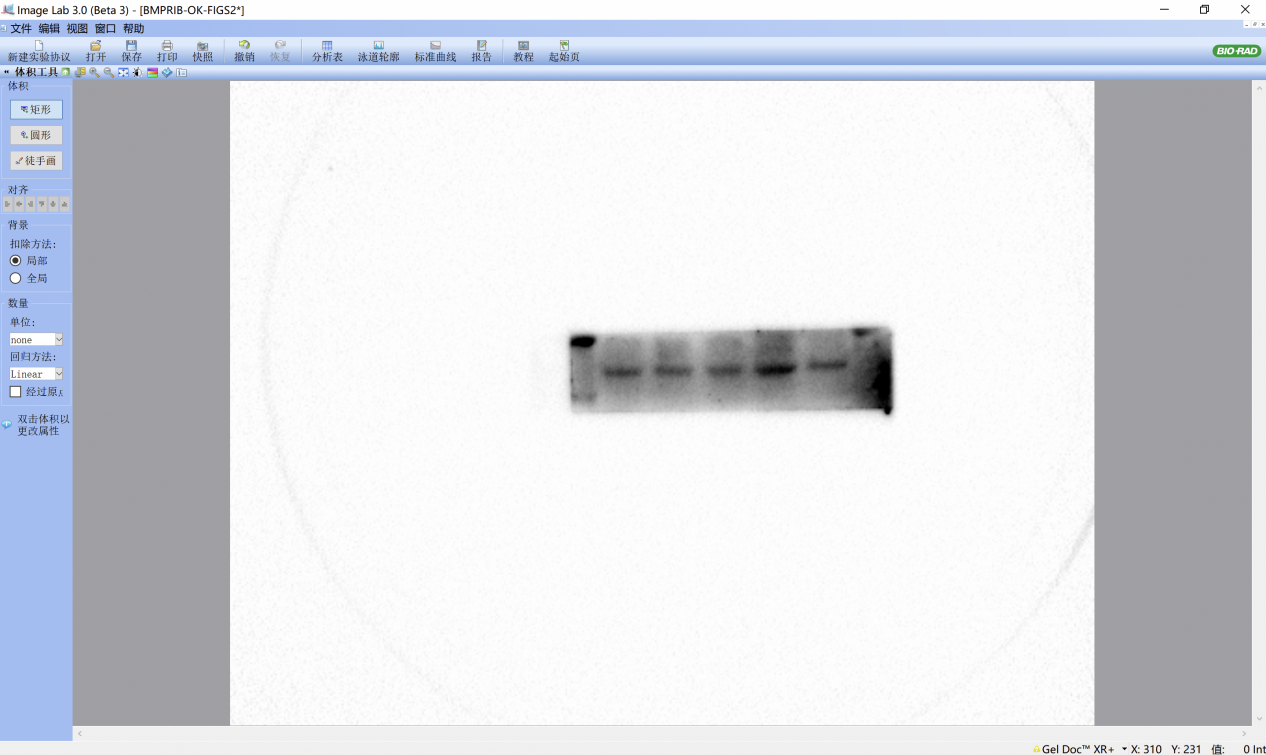

Supplement: Supplementary file 3 — Supplemental Material-2 [file 41419_2022_5471_MOESM3_ESM.docx]
